# Hypoxia stabilizes SETDB1 to maintain genome stability

**DOI:** 10.1093/nar/gkad796

**Published:** 2023-10-18

**Authors:** Sungryul Park, Jin Hwa Cho, Jong-Hwan Kim, Mijin Park, Seulki Park, Seon-Young Kim, Seon-Kyu Kim, Kidae Kim, Sung Goo Park, Byoung Chul Park, Jeong Hee Moon, Gaseul Lee, Sunhong Kim, Jung-Ae Kim, Jeong-Hoon Kim

**Affiliations:** Disease Target Structure Research Center, Korea Research Institute of Bioscience and Biotechnology, Daejeon 34141, Republic of Korea; Disease Target Structure Research Center, Korea Research Institute of Bioscience and Biotechnology, Daejeon 34141, Republic of Korea; Korea Bioinformation Center, Korea Research Institute of Bioscience and Biotechnology, Daejeon 34141, Republic of Korea; Aging Convergence Research Center, Korea Research Institute of Bioscience and Biotechnology, Daejeon 34141, Republic of Korea; Department of Bioscience, University of Science and Technology, Daejeon 34113, Republic of Korea; Disease Target Structure Research Center, Korea Research Institute of Bioscience and Biotechnology, Daejeon 34141, Republic of Korea; Korea Bioinformation Center, Korea Research Institute of Bioscience and Biotechnology, Daejeon 34141, Republic of Korea; Department of Bioscience, University of Science and Technology, Daejeon 34113, Republic of Korea; Aging Convergence Research Center, Korea Research Institute of Bioscience and Biotechnology, Daejeon 34141, Republic of Korea; Department of Bioscience, University of Science and Technology, Daejeon 34113, Republic of Korea; R&D Center, PharmAbcine Inc., Daejeon 34047, Republic of Korea; Disease Target Structure Research Center, Korea Research Institute of Bioscience and Biotechnology, Daejeon 34141, Republic of Korea; Department of Bioscience, University of Science and Technology, Daejeon 34113, Republic of Korea; Department of Bioscience, University of Science and Technology, Daejeon 34113, Republic of Korea; Critical Diseases Diagnostics Convergence Research Center, Korea Research Institute of Bioscience and Biotechnology, Daejeon 34141, Republic of Korea; Core Research Facility & Analysis Center, Korea Research Institute of Bioscience and Biotechnology, Daejeon 34141, Republic of Korea; Core Research Facility & Analysis Center, Korea Research Institute of Bioscience and Biotechnology, Daejeon 34141, Republic of Korea; College of Pharmacy, Chungbuk National University, Cheongju, Chungbuk 28160, Republic of Korea; Drug Discovery Center, LG Chem Ltd., Seoul 07796, Republic of Korea; Disease Target Structure Research Center, Korea Research Institute of Bioscience and Biotechnology, Daejeon 34141, Republic of Korea; Aging Convergence Research Center, Korea Research Institute of Bioscience and Biotechnology, Daejeon 34141, Republic of Korea; Department of Bioscience, University of Science and Technology, Daejeon 34113, Republic of Korea; Disease Target Structure Research Center, Korea Research Institute of Bioscience and Biotechnology, Daejeon 34141, Republic of Korea; Department of Bioscience, University of Science and Technology, Daejeon 34113, Republic of Korea; Graduate School of New Drug Discovery and Development, Chungnam National University, Daejeon 34134, Republic of Korea

## Abstract

Von Hippel–Lindau (VHL) is a tumor suppressor that functions as the substrate recognition subunit of the CRL2^VHL^ E3 complex. While substrates of VHL have been identified, its tumor suppressive role remains to be fully understood. For further determination of VHL substrates, we analyzed the physical interactome of VHL and identified the histone H3K9 methyltransferase SETBD1 as a novel target. SETDB1 undergoes oxygen-dependent hydroxylation by prolyl hydroxylase domain proteins and the CRL2^VHL^ complex recognizes hydroxylated SETDB1 for ubiquitin-mediated degradation. Under hypoxic conditions, SETDB1 accumulates by escaping CRL2^VHL^ activity. Loss of SETDB1 in hypoxia compared with that in normoxia escalates the production of transposable element-derived double-stranded RNAs, thereby hyperactivating the immune-inflammatory response. In addition, strong derepression of TEs in hypoxic cells lacking SETDB1 triggers DNA damage-induced death. Our collective results support a molecular mechanism of oxygen-dependent SETDB1 degradation by the CRL2^VHL^ E3 complex and reveal a role of SETDB1 in genome stability under hypoxia.

## Graphical Abstract

**Graphical Abstract:**
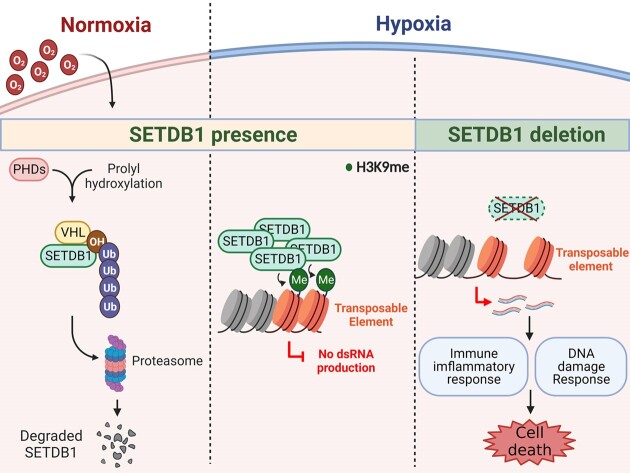

## Introduction

Von Hippel–Lindau (VHL) is the substrate recognition subunit of the E3 complex comprising Cullin2, elongin B/C and RBX1 (CRL2^VHL^) (1). A tumor suppressor role of VHL has been validated based on association of VHL inactivation with a hereditary condition involving formation of tumors in multiple organs (2,3). Inactivation of VHL is considered a hallmark of clear cell renal cell carcinoma (ccRCC) (2). Earlier functional analyses indicate that VHL is involved in cellular oxygen sensing. By recognizing proteins hydroxylated at proline residues, VHL mediates protein ubiquitination by the CRL2^VHL^ complex for subsequent degradation (2). Following the initial identification of hypoxia-inducible factors (HIF) as important substrates of CRL2^VHL^ (4), the list of CRL2^VHL^ targets continues to expand, providing further insights into the molecular mechanisms underlying the tumor suppressive functions of VHL.

Hypoxia is an important microenvironment factor that determines cell fate by affecting proliferation, mobility, differentiation and death (5–7). HIF are master regulators that trigger the hypoxia response (8). While α-subunits of HIF heterodimers, HIF1α and HIF2α (HIF1/2α), are hydroxylated at proline residues by prolyl hydroxylase domain proteins (PHDs) and subsequently undergo CRL2^VHL^-dependent proteasomal degradation in normoxia, oxygen depletion stabilizes HIF1/2α, thereby inducing HIF-dependent transcriptional reprogramming (4). In addition to HIF-dependent pathways, hypoxia induces HIF-independent responses, including epigenetic changes associated with histone modifications and DNA methylation (9,10). Given that the Jumonji C-domain-containing family of histone lysine demethylases (KDM) and ten-eleven translocation proteins (TET) involved in DNA demethylation use oxygen as a co-substrate, under lack of oxygen, the catalytic activities of these enzymes are lost, affecting methylation of histones and DNA (11).

SETDB1 is an evolutionarily well-conserved H3K9 methyltransferase. Frequent amplification of SETDB1 in various tumor types (including melanoma, lung, colon, breast and liver cancers (12–16)) and SETDB1 deletion-associated defects in tumor growth support its function as a prominent cancer driver (17). SETDB1-mediated H3K9 methylation is involved in repression of transposable elements (TE) as well as a specific set of protein-encoding genes (18). TEs are classified into long terminal repeat (LTR)-containing endogenous retroviruses (ERV), non-LTR–containing long interspersed nuclear elements (LINE) and short interspersed nuclear elements (SINE). Transcripts derived from TEs generate endogenous double-stranded RNAs (dsRNAs) that threaten genome stability (19). In addition, ‘viral mimicry’ of ERV transcripts is known to activate antiviral signaling pathways related to the innate immune response (20).

Here, we identified SETDB1 as a novel substrate of the CRL2^VHL^ complex based on analysis of the physical interactome of VHL. Mechanistic studies disclosed that SETDB1 stability is regulated in an oxygen-dependent manner. Functional analyses showed that SETDB1 stabilization is essential for TE repression in hypoxia to prevent hyperactivation of the immune inflammatory response and DNA damage-induced cell death. Our results collectively indicate a critical role of hypoxia-induced SETDB1 stabilization in maintaining genome stability, providing an insight into the mechanisms by which cells epigenetically maintain homeostasis under conditions of hypoxic stress.

## Materials and methods

### Plasmids

HA-VHL and Myc-VHL were generated by cloning human VHL cDNA into N-HA pcDNA3.1 and Myc-pcDNA3.1 vectors, respectively. Human SETDB1 cDNA was subcloned into N-Flag pcDNA3.1, C-Flag pXY and C-6XhisSBP pXY vectors. EGLN2 and EGLN3 cDNA were purchased from Korean Human GeneBank (KHGB). PCR-amplified EGLN2 and EGLN3 cDNAs were subcloned into N-Flag pcDNA3.1. PHD2-EGFP was purchased from Addgene (Watertown, MA, USA) (plasmid no. 21401) and Flag-PHD4 generated by cloning human PHD4 cDNA into N-Flag pcDNA3.1. The truncation mutants of Flag-SETDB1, T1 (amino acids 1–555), T2 (amino acids 1–668), T3 (amino acids 711–1290), T4 (amino acids 556–801), T5 (amino acids 802–1290) and T6 (amino acids 556–801) were generated by cloning the corresponding human cDNA into N-Flag pcDNA3.1.

All point mutations were generated by site-directed mutagenesis using a rapid PCR-based method. All generated constructs were verified by DNA sequencing.

### Cell culture

All cell lines used in the study were authenticated, *Mycoplasma* free and commercially available. The Mycoplasma test was conducted using an e-Myco™ Mycoplasma PCR Detection Kit (25235, iNtRON Biotechnology, Kyonggi‐Do, South Korea). Cells were cultured in Dulbecco's modified Eagle's medium (HeLa; CCL-2, HEK293; CRL-1573, HEK293T; CRL-3216) or RPMI1740 (SW480; CCL-228, SW620; CCL-227) supplemented with 10% fetal bovine serum and antibiotic-antimycotic (catalog no. 15240062, Gibco, Waltham, MA, USA). Cells were maintained at 37°C and 5% (v/v) CO_2_ in a humidified incubator Panasonic, catalog no. MCO-19AIC). For hypoxia exposure, cells were cultured in a humidified O_2_/CO_2_ incubator (Panasonic, catalog no. MCO-5M-PK) at 37°C and 5% (v/v) CO_2_ under the indicated O_2_ concentrations. All the cell lines specified here were purchased from the ATCC. The human embryonic kidney a (HEKa) cell line (C0055C, Thermo Fisher, Waltham, MA, USA) was cultured in EpiLife medium (catalog no. MEPI500CA, Thermo Fisher) with HKGS (catalog no. S0015, Thermo Fisher) according to the manufacturer's protocols. HDFn cells (catalog no. C0045C, Thermo Fisher) were cultured in Medium 106 (catalog no. M106500, Thermo Fisher) supplemented with LSGS (catalog no. S003K, Thermo Fisher).

### Lentiviral vectors and stable cell lines

For generation of shRNA and Cas9–sgRNA lentiviral vectors, shRNAs targeting CDS of human SETDB1 and non-targeted control were cloned into pLKO.1 (no. 8453, Addgene) plasmid and shRNA targeting 3′ untranslated region of human SETDB1 was cloned into pLKO.1-blast (no. 26655, Addgene) using oligonucleotides synthesized by Bioneer (Daejeon, South Korea). sgRNAs targeting human SETDB1 were subcloned in pL-CRISPR.SFFV.tRFP (no. 57826, Addgene) using annealed oligonucleotides from Bioneer. Sequences are presented in Supplementary Table S1. For generation of SETDB1 and VHL expressing lentiviral vectors, Flag-tagged SETDB1 and Flag-tagged VHL were subcloned into pLVX-EF1α-IRES-Puro (catalog no. 631253, Clontech).

For generation of lentiviral particles, HEK293T cells were co-transfected with a lentiviral plasmid containing each gene, psPAX2 (no. 1259, Addgene) and pMD2.G (no. 12260, Addgene) at a ratio of 1:0.75:0.25. After 72 h of transfection, cells were centrifuged at 1000*g* for 5 min and supernatant fractions containing lentiviruses were collected and filtered through a 0.45-μm sterile filter to remove cell debris. Cells were treated with viral supernatant and 8 μg/ml polybrene (Sigma-Aldrich, Burlington, MA, USA, catalog no. H9268), followed by incubation for 48 h. After viral transduction, Flag-tagged SETDB1, VHL or shRNA-expressing cells were subjected to the appropriate treatments and selected with 1 μg/ml puromycin or 5 μg/ml blasticidin. Cells expressing sgRNA were sorted based on RFP using a BD FACS Aria™ Fusion instrument (BD, Franklin Lakes, NJ, USA).

### Transient cell transfection

Transient expression of ectopic proteins and viral particles was achieved in 70–80% confluent cells by transfecting the indicated eukaryotic plasmid using X-tremeGENE™ HP DNA Transfection Reagent (catalog no. 6366244001, Roche, Basel, Switzerland). Cells were analyzed 48 h after transfection. Transient KDwas performed by transfection of siRNA with Lipofectamine RNAiMAX Reagent (catalog no. 13778100, Thermo Fisher) according to the manufacturer's protocol. siRNAs targeting the indicated genes were obtained from Bioneer. The target sequences are presented in Supplementary Table S1.

### Western blot and immunoprecipitation analyses

For analysis of protein levels in whole cell lysates, cells were lysed in RIPA (150 mM NaCl, 1.0% IGEPAL^®^ CA-630, 0.5% sodium deoxycholate, 0.1% sodium dodecyl sulfate (SDS), 50 mM Tris-HCl, pH 8.0) with an EDTA-free protease inhibitor cocktail tablet (catalog no. 04693132001, Roche) and sonicated using Bioruptor^TM^ (catalog no. UCD200, Diagnode, Denville, NJ, USA). Protein concentrations were quantified with the Bradford assay, diluted to the same concentration and boiled with SDS sample buffer. For immunoprecipitation experiments, cells were homogenized in lysis buffer (50 mM HEPES pH 7.5, 100 mM NaCl, 1% Triton™ X-100, 10 mM MgCl_2_) containing an EDTA-free protease inhibitor cocktail tablet (catalog no. 04693132001, Roche) and subjected to sonication. Cell lysates were pre-cleared with Dynabeads^TM^ Protein A (catalog no. 10002D Invitrogen, Waltham, MA, USA) or Dynabeads^TM^ Protein G (catalog no. 10004D, Invitrogen) and incubated with Anti-FLAG® M2 Magnetic Beads (catalog no. M8823, Sigma-Aldrich), Pierce™ High Capacity Streptavidin Agarose (catalog no. 20357, Thermo Fisher), Pierce™ Anti-HA Magnetic Beads (catalog no. 88836, Thermo Fisher) or Dynabeads™ Protein A/G with the indicated antibodies for 4 h at 4°C. For pull-down of polyubiquitinated proteins, cells treated with bortezomib (catalog no. S1013, Selleckchem) were lysed by incubating in Tris-EDTA buffer (50 mM Tris-HCl, pH 7.5, 0.15 M NaCl, 1 mM EDTA) containing 1% NP-40 and 10% glycerol. Poly-ubiquitinated proteins were pulled down using a Tandem Ubiquitin Binding Entities (TUBE) kit (catalog no. UM501M, LifeSensors) in the presence of 10 mM *N*-ethylmaleimide (NEM; catalog no. 04259, Sigma-Aldrich) according to the manufacturer's protocol.

Next, washed beads were boiled with SDS sample buffer. Prepared cell lysate samples were separated via SDS–PAGE and transferred to NC membrane (catalog no. 10600096, GE healthcare, Chicago, IL, USA). Blots were probed with the indicated primary antibodies, followed by anti-rabbit IgG. HRP-linked Antibody Detection was performed with Pierce™ ECL Plus Western Blot Substrate (catalog no. 32132, Thermo Fisher) or SuperSignal™ West Femto Maximum Sensitivity Substrate (34094, Thermo Fisher). The antibodies used for western blots are listed in Supplementary Table S1.

### Subcellular fractionation

Subcellular fractionation was conducted using hypotonic buffer. Cells (5 × 10^6^) were washed twice in PBS and once in Buffer A (10 mM HEPES, 1.5 mM MgCl_2_, 10 mM KCl, 0.5 mM DTT and an EDTA-free protease inhibitor cocktail tablet (catalog no. 04693132001, Roche)). Next, cells were resuspended in Buffer A plus 0.1% (v/v) IGEPAL and incubated on ice for 10 min. Following centrifugation at 1400*g* for 4 min at 4°C, the supernatant containing the cytosolic fraction was collected. To isolate the total nuclear fraction, the nuclear pellet was lysed in 1% SDS plus 1:100 benzonase (E1014, Sigma Aldrich) for 20 min at room temperature. For further separation into nucleosolic and chromatin fractions, the nuclear pellet was resuspended in Buffer B (20 mM HEPES, 1.5 mM MgCl_2_, 300 mM NaCl, 0.5 mM DTT, 25% v/v glycerol, 0.2 mM EDTA and an EDTA-free protease inhibitor cocktail tablet) for 10 min on ice. Following centrifugation at 1700*g* for 4 min at 4°C, the supernatant containing the nucleosolic fraction and insoluble pellet (chromatin fraction) were separated. The pellet was solubilized using 1% SDS plus 1:100 benzonase.

### Immunofluorescence analysis

Cells were seeded on μ-Slide 8 wells (catalog no. 80826, Ibidi, Fitchburg, WI, USA) at a density of 2 × 10^4^ cells per well. Cells were fixed with 4% paraformaldehyde for 20 min, permeabilized with PBS plus 0.1% Triton X-100 for 5 min, blocked with 4% BSA in PBS for 30 min at room temperature and incubated with the indicated primary antibodies for 6 h at 37°C. For staining the R-loop, cells were fixed with ice-cold 100% methanol at −20°C for 15 min, permeabilized as before, blocked with 1 mg/ml BSA in PBS for 30 min and incubated with the appropriate primary antibody. Cells were subsequently washed with 0.01% Tween-20 in PBS (PBST) and incubated with the appropriate secondary antibodies for 1 h at room temperature. Each well was washed with PBST, mounted in DAPI using Mounting Medium with DAPI-Aqueous, Fluoroshield (catalog no. ab104139, Abcam, Cambridge, UK). For EdU incorporation analysis, cells were stained using a Click-iT™ EdU Cell Proliferation Kit for Imaging (catalog no. C10337, Thermo Fisher) according to the manufacturer's protocol and imaged under a Zeiss LSM 880 confocal laser microscope (Zeiss, Oberkochen, Germany). The antibodies used for immunofluorescence analysis are listed in Supplementary Table S1.

The proximity ligation assay (PLA) was conducted using Duolink® PLA reagents (catalog no. DUO92101, Sigma-Aldrich) in keeping with the manufacturer's protocol. Cells were imaged using a Zeiss LSM 880 confocal laser microscope (Zeiss). The antibodies used for PLA are listed in Supplementary Table S1.

### *In vitro* assay

The activity of the 3PA SETDB1 mutant was measured with an *in vitro* histone methylation assay using Flag-tagged SETDB1 purified from a lentiviral Flag-SETDB1-expressing stable cell line. Within a reaction volume of 20 μl, 1 μg SETDB1 and 1.5 μg core histones were incubated at 37°C for 2 h in 50 mM pH 8.5 Tris-HCl, 20 mM KCl, 10 mM MgCl_2_, 1 mM β-mercaptoethanol and 0.01% Tween-20. Reactions were terminated by the addition of 5× SDS-buffer and protein levels analyzed via western blot. The activity of SETDB1 following dimethyloxaloylglycine (DMOG) treatment was measured by *in vitro* histone methylation assay using immunoprecipitated Flag-tagged SETDB1, overexpressed in HEK293T cells. Bead-bound, immunoprecipitated SETDB1 and 1.5 μg core histones were incubated with 50 mM pH 8.5 Tris-HCl, 20 mM KCl, 10 mM MgCl_2_, 1 mM β-mercaptoethanol and 0.01% Tween-20 (total volume, 20 μl) at 37°C for 2 h. Reactions were terminated by the addition of 5× SDS buffer, after which protein levels analyzed by western blot analysis.

For the *in vitro* ubiquitination assay, immunoprecipitated wild-type (WT) or 3PA SETDB1 protein was incubated with His-tagged ubiquitin (5 μM, His-Ub), CRL2^VHL^ complex (1 μg; catalog no. 23–044, Merck, Kenilworth, NJ, USA), UBE1 (20 nM; catalog no. 23–021, Merck) and UbcH4 (2 μM; catalog no. 23–025, Merck) in reaction buffer (25 mM MOPS pH 7.5, 0.01% Tween-20, 5 mM MgCl_2_, 10 μM ATP) at 37°C for 2 h. His-Ub was purified as described previously (21). Reactions were terminated by the addition of 5× SDS buffer and analyzed by western blotting.

For the *in vitro* hydroxylation assay, immnoprecipitated Flag-tagged SETDB1 or HA-tagged HIF1α was incubated with 500 nM of recombinant PHD1 (catalog no. 81064, Active motif, Tegernheim, Bayern, Germany) and PHD3 (catalog no. 81033, Active motif) in the reaction buffer (40 mM Tris-HCl pH 7.5, 10 mM KCl, 30 μM MgCl_2_, 2 mM DTT, 200 μM 2-oxoglutarate, 2 mM ascorbate, 100 μM (NH_4_)2Fe(SO_4_)2·6H_2_O) for 6 hours at 37ºC. Reactions were terminated by the addition of 5× SDS-buffer and analyzed by western blotting.

### Mass spectrometry analysis

For analysis of the interactome, eluted proteins from immunoprecipitation experiments were digested using Strap micro (C02-micro-80, Protifi, Melville, NY, USA) following the manufacturer's protocol (v.4.7). Briefly, 1 μg trypsin/lysC (V5073, Promega) was added to each sample and incubated overnight at 37°C. Cleaved peptides were desalted with a C18 spin tip (84850, Thermo Fisher) and dried under a speed vac. The RSLCnano u3000/Orbitrap Exploris 240 system (Thermo Fisher) was used for analysis. Each sample was dissolved in 0.1% FA/2% acetonitrile and peptides from 0.5–1 μg protein loaded onto the trap column (Acclaim PepMap 100, 75 μm × 2cm, C18, 3 μm, PN 164535). Peptides were separated using an analytical column (PepMap RSLC C18, 75 μm × 25 cm, 2 μm, 100 Å, PN ES802) at a temperature of 50°C. The mobile phases comprised 0.1% formic acid in water (buffer A) and 0.1% formic acid in acetonitrile (buffer B). The following gradient was used at a flow rate of 300 nl/min: 2% B to 20% B in 100 min and 20% B to 32% B in 20 min. The survey scan settings were as follows: resolution = 120 000, maximum IT = 100 ms, AGC 300%, mass range 375–1200 Th. The selected precursor was fragmented via high-collision dissociation and analyzed using Orbitrap. Other parameters for the MS/MS scan were as follows: Top15 double play, Resolution = 15 000, maximum IT = 22 ms, AGC standard, Threshold 5E3, normalized collision energy = 30%, isolation width = 1.4, dynamic exclusion parameter exclusion after *n* times = 1, exclusion duration time = 10 s, mass tolerance low/high = 10 ppm. Raw data from LC–MS were analyzed with MaxQuant v.1.6.10.43 and Perseus v.1.5.8. MaxQuant parameters were as follows: database = UniProt homo sapiens, enzyme = trypsin/P, variable modification = oxidation (M), acetyl (protein N-terminal), fixed modification = methylthio(C), LFQ and match between runs. The ProteinGroup.txt file was filtered for ‘razor + unique peptide ≥ Perseus’ and ‘LFQ intensity’ of each sample compared.

For post-translational modification (PTM) analysis of SETDB1, Flag-tagged SETDB1 was overexpressed in HEK293T cells, followed by immunoprecipitation and protein separation via SDS–PAGE. Immunoprecipitated SETDB1 was digested using S-Trap micro columns (C02-micro-80; Protifi, Melville, NY, USA) following the manufacturer's protocol (v.4.7). Briefly, 1 μg trypsin/lysC (catalog no. V5073, Promega) was added to each sample with overnight incubation at 37°C. Cleaved peptides were desalted with a C18 spin tip (84850, Thermo Fisher) and dried under a speed vacuum. The RSLCnano u3000/Orbitrap Exploris 240 system (Thermo Fisher) with FAIMS was used for analysis. Each sample was dissolved in 0.1% FA/2% acetonitrile and peptides from 0.5–1 μg protein loaded onto the trap column (Acclaim PepMap 100, 75 μm × 2 cm, C18, 3 μm, PN 164535). Peptides were separated using an analytical column (BEH300 C18, 75 μm × 25 cm, 1.7 μm, PN 186007484) at a temperature of 50°C. The mobile phases were 0.1% formic acid in water (buffer A) and 0.1% formic acid in acetonitrile (buffer B). The following gradient was used at a flow rate of 300 nl/min: 5%B to 7%B in 3 min, 7%B to 20%B in 73 min, 20%B to 28%B in 36 min, 28% B to 60% B in 8 min.

The survey scan settings were as follows: resolution = 60 000, maximum IT = 100 ms, AGC 300%, mass range 350–1200 Th. The selected precursor was fragmented via high-collision dissociation and analyzed using Orbitrap. FAIMS CV −45, −60 was used for analysis with cycle times of 1.7 and 1.3 s, respectively. Other parameters for the MS/MS scan were as follows: resolution = 30 000, maximum IT = 200 ms, AGC standard, threshold 1E5, normalized collision energy = 30%, isolation width = 2.0, dynamic exclusion parameter (exclude after *n* times = 1), exclusion duration time = 20 s, mass tolerance low/high = 10 ppm.

Raw data from LC–MS were analyzed with MaxQuant v.2.1.0.0 using the following parameters: database = UniProt *Homo sapiens*, enzyme = trypsin/P, variable modification = oxidation (M), oxidation (P), acetyl (protein N-terminal), fixed modification = methylthio (C) and match between runs. Intensity values of proteins and peptides from MaxQuant results were used for calculation of the modification rate.

### Colony formation assay

For analysis of colony formation, cells were split, seeded (400 cells/well) in six-well plates and cultured at 37°C for 3 weeks, followed by staining with 0.05% crystal violet. Images of the plates were scanned using ImageScanner III (GE Healthcare, Chicago, IL, USA).

### Caspase 3/7 assay

For analysis of caspase 3/7 activity, cells were seeded into white-walled 96-well plates to a final cell density of 3 × 10^3^ cells/well and pretreated with or without 2,2 bipyridyl (200 μM) for the indicated times. The activity levels of caspase 3/7 were analyzed with the Caspase-Glo 3/7 assay (catalog no. G8092, Promega, Madison, WI, USA) according to the manufacturer's instructions. Briefly, plates were equilibrated to room temperature and Caspase-Glo® 3/7 reagent (100 μl) added into each well. Following incubation at room temperature for 30 min, luminescence signals were detected with Victor X3 (Perkin Elmer, Waltham, MA, USA).

### RNA isolation and quantitative RT–PCR

Total RNA was extracted using two different protocols, specifically, Qiagen RNeasy kit (catalog no. 74104, Qiagen, Germantown, MD, USA) for conventional mRNA and TRIzol® for analyzing repetitive elements, in keeping with the manufacturers' recommendations. cDNA was synthesized with oligo dT for mRNA and random primer for total RNA using the RevertAid First Strand cDNA Synthesis Kit (K1622, Thermo Fisher). Quantitative PCR (qPCR) was conducted using a Solg™ Real-Time PCR Kit with EvaGreen™ intercalating dye detection (catalog no. SRH91-R25h, Solgent, Daejeon, South Korea). Actin was employed as the internal control. The primers used are listed in Supplementary Table S1.

### Chromatin immunoprecipitation and qPCR

Cells were cross-linked by adding 1% formaldehyde to the medium for 15 min at room temperature. After quenching with 0.125 M glycine, fixed cells were washed twice with 1 × PBS, pelleted by centrifugation at 1000*g* for 5 min and lysed in lysis buffer (1% SDS, 10 mM EDTA, 50 mM Tris-HCl, pH 8.1 and EDTA-free protease inhibitors) for 1 h at 4°C. Cells were subsequently sonicated using the Covaris system (shearing time 5 min, 20% duty cycle, intensity of 10 200 cycles per burst and 30 s per cycle) in a total volume of 1 ml. Dynabeads^TM^ Protein A (Invitrogen, catalog no. 10002D), Dynabeads^TM^ Protein G (Invitrogen, catalog no. 10004D) and 10 μg antibody against SETDB1 or H3K9me3 were bound for 3–4 h at 4°C in PBS, added to chromatin and incubated overnight at 4°C. Immunoprecipitated chromatin was washed three times with wash buffer 1 (50 mM HEPES pH 7.5, 500 mM NaCl, 1 mM EDTA, 1% Triton X-100, 0.1% sodium deoxycholate, 0.1% SDS) and twice with wash buffer 2 (10 mM Tris pH 8, 0.25 M LiCl, 0.5% NP-40, 0.5% sodium deoxycholate, 1 mM EDTA, 1 mM PMSF). Bound DNA was eluted from beads with 200 μl of elution buffer (1% SDS, 10 mM EDTA and 50 mM Tris-HCl, pH 8.1) and incubated for 45 min at 65°C. For reverse cross-linking, the supernatant was incubated for 4 h at 65°C and treated with 7.5 μl proteinase K (20 mg/ml, 70663, Merck) and 1 μl of RNase A (RNASEA-RO, Roche) for 1 h at 37°C. DNA was purified using phenol-chloroform-isoamyl alcohol. Immunoprecipitated DNA was used to perform qPCR with EvaGreen™ intercalating dye detection. The relative amount of each amplified fragment was normalized to that of input DNA. Primers were designed using the NCBI Primer-BLAST. Primers for repetitive element PCR were designed based on Dfam (22) consensus sequence and RepeatMasker (23). The primers used are listed in Supplementary Table S1.

### RNA sequencing analysis

The RNA sequencing (RNA-seq) library for transcriptomic analysis was prepared using the TruSeq Stranded mRNA Sample Prep Kit (catalog no. 20020595, Illumina, San Diego, CA, USA). For analysis of repetitive elements, a library was prepared using the TruSeq stranded total RNA Sample prep Kit (20020597, Illumina). Sequencing was performed using the Illumina HiSeq2000 platform to generate 100 bp paired-end reads. The human reference genome was obtained from the NCBI genome (https://www.ncbi.nlm.nih.gov/genome/) and genome indexing performed using STAR (v.2.5.1) (24). Sequenced reads were mapped to human genome (hg19) STAR and gene expression levels quantified with the count module. The edgeR (v.3.12.1) (25) package was applied to select differentially expressed genes from RNA-seq counts between samples (fold change > 1.5, *P* value < 0.05). The trimmed mean of M-value normalization-normalized counts per million value of each gene was added to 1 and log_2_-transformed for further analysis. A heatmap was generated using MeV (26) and the R (v.3.5.0, https://www.r-project.org/) pheatmap package (v.1.0.12, https://cran.r-project.org/web/packages/pheatmap/index.html).

For quantification of repetitive elements in bulk RNA-seq data, the RepEnrich (27) (v.1.2) tool was used as a default option. After downloading the hg19 version of repetitive element annotation provided by the tool, the genome was constructed using the ‘RepEnrich_setup.py’ module. RNA-seq data were mapped to the genome using Bowtie1 (v.1.2.3) (28) to quantify the read count of each repetitive element and expression summarized as counts per million values. Using the edgeR package, differentially expressed repetitive elements for each condition were summarized (fold change > 1.5, *P* value < 0.05). NGS data were deposited in the NCBI Gene Expression Omnibus under accession number GSE179331. Raw sequence tags were deposited in the NCBI Short Read Archive under accession numbers SRP326658 and SRP326663.

### Gene set enrichment analysis

For gene set enrichment analysis (GSEA) analysis of RNA-seq data, we used GSEA software (29) from the Broad Institute website (v.4.0.0, https://www.gsea-msigdb.org/gsea/index.jsp). Our expression dataset was analyzed against the hallmark, KEGG pathway and GO gene sets (H, C2 and C5.gmt files from MSigDB v.7.0). The statistical significance (nominal *P* value) of the enrichment score (ES) was calculated by running 500 gene set permutations. The normalized enrichment score accounted for the size of the gene set.

### Other reagents

DMOG (catalog no. S7483, Selleckchem), 2,2′-bipyridyl (catalog no. D216305, Sigma-Aldrich), roxadustat (catalog no. FG-4592, Selleckchem), cycloheximide (catalog no. C1988, Sigma-Aldrich), nocodazole (catalog no. M1404. Sigma-Aldrich) were used as indicated in the text.

### Transcriptomic profiling of patient data from the Pan-Cancer Atlas (PanCanAtlas)

To perform gene expression profiling of patients, we obtained a transcriptome dataset (32 cancer types, *n* = 10 117) from the PanCanAtlas consortium (https://gdc.cancer.gov/about-data/publications/pancanatlas) (30). For determining the hypoxia status of each patient, a signature-based hypoxia scoring method using *Buffa* signature was adopted (31–33). To determine differentially expressed genes between two subgroups, a multiple testing procedure involving the two sample *t*-test with 1000 permutations was applied to compute permutation adjusted *P* values (R package: *multtest*). Functional enrichment analysis was conducted to identify the most significant biological processes associated with each molecular subgroup using DAVID bioinformatic resources (v.6.8, https://david.ncifcrf.gov). GSEA was performed using the GSEA tool (v.4.1.0).

### Statistical analysis

One-way ANOVA followed by Dunnett's multiple comparison test, two-way ANOVA followed by Bonferroni multiple comparison test and cumulative frequency distribution followed by Kolmogorov–Smirnov test were applied for statistical analyses using GraphPad Prism5 and Prism9 software (GraphPad, San Diego, CA, USA). Quantified data were assessed using independent Student's *t*-test (*P < 0.05, **P < 0.005, ***P < 0.0001).

## Results

### VHL interacts with SETDB1 and regulates its stability

**Figure 1.**
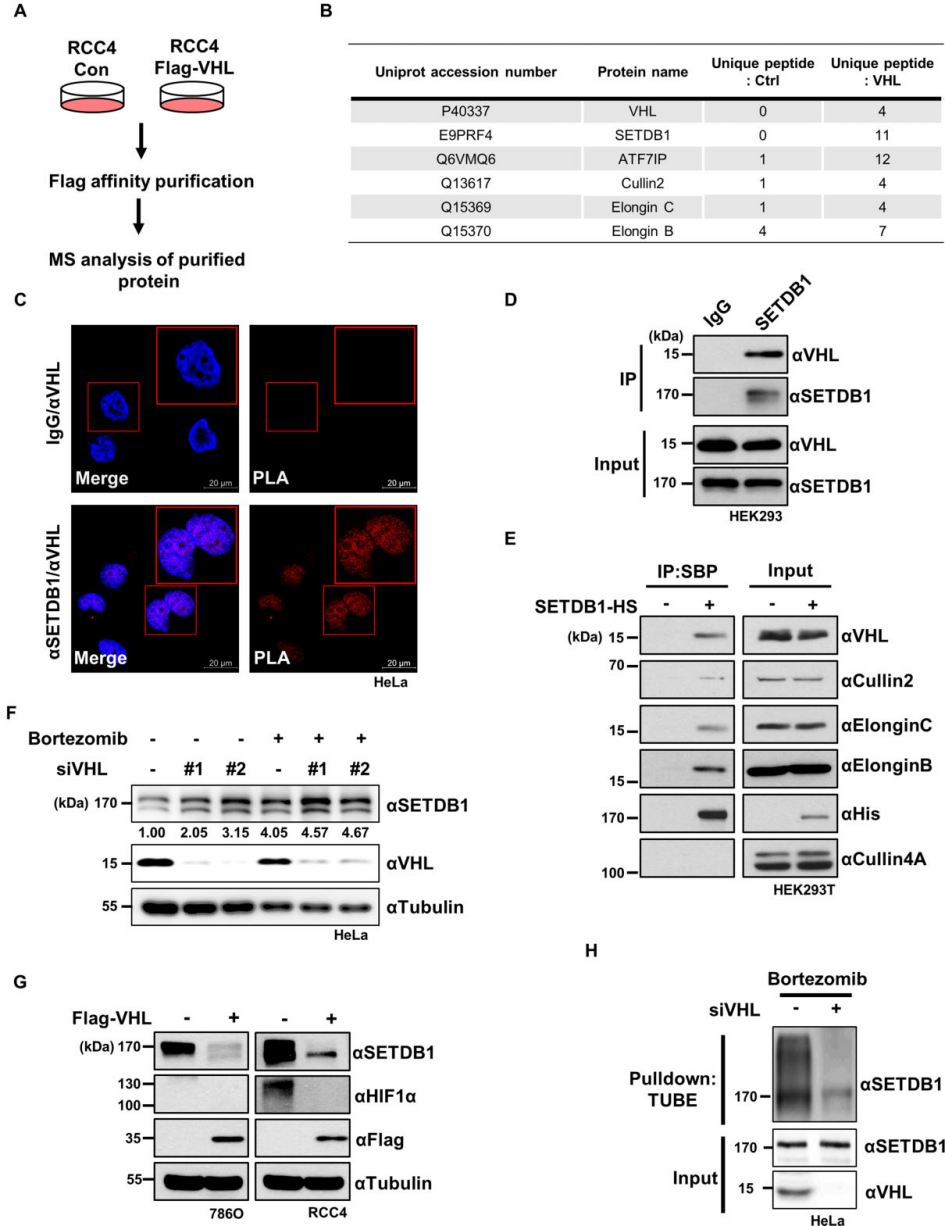
VHL mediates ubiquitin-dependent degradation of SETDB1. **(A)** Schematic workflow of MS analysis. Whole cell lysates from RCC4 cells stably expressing Flag- tagged VHL (Flag-VHL) and control (Con) were immunoprecipitated using anti-Flag antibodies. Immunoprecipitated proteins were analyzed using mass spectrometry. **(B)** MS analysis of VHL-associated proteins. **(C)** PLA using either anti-SETDB1/anti-VHL antibodies or anti-IgG/anti-VHL to determine the association of VHL and SETDB1 in HeLa cells. Representative images of PLA (red) and those merged with DAPI staining (blue) are shown. Scale bars denote 20 μm. **(D)** Examination of the physical association of endogenous SETDB1 with VHL via immunoprecipitation (IP) against SETDB1 or IgG followed by immunoblotting with the indicated antibodies. **(E)** Cellular interactions between ectopically expressed SETDB1 tagged with histidine and streptavidin-binding protein (SBP) and CRL2^VHL^ in HEK293T analyzed via immunoprecipitation (IP) against the SBP epitope followed by immunoblotting with the indicated antibodies. **(F)** Immunoblot analysis with antibodies against SETDB1 and VHL to determine the effects of VHL KDwith siRNAs (siVHL) in HeLa cells treated with bortezomib (1 μM) for 12 h. **(G)** Effect of Flag-tagged VHL overexpression (Flag-VHL) on abundance of endogenous SETDB1 and HIF1α in 786O and RCC4 cells assessed by immunoblotting with the indicated antibodies. **(H)** Downregulation of endogenous SETDB1 ubiquitination by siRNA-mediated KDof VHL (siVHL) in HeLa cells treated with bortezomib (1 μM) for 12 h. Non-targeting siRNA was used as a negative control.

To uncover potential novel substrate targets of CRL2^VHL^ other than well-known HIF1/2α , we analyzed the physical interactome of VHL. To this end, RCC4 cells stably expressing Flag-tagged VHL were established (Figure 1A). Immunoprecipitation with anti-FLAG antibody, followed by mass spectrometry analysis, revealed that VHL associates with the histone H3K9 methyltransferase SETDB1 and its binding partner ATF7IP, along with subunits of the CRL2^VHL^ complex, such as Cullin2 and Elongin B/C (Figure 1B). Proximity ligation analysis (PLA) additionally showed that SETDB1 and VHL interacted with each other mainly in the nucleus of HeLa cells whereas negative controls showed no such interaction (Figure 1C). In co-immunoprecipitation experiments, SETDB1 interacted with endogenous VHL as well as other CRL2^VHL^ components (Figure 1D, E), supporting the possibility that the CRL2^VHL^ complex ubiquitinates and degrades SETDB1. A subsequent investigation of the effect of VHL on the abundance of SETDB1 showed that either knockdown (KD) of VHL or treatment with the proteasome inhibitor, bortezomib, resulted in upregulation of SETDB1, without an additive effect of KD and bortezomib (Figure 1F). On the other hand, overexpression of VHL induced downregulation of SETDB1 in VHL-deficient renal carcinoma cells (Figure 1G). In addition, KD of VHL in HEK293T cells increased the half-life of SETDB1 to about twice as long as that in control cells (Supplementary Figure S1A, B). Ubiquitination assays showed poly-ubiquitination of SETDB1 in cells, an effect that was decreased on VHL KD (Figure 1H, Supplementary Figure S1C, D). Conversely, VHL overexpression led to increased poly-ubiquitination of SETDB1 (Supplementary Figure S1E). Taken together, these results demonstrate that VHL regulates SETDB1 protein stability via the ubiquitin-proteasome pathway.

### Inactivation of PHDs in hypoxia stabilizes SETDB1 in a HIF-independent manner

**Figure 2.**
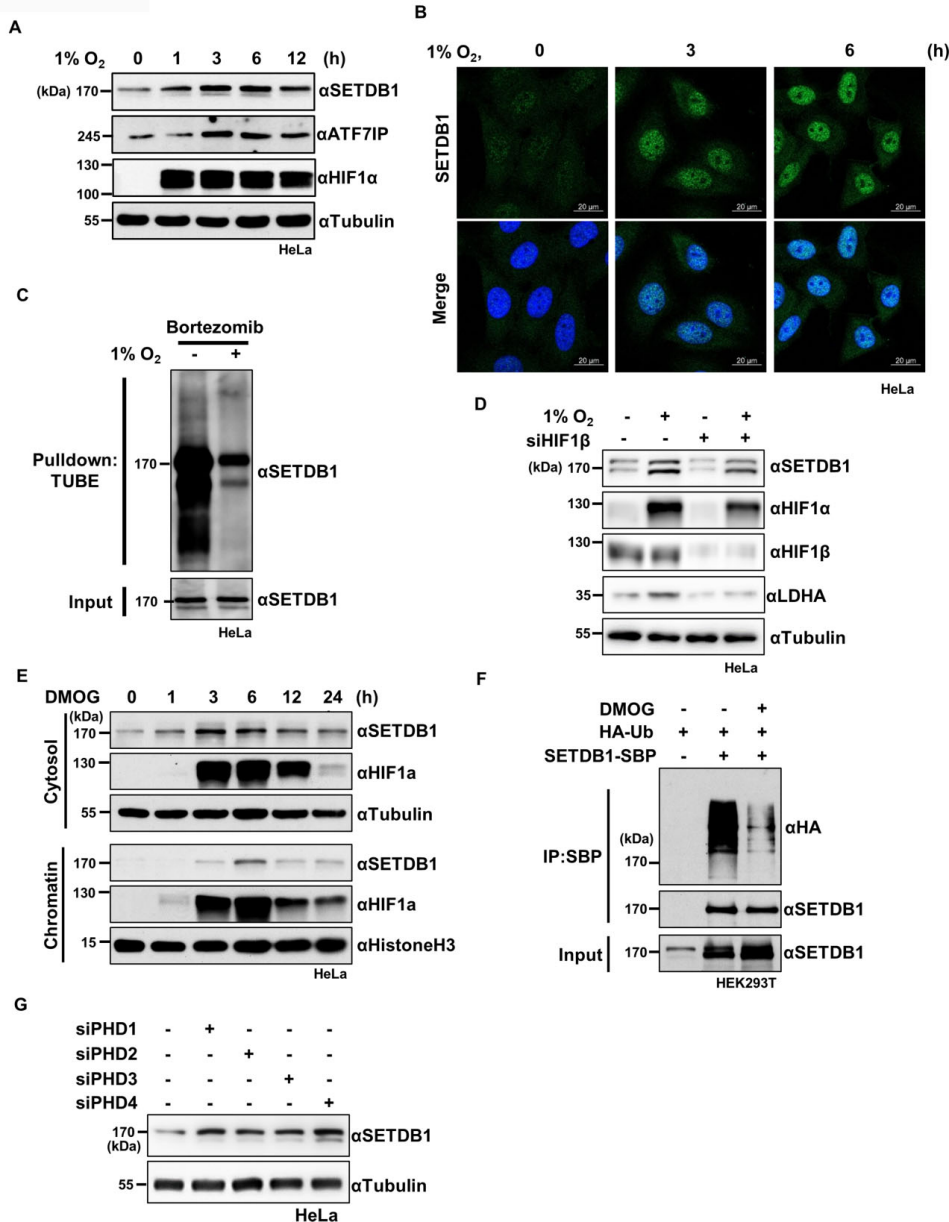
Hypoxia stabilizes SETDB1 through inactivating PHDs. **(A)** Immunoblot analysis showing time-dependent changes in SETDB1 and ATP7IP levels in HeLa cells exposed to 1% O_2_. **(B)** Increased SETDB1 levels in HeLa cells exposed to 1% O_2_ for the indicated times, analyzed by immunostaining with anti-SETDB1 antibody and DAPI. Scale bar denotes 20 μm. **(C)** Effect of hypoxia (1% O_2_, 12 h) on polyubiquitination of endogenous SETDB1 in HeLa cells treated with bortezomib (1 μM, 12 h), analyzed by ubiquitination assay using TUBE. **(D)** Effect of siRNA-mediated HIF1β depletion (siHIF1β) on the increase in SETDB1 levels in HeLa cells exposed to 1% O_2_ for 6 h, examined by immunoblotting with the indicated antibodies. Non-targeting siRNA was used as a negative control **(E)** Effect of a pan-PHD inhibitor, DMOG, on SETDB1 levels in the cytosolic and chromatin fractions of Hela cells evaluated by immunoblotting with the indicated antibodies. **(F)** Effect of DMOG (1 mM, 6 h) on polyubiquitination of ectopic SETDB1 tagged with SBP (SETDB1-SBP) in HEK293T cells, analyzed by immunoblotting with the indicated antibodies. **(G)** Effect of siRNA-mediated KD of individual PHD isoforms (PHD1-4) on SETDB1 levels in HeLa cells, determined by immunoblotting using the indicated antibodies Non-targeting siRNA was used as a negative control.

Given that VHL regulates the stability of its substrates, such as HIF1/2α, in an oxygen-dependent manner, we examined the effect of hypoxic stress on SETDB1 levels. Our results showed an inverse correlation of SETDB1 protein expression with oxygen availability (Supplementary Figure S2A). Notably, while HIF1α was rapidly stabilized (<1 h) in hypoxia, the highest increase in SETDB1 protein expression was detected 3 h after induction of hypoxic stress (Figure 2A). Consistent with this finding, immunostaining analyses revealed increased SETDB1 protein levels in hypoxia (Figure 2B, Supplementary Figure S2B). However, mRNA levels of *SETDB1* remained unchanged under hypoxic conditions (Supplementary Figure S2C). The ubiquitination assay further verified that hypoxia suppressed poly-ubiquitination of SETDB1 (Figure 2C). In keeping with hypoxia-induced SETDB1 stabilization, treatment with the hypoxia mimetic compound, 2,2′-bipyridyl, induced upregulation of SETDB1 in a time-dependent manner (Supplementary Figure S2D). In addition, re-oxygenation following hypoxic stress compromised the increase in SETDB1 (Supplementary Figure S2E). SETDB1 forms a heterodimeric complex with ATF7IP and the stability of each protein is regulated by its binding partner (34). We observed an increase in ATF7IP concordant with SETDB1 elevation in hypoxia (Figure 2A). However, ATF7IP KD failed to affect the increase of SETDB1 under hypoxia (Supplementary Figure S2F), eliminating the possibility that hypoxia-induced SETDB1 stabilization is dependent on the ATF7IP level. Moreover, SETDB1 levels were elevated in various non-malignant and malignant cells under hypoxic stress or in response to 2,2′-bipyridyl treatment (Supplementary Figure S3A–F), but remained unchanged in VHL-deficient RCC4 cells (Supplementary Figure S3G).

To determine whether hypoxia-induced SETDB1 stabilization depends on HIFs, which are considered master regulators of the hypoxia response, we examined SETDB1 levels in aryl hydrocarbon receptor nuclear translocator (ARNT/HIF1β)-depleted cells deficient of HIF activity (35). Whereas ARNT/HIF-1β depletion impaired the hypoxia-induced increase in the level of LDHA, a known target of the HIF complex, its effect on SETDB1 protein levels was negligible under both normoxic and hypoxic conditions (Figure 2D). These results clearly indicate that hypoxia-induced SETDB1 stabilization is independent of HIF activity.

In view of the finding that VHL mediates degradation of target proteins via recognition of hydroxylated proline residues, we further investigated whether PHDs are involved in VHL-induced SETDB1 degradation. Treatment with 2-oxoglutarate dimethyloxaloylglycine (DMOG), a pan prolyl hydroxylase inhibitor, led to an increase in SETDB1 and HIF1α levels in the cytosol and at chromatin after 3 h (Figure 2E) and a decrease in poly-ubiquitinated SETDB1 (Figure 2F). Consistent with this, another well-known PHD inhibitor, roxadustat, like DMOG and hypoxic stress, also upregulated SETDB1 (Supplementary Figure S3H). By contrast, DMOG treatment had no effect on SETDB1 levels in VHL-deficient RCC4 cells (Supplementary Figure S3I). These results indicate that the activity of prolyl hydroxylases is required for the VHL-dependent regulation of SETDB1 stability. Because SETDB1 protein levels remained unchanged during cell-cycle progression, with or without DMOG treatment (Supplementary Figure S3J), we can rule out the possibility that increases in SEDB1 levels under prolyl hydroxylase-deficient conditions were related to the cell cycle. The involvement of PHDs 1–4, encoding different prolyl hydroxylase isoforms, in SETDB1 protein stability was further assessed by knocking down individual PHDs with specific siRNAs. Depletion of each PHD isoform alone increased SETDB1 levels (Figure 2G, Supplementary Figure S3K) to a similar degree. Moreover, simultaneous KD of different PHDs produced additive upregulation of SETDB1 (Supplementary Figure S3L). Collectively, these results imply that PHDs 1–4 are all involved in regulating the stability of SETDB1 and demonstrate that inactivation of PHDs under hypoxic conditions stabilizes SETDB1.

### Hydroxylation of SETDB1 at proline mediates VHL-induced degradation

**Figure 3.**
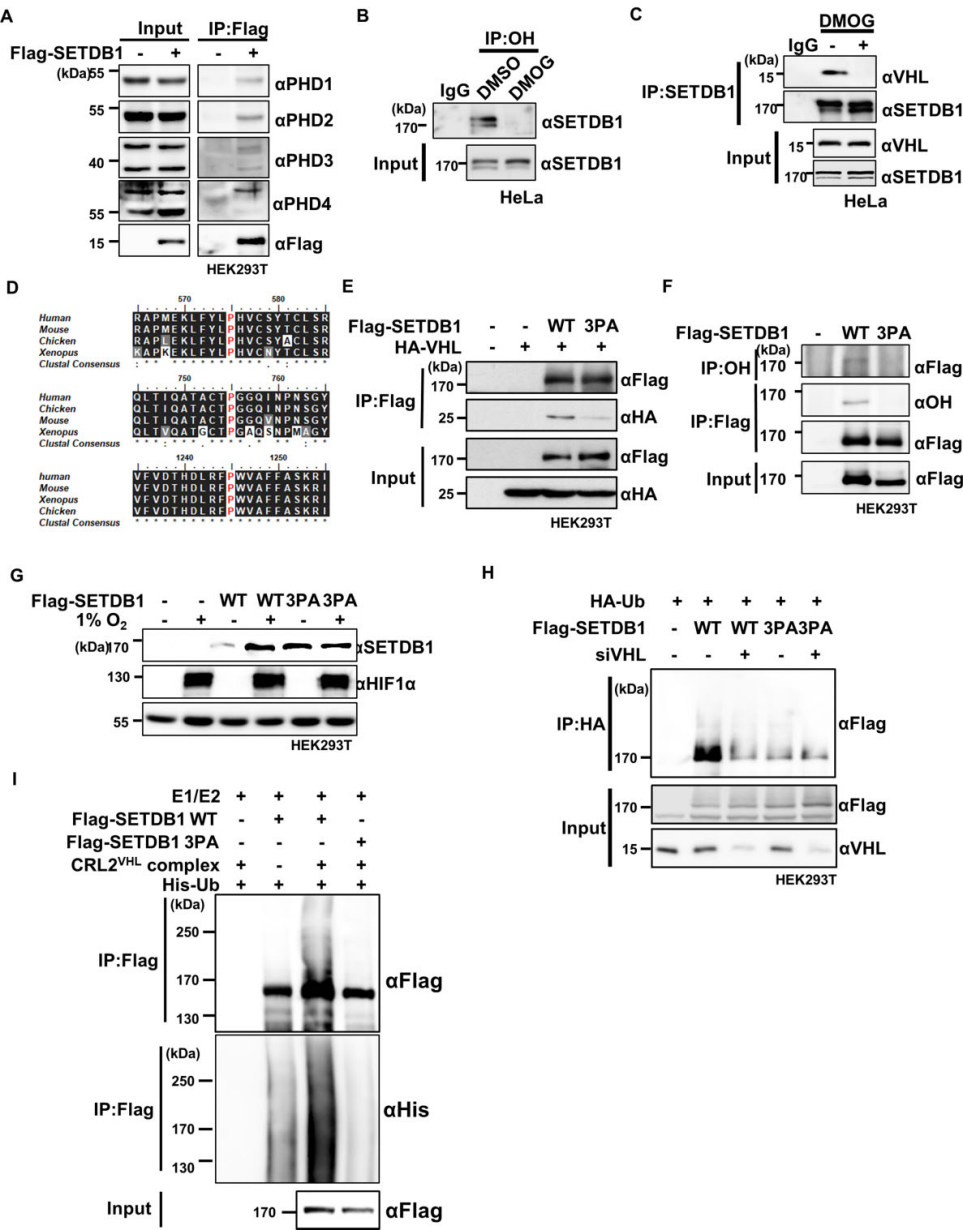
Hydroxylation of SETDB1 at proline residues promotes VHL-mediated degradation. **(A)** Binding of Flag-tagged SETDB1 to PHD1-4 proteins in HEK293T cells analyzed by immunoprecipitation (IP) with anti-Flag antibodies followed by immunoblotting with the indicated antibodies. **(B)** Endogenous hydroxylated proline-containing SETDB1 in HeLa cells, captured by immunoprecipitation with an anti-OH antibody followed by immunoblotting with a SETDB1 antibody, compared with that in DMOG-treated (1 mM, 24 h) HeLa cells. **(C)** Interaction of SETDB1 with VHL, with or without DMOG treatment (1 mM, 12 h), assessed by immunoprecipitation with the indicated antibodies. **(D)** Alignment of hydroxylated proline residues of SETDB1 homologs in different organisms. Conserved proline residues are marked with red. **(E)** Effects of simultaneous mutation of conserved proline to alanine (3PA) in SETDB1 on physical associations between Flag-tagged SETDB1 (Flag-SETDB1) and HA-tagged VHL (HA-VHL) in HEK293T cells, assessed by immunoprecipitation with anti-Flag followed by immunoblotting with the indicated antibodies. **(F)** Hydroxylated proline residues of Flag-SETDB1 wild-type (WT) and 3PA mutant SETDB1 in HEK293T cells were captured via immunoprecipitation with the indicated antibodies followed by immunoblotting. **(G)** Effect of SETDB1 3PA mutation on SETDB1 protein in HEK293T cells under normoxia or hypoxia (1% O_2_, 6 h) determined by immunoblotting with the indicated antibodies. **(H)** Effects of siRNA-mediated VHL depletion (siVHL) on polyubiquitination of Flag-tagged wild-type (WT) and 3PA mutant (3PA) SETDB1 in HEK293T cells determined with the ubiquitination assay as described in Supplementary Figure S1C. Non-targeting siRNA were used as a negative control. **(I)** Effect of 3PA mutation on polyubiquitination of Flag-tagged SETDB1 examined via an *in vitro* ubiquitination assay using recombinant histidine tagged-ubiquitin (His-Ub), E1, E2 and the CRL2^VHL^ complex, followed by immunoblotting with the indicated antibodies.

Next, we examined the possibility that PHDs directly hydroxylate proline residues of SETDB1. Data from co-immunoprecipitation assays showed that SETDB1 physically interacts with PHD1, PHD2, PHD3 and PHD4 (Figures 3A, Supplementary Figure S4A–D). However, *in vitro* hydroxylation assay of SETDB1 that was immunoprecipitated from HEK293T cells with recombinant PHD1 and PHD3 failed to generate hydroxylation on either SETDB1 or HIF1α, which was used as a positive control (Supplementary Figure S5A). Considering that a previous study has highlighted the challenges associated with reproducibility when attempting *in vitro* reconstitution of protein (36), we deopted to enhance the credibility of our findings regarding PHD-mediated SETDB1 hydroxylation in cells instead of persistently pursuing *in vitro* hydroxylation experiments. Moreover, proline hydroxylation on SETDB1 was detected with an antibody specific for hydroxyl proline residues (Figures 3B, Supplementary Figure S5B). The extent of SETDB1 proline hydroxylation was decreased by DMOG (1 mM), 2,2-bipyridyl (200 μM, 12 h) or hypoxia treatment (Supplementary Figure S5C). Given that VHL binds to hydroxyl proline residues of HIF1α and HIF2α (37,38), and that the interaction between SETDB1 and VHL was impaired by DMOG treatment (Figure 3C), we reasoned that proline hydroxylation on SETB1 is necessary for its physical interaction with VHL. To define the VHL-interacting region within SETDB1, we generated different truncated mutants of SETDB1 (T1–T6). Subsequent binding assays showed that the region spanning amino acids 556–1290 of SETDB1 is involved in binding to VHL (Supplementary Figure S5D-F). A mass spectrometry analysis of SETDB1 immunoprecipitated from HEK293T cells identified 23 putative hydroxylated proline residues, 13 of which are located within the VHL binding region (Supplementary Table S2). Of these 13 putative hydroxylated prolines, five residues on peptides detected with relatively high intensities were singled out for further investigation (Supplementary Figure S5G, H). Notably, DMOG treatment reduced the detection intensities of these peptides. To determine the involvement of proline residues in VHL binding, we substituted alanine for the residues that enable interaction with VHL in truncated forms of SETDB1 (T4-) (6). Subsequent assays of VHL binding to SETDB1 fragments harboring alanine substitutions at these residues revealed that P575, P755 and P1245 of SETDB1 are crucial for interactions of the truncated SETDB1 with VHL (Supplementary Figure S5I–K). These proline residues are evolutionarily well conserved across species from *Xenopus* to humans (Figure 3D). Notably, two of these residues (P755 and P1245) are located in pre-SET and SET domains essential for the methyltransferase activity of SETDB1. To examine the effect of these mutations on SETDB1–VHL binding, we mutated these proline residues in full-length SETDB1. Whereas single mutations of individual proline residues had little effect on physical interactions between SETDB1 and VHL (Supplementary Figure S5L), simultaneous triple mutation of all three proline residues (3PA) substantially weakened SETDB1 binding to VHL (Figure 3E). Notably, the 3PA mutations similarly decreased SETDB1 proline hydroxylation levels (Figure 3F). Moreover, the 3PA SETDB1 mutant protein was more abundant than wild-type SETDB1 in normoxia but not in hypoxia (Figure 3G). Consistent with this, the half-life of the 3PA SETDB1 mutant protein was ∼2-fold longer than that of wild-type SETDB1 (Supplementary Figure S5M, N). These results indicate that hydroxylated P575, P755 and P1245 residues cooperatively interact with VHL to destabilize SETDB1.

To ascertain whether these proline residues are necessary for ubiquitin-mediated proteasomal degradation, we examined poly-ubiquitination of 3PA SETDB1 in cells. The ubiquitination level of 3PA SETDB1 in cells with intact VHL was compromised to the extent of wild-type SETDB1 ubiquitination in VHL-depleted cells (Figure 3H). Moreover, VHL KD failed to further downregulate ubiquitination of 3PA SETDB1. An *in vitro* ubiquitination assay with immunoprecipitated SETDB1 and purified proteins disclosed that the CRL2^VHL^ complex efficiently ubiquitinated wild-type but not 3PA SETDB1 (Figure 3I). Additionally, to examine the effect of hydroxylation on the methyltransferase activity of SETDB1, we performed an *in vitro* methylation assay with SETDB1 immunoprecipitated from HEK293T cells treated with DMOG (1 mM) or DMSO control. We found no effect of DMOG treatment on the H3K9 methyltransferase activity of SETDB1 (Supplementary Figure S5O), implying that hydroxylation is not involved in the catalytic activity of SETDB1. However, 3PA mutant SETDB1 immunoprecipitated from HEK293T cells lacked methyltransferase activity (Supplementary Figure S5P). The fact that P755 and P1245 residues are located within pre-SET and SET domains, respectively, which are essential for methyltransferase activity, suggests that the PA substitutions induced a conformational change that inactivated the catalytic activity of SETDB1. Taken together, our findings indicate that the cooperative interaction of hydroxylated P575, P755 and P1245 residues on SETDB1 with VHL mediates ubiquitination-dependent proteasomal degradation of SETDB1.

### SETDB1 loss hyperactivates inflammatory responses related to innate immune signatures in hypoxia

**Figure 4.**
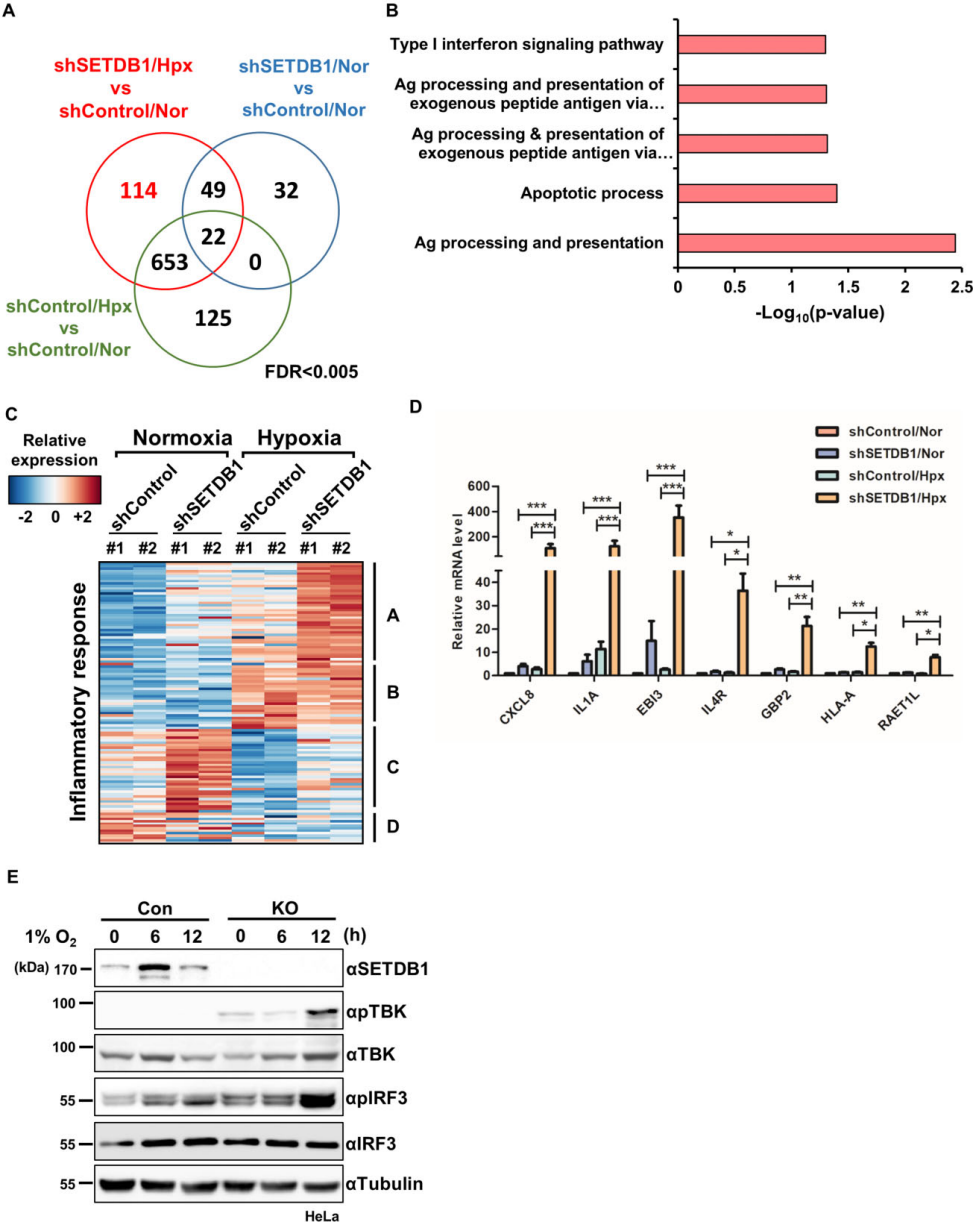
SETDB1 loss hyperactivates immune-inflammatory responses in hypoxia. **(A)** Venn diagram displaying differentially upregulated genes in HeLa cells depleted of SETDB1 with shRNA (shSETDB1) in normoxia (Nor) and hypoxia (Hpx, 1% O_2_, 12 h) compared with their counterparts in control cells (shCon) under normoxia. **(B)** Gene ontology (GO) analysis showing significant enrichment of innate immunity-related pathways of differentially upregulated genes specifically in shSETDB1/Hpx cells. **(C)** Heatmap displaying relative expression of genes corresponding to the inflammatory response pathway. **(D)** Combined effects of SETDB1 loss and hypoxia (1% O_2_, 12 h) on expression of genes linked to the inflammatory response and innate immune signature, determined by assessment of transcript levels with quantitative RT–PCR. Data are presented as means ± SEM of four independent experiments (*P < 0.05, **P < 0.005, ***P < 0.001, Student's *t*-test). **(E)** Effects of SETDB1 loss on activation of molecules in the inflammatory signaling pathway evaluated by immunoblotting of whole cell lysates of SETDB1 knockout (KO) or control (Con) HeLa cells under either normoxia or hypoxia (1% O_2_, indicated times) with the indicated antibodies.

To investigate the functional relevance of elevated SETDB1 in hypoxia, we analyzed the transcriptome of HeLa cells depleted of SETDB1 using specific shRNA (shSETDB1). Multidimensional scaling (MDS) plots demonstrated reproducibility of replicates (Supplementary Figure S6A). Differential analysis showed that 838 and 103 genes were upregulated in shSETDB1 cells under hypoxia (shSETDB1/Hpx) and normoxia (shSETDB1/Nor), respectively, compared with shControl cells under normoxia (shCon/Nor; FDR < 0.005; Figure 4A). In addition, 780 genes were upregulated in shControl cells under hypoxia (shCon/Hpx) relative to shCon/Nor. By eliminating overlapping genes among the different comparison sets, 114 genes were identified as differentially upregulated specifically in shSETDB1/Hpx group (Figure 4A). Gene ontology (GO) analysis of the differentially upregulated genes further revealed enrichment of innate immunity-related pathways in shSETDB1/Hpx (Figures 4B, Supplementary Figure S6B). GSEA of shSETDB1/Hpx and shSETDB/Nor was additionally performed to identify gene signatures altered by a combination of SETDB1 loss and oxygen depletion. To rule out the effects of hypoxia *per se* on gene expression, we excluded enriched signatures overlapping between ‘shSETB1/Hpx versus shSETDB1/Nor’ and ‘shCon/Hpx versus shCon/Nor’ groups, such as hypoxia and glycolysis. Accordingly, we observed enrichment of the inflammatory response signature in shSETDB1/Hpx (Supplementary Figure S6C), suggesting that the effect of SETDB1 loss on the inflammatory response is more potent in hypoxia than normoxia and inactivation of the protein in hypoxic conditions can trigger hyper-activation of innate immunity.

Comparative analysis of inflammatory response genes showed that genes upregulated in shSETDB1/Hpx included those displaying elevated expression in shSETDB1/Nor and shCon/Hpx, compared with shCon/Nor (Figure 4C, Groups A and B). While expression of Group B genes in shSETDB1/Hpx was comparable with those in shCon/Hpx, expression of group A genes was higher in shSETDB1/Hpx, implying that SETDB1 loss under hypoxia hyperactivates these genes. Intriguingly, the genes most highly expressed in shSETDB1/Nor were largely downregulated in shCon/Hpx compared with shCon/Nor (group C). Expression of group C genes in shSETDB1/Hpx and shCon/Nor groups was comparable, suggesting that SETDB1 depletion attenuates hypoxia-induced repression of group C genes. To validate differential gene expression in shSETDB1/Hpx, individual mRNA levels of seven representative genes (CXCL8, IL1A, EBI3, IL4R, GBP2, HLA-A and RAET1L) belonging to the inflammatory response pathway and innate immune signatures (Supplementary Figure S6D) were assessed. While expression of all the assessed genes was increased to some extent in both shSETDB1/Nor and shControl/Hpx compared with shControl/Nor, the fold increase in shSETDB1/Hpx was the highest with strong significance (Figure 4D).

TANK Binding Kinase 1 (TBK1) is a serine/threonine kinase with an important role in regulating inflammatory responses. Following activation by an inflammatory inducer, TBK1 is phosphorylated and activates IRF3/7 for type I interferon (IFN) production (39). To examine the functional association of SETDB1 with inflammatory signaling activity, we evaluated the phosphorylation status of TBK and IRF3 in HeLa cells with knockout (KO) of SETDB1. Consistent with transcriptomic analyses, while SETDB1 loss induced phosphorylation of TBK1 and IRF3 in normoxia to some extent, phosphorylated forms of TBK1 and IRF3 were the most significantly elevated in SETDB1 KO cells at 12 h under hypoxia (Figure 4E). Based on the collective results, we propose that SETDB1 loss hyperactivates an inflammatory response related to innate immunity under conditions of limited oxygen.

### Maintenance of the repressive state of TE elements in hypoxia requires increased chromatin occupancy of SETDB1

**Figure 5.**
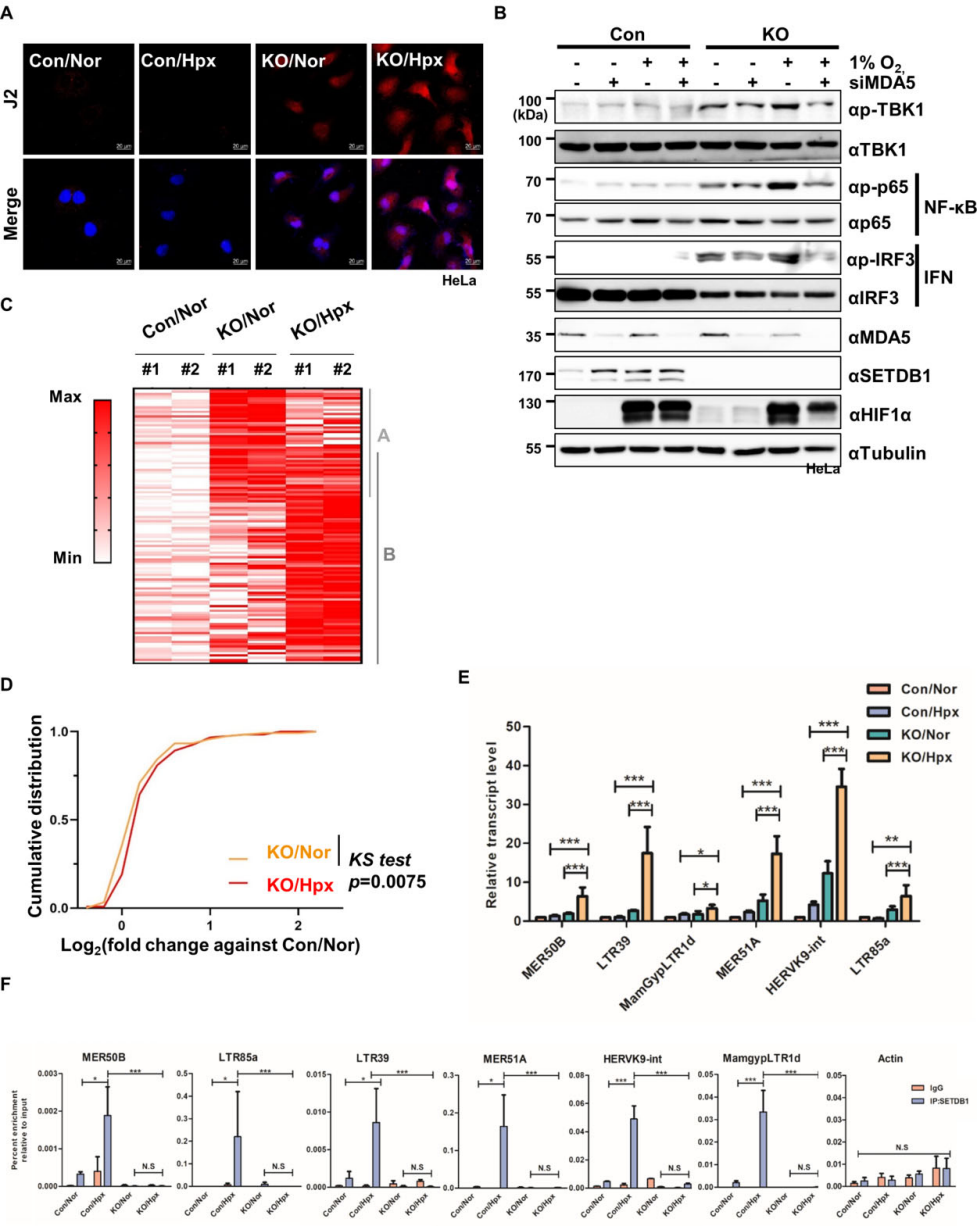
Maintenance of the repressive state of TE elements in hypoxia requires increased chromatin occupancy of SETDB1. **(A)** Representative images of dsRNA stained with J2 antibody (red) and DAPI (blue) in HeLa cells lacking SETDB1 (KO) compared with control cells (Con) in normoxia (Nor) and hypoxia (Hpx). Scale bar denotes 20 μM. **(B)** Effect of MDA5 depletion (siMDA5) on NF-κB and IFN-related inflammatory signaling activities determined by immunoblotting of SETDB1 KO (KO) or control (Con) HeLa cells exposed to hypoxic stress (1% O_2_, 12 h) with the indicated antibodies. **(C)** Heatmap showing relative expression of TEs differentially upregulated in SETDB1 KO Hela cells under normoxia (KO/Nor, A) and hypoxia (1% O_2_, 12 h; KO/Hpx, B) compared with control HeLa cells under normoxia (Con/Nor). **(D)** Cumulative distribution of fold changes in transcript abundance for TE groups A and B in SETDB1 KO cells under normoxia (KO/Nor) and hypoxia (1% O_2_, 12 h; KO/Hpx) relative to those in control cells under normoxia (Con/Nor). The *P* value was calculated with the Kolmogorov–Smirnov (KS) test. **(E)** Quantitative RT–PCR analysis of expression of individual TEs in SETDB1 KO (KO) and control (Con) HeLa cells under normoxia and hypoxia (1% O_2_, 6 h). Data are presented as means ± SEM of four independent experiments (**P < 0.005, ***P < 0.001, Student's *t*-test). **(F)** ChIP-qPCR analysis of SETDB1 enrichment at the indicated TE and Actin loci in SETDB1 KO (KO) and control (Con) HeLa cells under either normoxia or hypoxia (1% O_2_, 6 h). Results are expressed as fold enrichment relative to input DNA and presented as mean ± SEM (*n* = 3, **P < 0.005, ***P < 0.001, Student's *t*-test).

Given that SETDB1 is physically associated with chromatin and functions as a transcriptional regulator (40), we examined whether SETDB1 directly occupies the promoters of differentially upregulated genes involved in the inflammation response. Chromatin immunoprecipitation coupled with quantitative PCR (ChIP–qPCR) showed little or no association of SETDB1 with these regions under both normoxic and hypoxic conditions (Supplementary Figure S7A), indicating that inflammation response-related genes are not direct targets of SETDB1. Next, we hypothesized that SETDB1 ablation could derepress TEs to produce dsRNA more robustly in hypoxia than normoxia and thus hyperactivates the inflammatory response under hypoxic conditions. Experiments to monitor derepression of TEs by immunostaining with J2 antibody specific for dsRNAs revealed higher intensity of J2 signals in SETDB1 KO cells under hypoxia relative to normoxia, supporting our hypothesis (Figure 5A, Supplementary Figure S7B).

Mammalian cells sense endogenous or exogenous dsRNAs via the dsRNA recognition receptors, MDA5 and RIG-1, and subsequently activate an inflammatory signal involving TBK1 (41). To explore whether dsRNA production is involved in inflammatory signaling in SETDB1 KO cells under hypoxic stress, we determined the requirement for MDA5 or RIG1 for stimulation of TBK1 activity and its downstream regulators p65 (NF-kB signaling) and IRF3 (IFN signaling). SETDB1 loss in normoxia induced phosphorylation of TBK1 and its downstream regulators to a slight extent, which was robustly enhanced in SETDB1 KO cells under hypoxic conditions (Figure 5B, Supplementary Figure S7C). However, KD of either MDA5 or RIG1 failed to enhance phosphorylation of TBK1 and its downstream signaling modulators in hypoxia, suggesting that dsRNAs generated by SETDB1 inactivation are involved in hyper-activation of the inflammatory responses under hypoxic conditions.

To examine whether TE derepression is related to dsRNA production due to SETDB1 loss in hypoxia, we analyzed 778 TE-derived transcripts detected using total RNA-seq (Supplementary Figure S7D). Unexpectedly, despite immunological detection of SETBD1 loss-induced increase in the global dsRNA content (Figures 5A, Supplementary Figure S7B), upregulated TEs in SETDB1 KO cells were not overrepresented compared with downregulated TEs either in normoxia or hypoxia (Supplementary Figure S7E). Next, we identified differentially expressed TEs (DETs, *P* < 0.05) in KO/Nor and KO/Hpx groups compared with control cells under normoxia (Con/Nor). KO/Nor cells exhibited 55 differentially upregulated TEs (DETups, A) and 70 differentially downregulated TEs (DETdns, C). The number of DETups and DETdns in KO/Hpx increased by ∼75% (96 DETups, B) and ∼13% (79 DETdns, D), respectively (Supplementary Figure S7F). Heatmap analysis showed that most of DETups in KO/Nor were also upregulated in KO/Hpx (Figure 5C). Moreover, cumulative expression of DETup genes in both groups A and B was significantly increased in KO/Hpx relative to KO/Nor (Figure 5D). By contrast, expression of DETdns in both groups C and D, downregulation of which was possibly mediated by a secondary effect of SETDB1 loss as shown previously (42), was not significantly different between KO/Nor and KO/Hpx (Supplementary Figure S7G, H). These results indicate that SETDB1 loss in hypoxia expands the number of derepressed TEs compared to that in normoxia, resulting in production of more dsRNAs in KO/Hpx than KO/Nor.

Next, we focused on the expression of six individual DETup genes (MER50B, LTR39, MamGypLTR1d, MER51a, HERVK9-int and LTR85a). Transcripts of all tested TEs were the most highly increased in SETDB1 KO cells under hypoxia (Figure 5E). ChIP–qPCR analysis showed that all TE regions were occupied by SETDB1 in normoxia and SETDB1 occupancy at these loci was significantly increased in hypoxia (Figure 5F). Concurrently, H3K9me3 levels were increased at these regions in hypoxia (Supplementary Figure S7I). Conversely, hypoxia-induced elevation of H3K9me3 was not detected at any of these locations in SETDB1 KO cells. By contrast, occupancy of SETDB1 and H3K9me3 in ACTB, whose expression is unrelated to SETDB1, was not significantly changed by oxygen availability (Figure 5F, Supplementary Figure S7I). This observation rules out the possibility that hypoxia-induced accumulation of SETDB1 and H3K9me3 is independent of genomic loci. Collectively, our results imply that enhanced accumulation of SETDB1, along with increased H3K9me3 levels, is required for maintaining the repressive state of TE elements in hypoxia.

### SETDB1 loss in hypoxia causes genome instability and subsequent cell death

**Figure 6.**
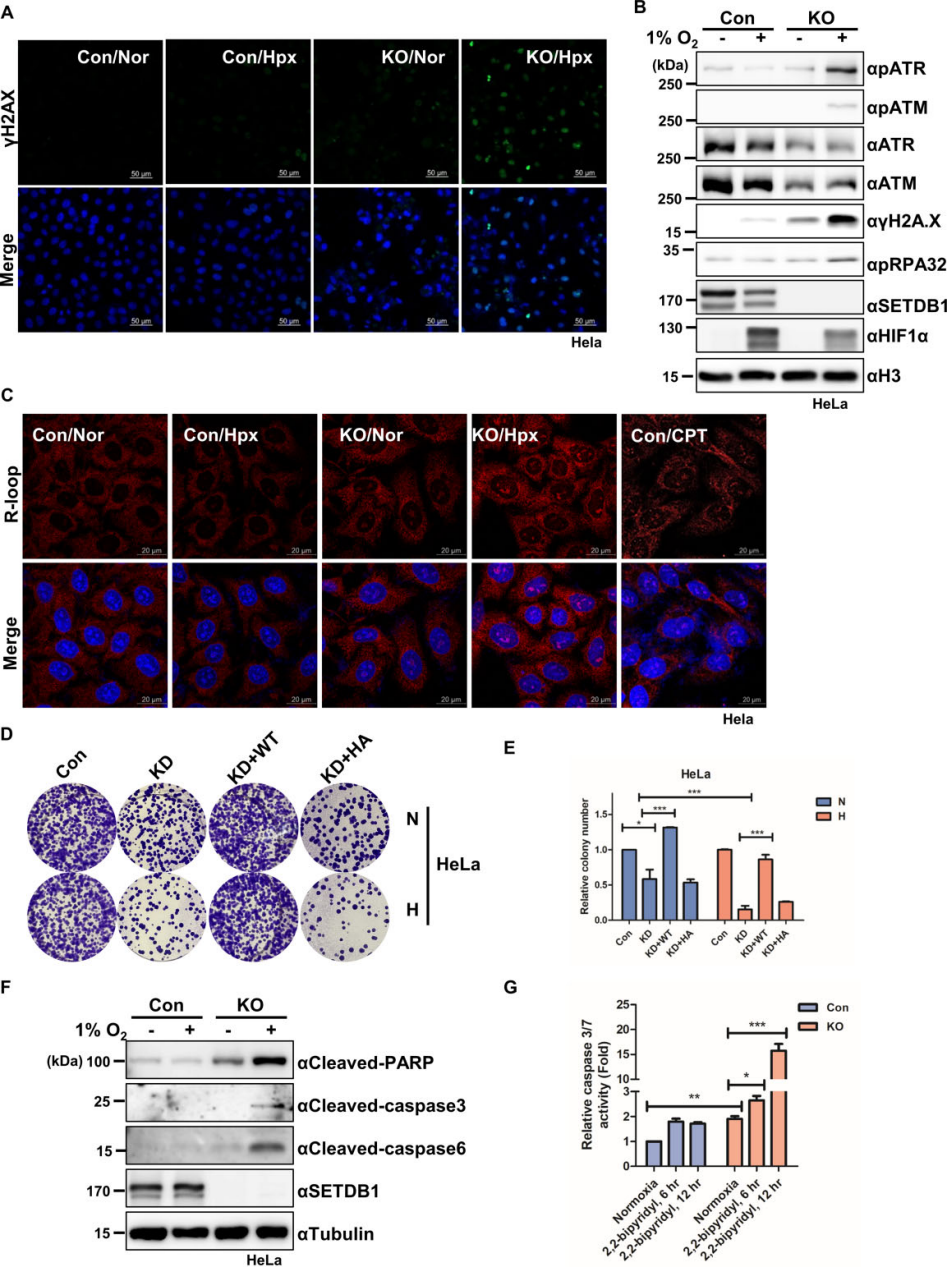
SETDB1 loss in hypoxia leads to cell death via induction of R-loops associated with genome instability **(A)** Representative images of immunostained γH2AX (green) and DAPI (blue) in SETDB1 KO (KO) and control (Con) HeLa cells under normoxia (Nor) or hypoxia conditions (Hpx, 1% O_2_ for 24 h). Scale bars denote 20 μm. **(B)** Immunoblotting of DNA damage markers in SETDB1 KO (KO) and control (Con) HeLa cells under normoxia or hypoxia (1% O_2_ for 24 h) with the indicated antibodies. **(C)** Representative images of immunostaining with R-loop (red) and DAPI (blue) in SETDB1 KO (KO) and control (Con) HeLa cells under normoxia (Nor) or hypoxia (Hpx, 1% O_2_ for 24 h). HeLa cells treated with camptothecin were stained as a positive control. Scale bars denote 20 μm. **(D)** Colony formation assay (CFA) in SETDB1-depeted (KD), control (Con), Wild-type (WT) SETDB1-rescued (Flag-SETDB1 overexpressing) and catalytic dead H1224A (HA) SETDB1-reintroduced HeLa cells exposed to hypoxia mimetic condition (2,2-bipyridyl, 200 μM for 24 h) followed by normoxic culture (medium change) for 24 d. N: normoxic condition; H: hypoxia mimetic condition. **(E)** Quantification of relative colony number as described in Figure 6D (*P < 0.05, **P < 0.01, ***P < 0.001, Student's *t*-test). **(F)** Immunoblot analysis of apoptosis markers in SETDB1 KO (KO) and control HeLa cells exposed to hypoxic stress (1% O_2_ for 12 h) with the indicated antibodies. **(G)** Caspase 3/7 activity in SETDB1 KO (KO) and control HeLa cells exposed to 2,2-bipyridyl (200 μM) for the indicated times assessed with the Caspase-Glo® 3/7 Assay. Relative luminescence units (RLU) reflecting caspase-3 and -7 activity are presented as mean ± SEM (*n* = 4, *P < 0.05, **P < 0.005, ***P < 0.001, Student's *t*-test).

Derepression of heterochromatic repetitive elements (RE), such as TEs, is correlated with genomic instability (19). Accordingly, we examined the hypothesis that enhanced derepression of TEs in SETDB1 KO cells under hypoxia could underlie damage to genome stability. In support of our hypothesis, immunostaining with γH2AX antibody, a representative DNA damage marker, showed a strong increase in γH2AX in SETDB1 KO cells under hypoxia (Figure 6A, Supplementary Figure S7J). Consistently, SETDB1 loss in hypoxia led to a marked increase in other DNA damage markers, such as phosphorylated forms of ATR, ATM and RPA32 (Figure 6B), indicating that SETDB1 inactivation mediates robust induction of DNA damage in hypoxia.

Previous studies have shown that deletion of the SETDB1 ortholog in *C. elegans* derepresses REs, thereby leading to accumulation of R-loops that induce DNA damage (19). Accordingly, we examined whether DNA damage in SETDB1 KO cells under hypoxic conditions is related to accumulation of R-loops mediated by RE derepression. Immunostaining with a R9.6 antibody specific for R-loops showed that SETDB1 loss induced R-loop formation in normoxia and to an even greater extent in hypoxia (Figure 6C, Supplementary Figure S7K). Furthermore, co-staining of γH2AX with 5′-ethynyl-2′-deoxyuridine (EdU) specific for cells in the S phase revealed that DNA damage in SETDB KO cells under hypoxic conditions occurred largely in the S phase where R-loops block replication fork progression (Supplementary Figure S7L).

DNA damage burden can impair cell growth and proliferation, leading to cell death. Thus, using colony-forming assays, we investigated whether SETDB1 ablation affects cancer cell proliferation in hypoxia. Exposure of HeLa and colon cancer cells (LS174T, SW620 and SW480) lacking SETDB1 to the hypoxia mimetic, 2,2-bipyridyl, for 24 h prior to culture in normoxia compromised colony formation to a greater extent than that observed in non-exposed cells (Figure 6D and E, Supplementary S7M–O). We observed that ectopic expression of wild-type SETDB1, but not the catalytically inactive H1224A SETDB1 mutant, rescued the proliferation defects in SETDB1 KD cells. A transcriptome analysis showed enrichment of apoptosis pathway-related genes in SETDB1-depleted cells (shSETDB1) under hypoxic conditions compared with that under normoxic conditions (Supplementary Figure S7P). Consistent with this, the molecular markers for cell death, cleaved PARP, caspase 3 and caspase , were increased in SETDB1-KO cells exposed to hypoxic stress (Figure 6F). Caspase 3/7 activity assays also showed that apoptosis in SETDB1-KO cells was increased by treatment with different hypoxia-mimetic reagents (Figure 6G, Supplementary Figure S7Q and R). Our findings collectively demonstrate that cells lacking SETDB1 undergo robust cell death under hypoxic stress that is associated with strong derepression of REs.

### SETDB1 expression is inversely correlated with the inflammatory response and innate immunity in hypoxia

To investigate whether SETDB1 is associated with hypoxic response in clinical samples, we analyzed transcriptomic features associated with its expression in patients with hypoxic cancer. We obtained data from 10 117 transcriptomes of 32 different cancer types in the TCGA PanCanAtlas (https://gdc.cancer.gov/about-data/publications/pancanatlas). Data were stratified into two groups based on a *Buffa* signature-based hypoxia scoring procedure (31–33): hypoxia (hypoxia score >0; 4984 samples) and normoxia (hypoxia score <0; 5133 samples; Supplementary Figure S8A). Whereas 729 genes showed significant differences between the hypoxia and normoxia groups, SETDB1 was not differentially expressed (Supplementary Figure S8B). This finding demonstrates that consistent with our *in vitro* results (Supplementary Figure S2C), hypoxia has little effect on SETDB1 transcription in patient samples. Functional enrichment analysis of 318 over-represented genes in the hypoxia group compared with the normoxia group showed that genes involved in cell cycle-related functions were significantly upregulated under hypoxia (Supplementary Figure S8C). Based on the results, we conclude that cancer cells under hypoxia tend to proliferate more aggressively than those under normoxia.

**Figure 7.**
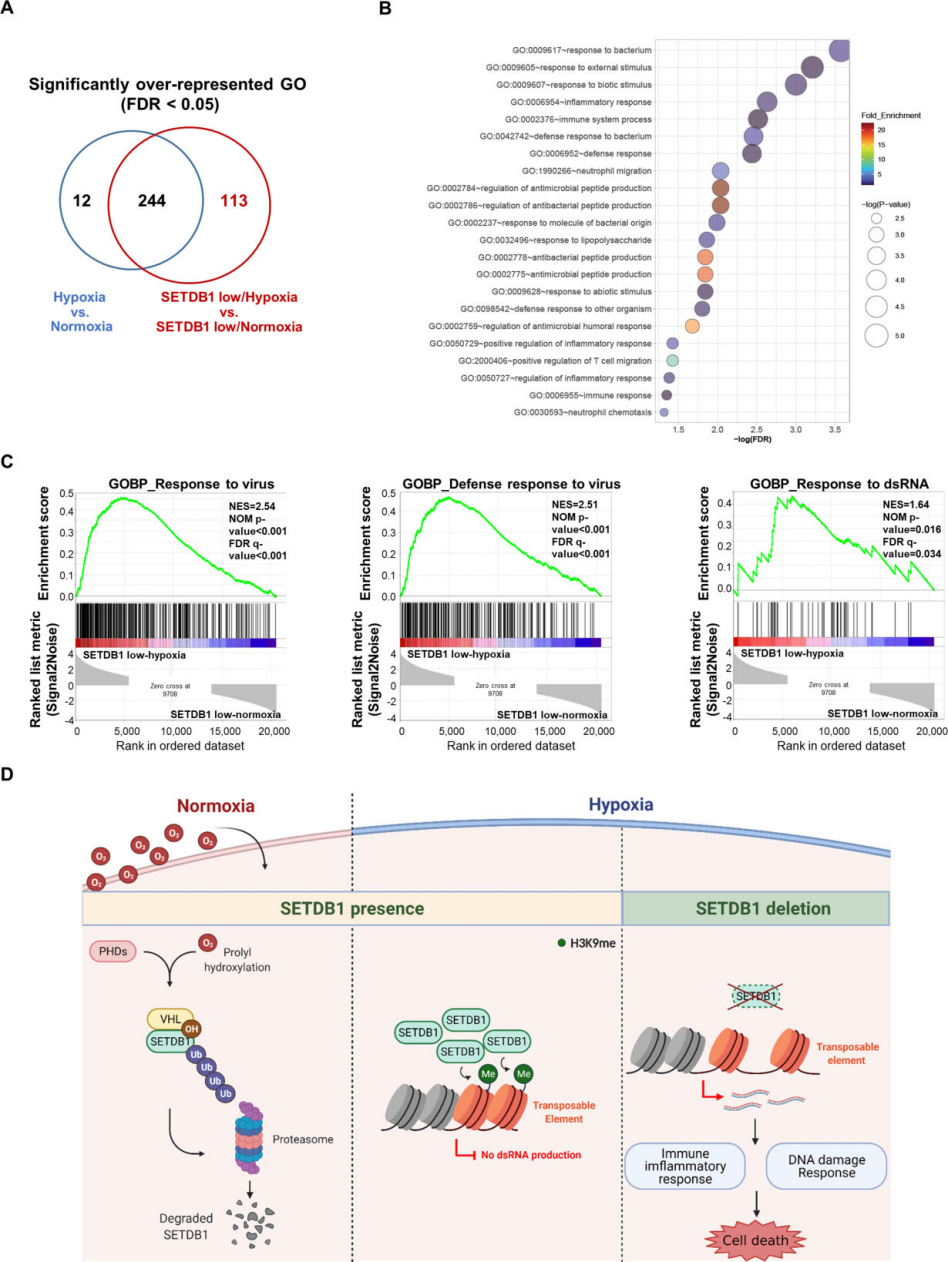
Transcriptomic profiling using patient data from the Pan-Cancer Atlas. **(A)** Venn diagram showing the number of differentially represented GO pathways across hypoxia versus normoxia, hypoxia with low SETDB1 expression versus normoxia with low SETDB1 expression and overlap between each set of groups. **(B)** Enriched GO pathways in the hypoxia with low-SETDB1 group. The size of the circles is consistent with –log (*P* value). **(C)** Representative enrichment plots from GSEA conducted for SETDB1 low-hypoxia versus SETDB1 low-normoxia groups. NES (normalized enrichment score), the normalized *P* values and FDR values are described. **(D)** Schematic illustration for oxygen-dependent regulation of SETDB1 function, generated with BioRender (http://biorender.com). In normoxia, PHDs hydroxylate proline residues of SETDB1 for VHL-mediated polyubiquitination followed by proteasomal degradation (left). In hypoxia, unhydroxylated SETDB1 is stabilized by escaping from VHL-mediated degradation. Increased SETDB1 leads to augmented SETDB1 occupancy at chromatin associated with TEs and subsequent strong repression of TEs via H3K9 methylation (middle). SETDB1 loss in hypoxia fails to silence TEs, thereby generating TE transcript-derived immune-inflammatory and DNA damage responses, with subsequent cell death (right).

To examine the functional implications of SETDB1 in hypoxic cancer, hypoxia and normoxia groups were further classified into high and low SETDB1 expression sub-groups. We identified 1220 differentially expressed genes in the hypoxia-low SETDB1 group compared with the normoxia-low SETDB1 group (two sample *t*-tests, *P* < 0.01, fold difference >3). Functional enrichment analysis of 622 overrepresented genes in the hypoxia-low SETDB1 group also showed association with cell cycle-related functions (Supplementary Figure S8D). To identify the biological processes specifically linked to SETDB1 expression in the hypoxia group, we excluded those that overlapped in the hypoxia versus normoxia and the hypoxia-low SETDB1 versus normoxia-low-SETDB1 groups (Figure 7A). Overall, functions related to inflammatory response and innate immunity were enriched specifically in the hypoxia-low SETDB1 group (Figure 7B). The data suggest that unlike cell cycle-related pathways, which are upregulated in hypoxic cancer irrespective of SETB1, activation of inflammatory and immune response functions is linked to low expression of SETDB1 in hypoxia. Interestingly, GSEA showed that responses to dsRNAs as well as viruses were upregulated in the hypoxia-low SETDB1 compared with normoxia-low SETDB1 group (Figure 7C). Our results clearly imply that the suppressive effects of SETDB1 on endogenous dsRNA production from TEs and subsequent inflammatory responses are more significant in hypoxic than normoxic cancer cells.

## Discussion

To ensure survival in challenging hypoxic tumor tissue environments, cancer cells reprogram transcriptional circuits via diverse regulatory pathways, including epigenetic alterations. In this study, several findings demonstrated that SETDB1-mediated TE repression is critical for genome stability in hypoxia (Figure 7D). First, SETDB1 was hydroxylated at proline residues by PHDs, which was recognized by the CRL2^VHL^ complex for proteasomal degradation. Second, under conditions of limited oxygen, accumulating SETDB1, freed from the CRL^VHL^ complex, facilitated H3K9 methylation to maintain the repressive state of transposable elements. Third, loss of SETDB1 failed to promote hypoxia-induced accumulation of H3K9 methylation on TEs, thereby derepressing TEs to generate more dsRNAs in hypoxia than normoxia. Consequently, SETDB1 inactivation in hypoxia hyperactivated the immune-inflammatory response and induced robust cell death. Finally, transcriptome analysis of human cancer disclosed that SETDB1 expression is inversely associated with dsRNA responses along with innate immune-inflammatory pathways in hypoxic cancer.

SETDB1 expression is frequently upregulated in various cancers (12–16). In addition, SETDB1 is functionally involved in different aspects of tumorigenesis, including suppressive anti-tumor immunity (43). SETDB1 abundance is therefore significantly implicated in oncogenic progression. Here, we identified CRL2^VHL^-dependent proteasomal degradation as a crucial regulator of SETDB1 protein homeostasis. SETDB1 accumulation may underlie a molecular mechanism for VHL inactivation involved in cancer progression. In keeping with these findings, the hypoxia-induced increase of SETDB1 protein suggests an oncogenic function in an oxygen-limited tumor microenvironment (TME). Inactivation of KDMs and TETs in hypoxia facilitates alterations in chromatin structure that are distinct from those found in normoxic conditions (11,44). Interestingly, our experiments showed that oxygen depletion intensified the effect of SETDB1 loss on derepression of TEs. This finding suggests that chromatin state of TEs in hypoxia may not be the same as that in normoxia and additional factors are required for stable repression of TEs. Unfortunately, because the 3PA mutant SETDB1 without VHL-interacting hydroxyl proline residues lacks intrinsic methyltransferase activity, we were unable to directly assess whether TE repression requires an increase in the catalytic activity of SETDB1 under hypoxic conditions. Nevertheless, the accumulation of SETDB1 together with H3K9me3 on repressive TE loci in hypoxia implies that maintaining TE chromatin status in the context of limited oxygen availability is associated with an increase in SETDB1 levels. We speculate that hypoxia modulates the activities of chromatin-regulating enzymes including KDMs and TETs and alters the structure of TE-associated chromatin to increase susceptibility to SETDB1 loss. Accordingly, repression of TEs in hypoxia may require elevated SETDB1-mediated H3K9 methylation compared with that in normoxia (45).

To overcome the oncogenic effect, several strategies, such as treatment with hypoxia-activated prodrugs, inhibition of HIF signaling and downstream metabolic interventions, have been proposed (46,47). Our demonstration that SETDB1 loss in hypoxia robustly de-represses TEs, triggering DNA damage-induced cell death, suggests that targeting SETDB1 could serve as a useful therapeutic strategy for directed treatment of cancer cells in the hypoxic TME. Recently, the utility of SETDB1 as a potent therapeutic target in combination with immune checkpoint blockade was highlighted based on the finding that SETDB1 inactivation generated TE-encoded antigens to boost tumor immunogenicity (43,45). Experiments from the current study showed that SETDB1 loss in hypoxia hyperactivates immune-inflammatory responses by increasing TE-derived dsRNAs, suggesting that combination of immune checkpoint blockade and SETDB1 inactivation is more efficacious for cancer cells with hypoxic TME. Given that the impact of hypoxic TME on the efficacy of anti-tumor immunotherapy has not yet been considered, further research focus on the identification of potent SETDB1 inhibitors and analysis of their efficacy for hypoxic cancer cells, either alone or in combination with immunotherapy, should aid in the improvement of anti-cancer strategies.

## Supplementary Material

gkad796_Supplemental_File

## Data Availability

The sequencing data generated for this study are available in the Gene Expression Omnibus (GEO) database (GSE179329 and GSE179327). Raw sequence tags were deposited in the NCBI Short Read Archive (SRA) under accession numbers SRP326659 and SRP326663. The mass spectrometry proteomics data have been deposited to the ProteomeXchange Consortium via the PRIDE partner repository (https://www.ebi.ac.uk/pride/archive/) with the dataset identifier PXD037104. The data underlying this article will be shared on reasonable request to the corresponding author.
