# Supplementary material for: Hypoxia stabilizes SETDB1 to maintain genome stability
Source: Nucleic Acids Res. 2023 Oct 18;51(20):11178–96. doi: 10.1093/nar/gkad796 (PMC10639076; doi:10.1093/nar/gkad796)
Supplement: gkad796_Supplemental_File [file gkad796_supplemental_file.pdf]

Fig S1.

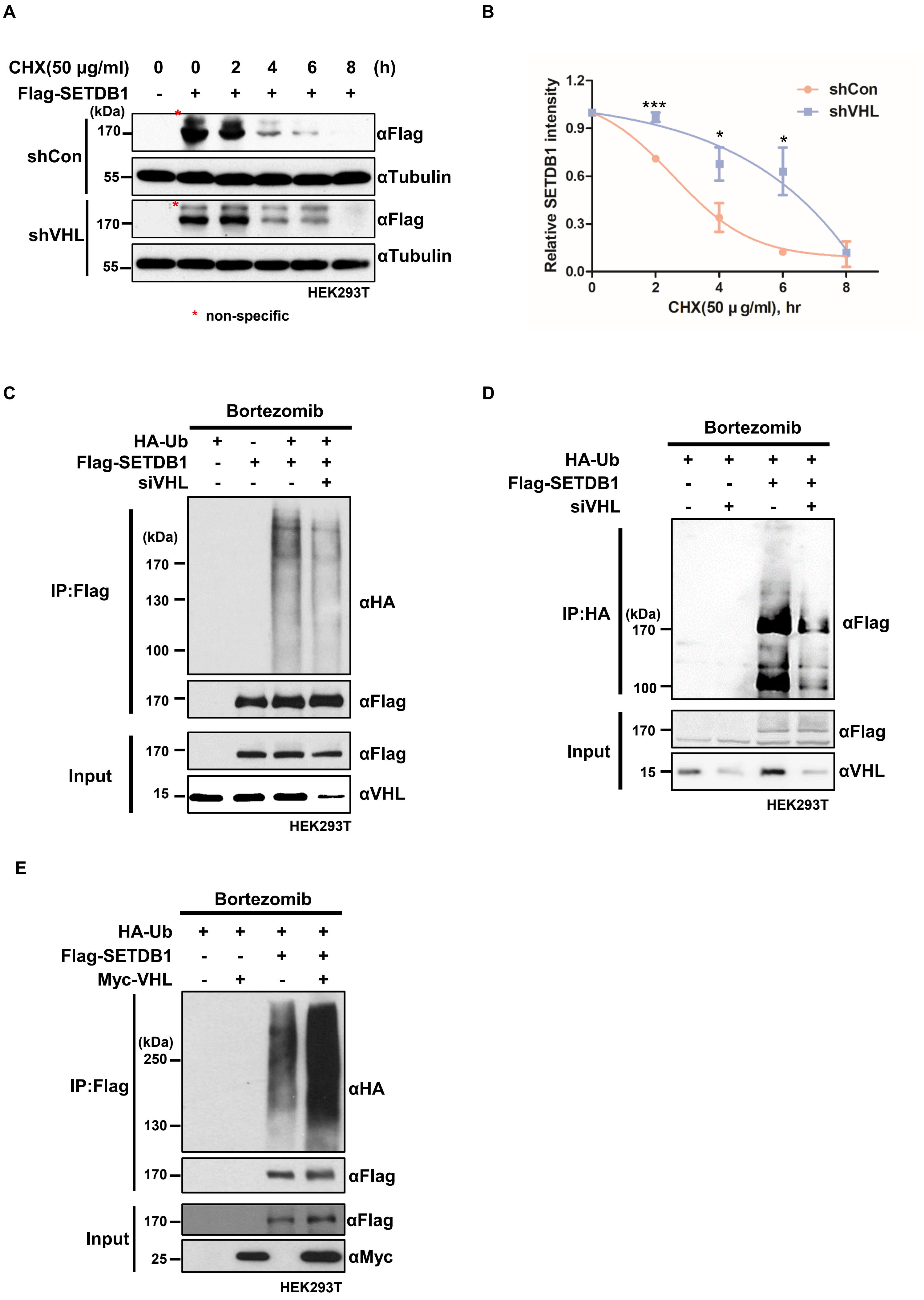
A
CHX(50 μg/ml) 0 0 2 4 6 8 (h)
Flag-SETDB1 - + + + + +
(kDa)
shCon
shVHL
170
55
αFlag
αTubulin
HEK293T
* non-specific
B
Relative SETDB1 intensity
CHX(50 μ g/ml), hr
shCon
shVHL
***
*
C
Bortezomib
HA-Ub
Flag-SETDB1
siVHL
IP:Flag
Input
αHA
αFlag
αVHL
HEK293T
D
Bortezomib
HA-Ub
Flag-SETDB1
siVHL
IP:HA
Input
αFlag
αVHL
HEK293T
E
Bortezomib
HA-Ub
Flag-SETDB1
Myc-VHL
IP:Flag
Input
αHA
αFlag
αMyc
HEK293T

Fig S2.

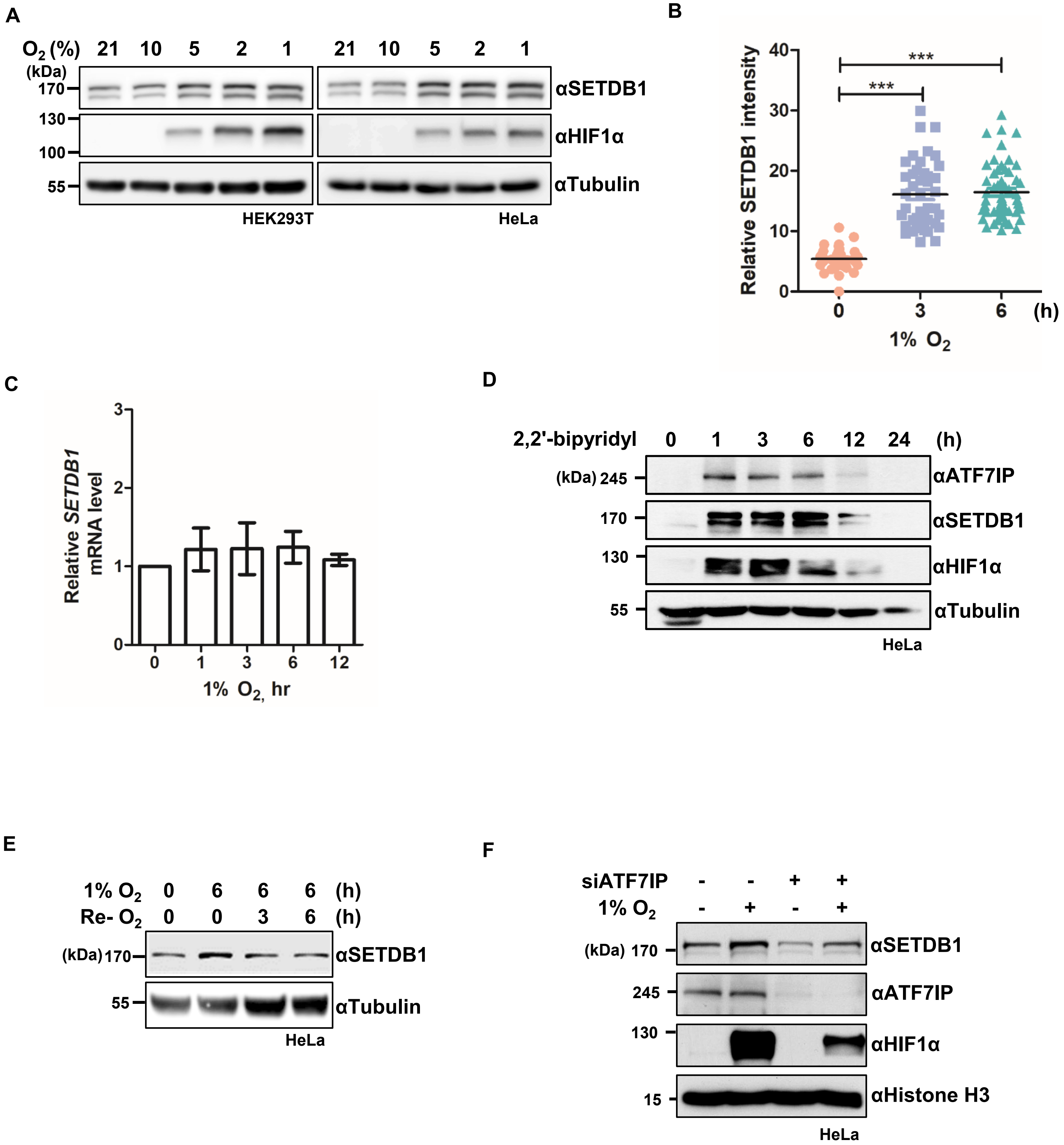
A
O2 (%) 21 10 5 2 1 21 10 5 2 1
(kDa)
170
130
100
55
αSETDB1
αHIF1α
αTubulin
HEK293T
HeLa
B
Relative SETDB1 intensity
40
30
20
10
0
***
***
0 3 6 (h)
1% O2
C
Relative SETDB1 mRNA level
3
2
1
0
0 1 3 6 12
1% O2, hr
D
2,2'-bipyridyl 0 1 3 6 12 24 (h)
(kDa) 245
170
130
55
αATF7IP
αSETDB1
αHIF1α
αTubulin
HeLa
E
1% O2 0 6 6 6 (h)
Re- O2 0 0 3 6 (h)
(kDa) 170
55
αSETDB1
αTubulin
HeLa
F
siATF7IP - - + +
1% O2 - + - +
(kDa) 170
245
130
15
αSETDB1
αATF7IP
αHIF1α
αHistone H3
HeLa

Fig S3.

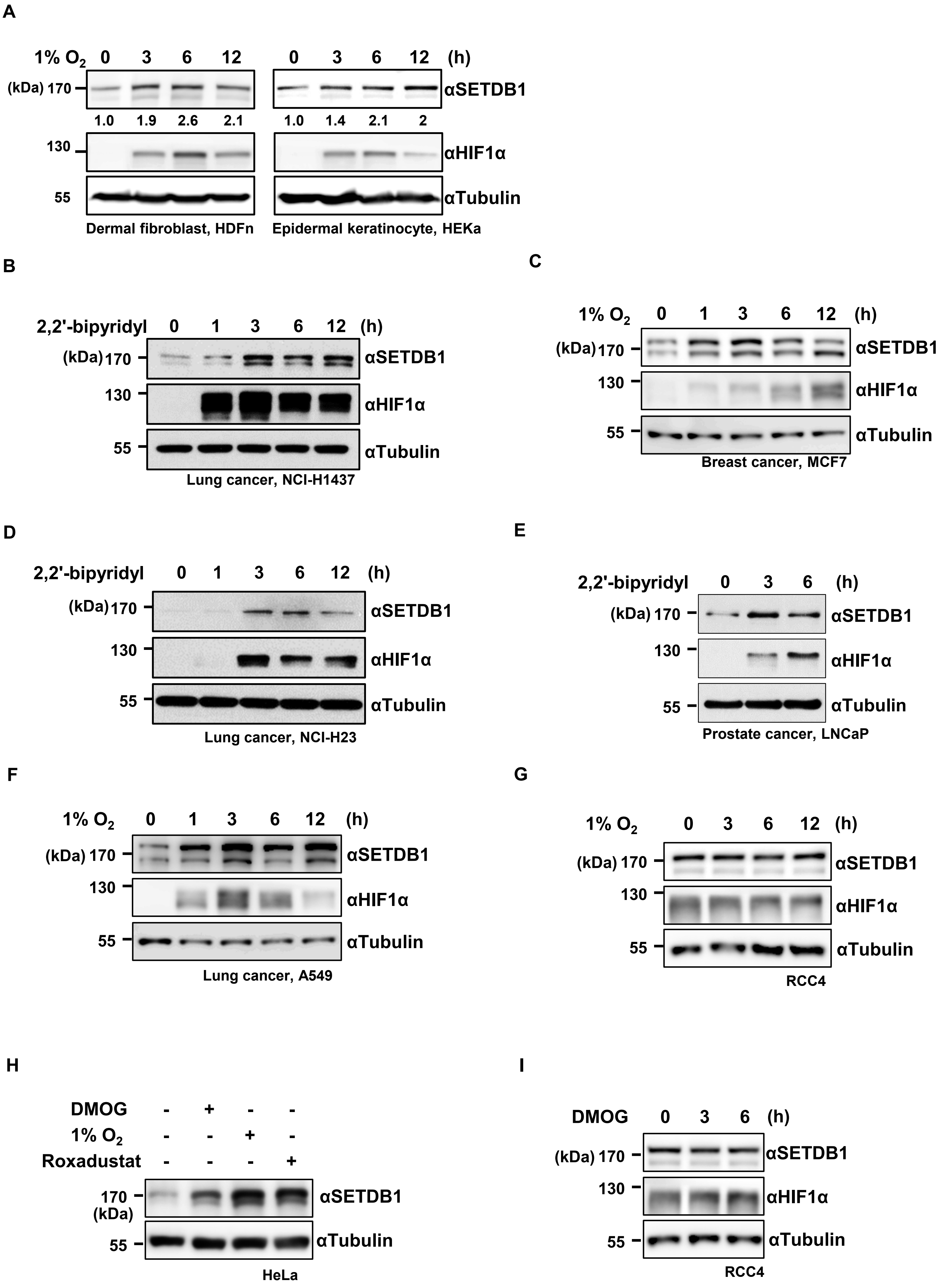
A
1% O2 0 3 6 12
0 3 6 12 (h)
(kDa) 170
αSETDB1
1.0 1.9 2.6 2.1
1.0 1.4 2.1 2
130
αHIF1α
55
αTubulin
Dermal fibroblast, HDFn
Epidermal keratinocyte, HEKa
B
2,2'-bipyridyl 0 1 3 6 12 (h)
(kDa) 170
αSETDB1
130
αHIF1α
55
αTubulin
Lung cancer, NCI-H1437
C
1% O2 0 1 3 6 12 (h)
(kDa) 170
αSETDB1
130
αHIF1α
55
αTubulin
Breast cancer, MCF7
D
2,2'-bipyridyl 0 1 3 6 12 (h)
(kDa) 170
αSETDB1
130
αHIF1α
55
αTubulin
Lung cancer, NCI-H23
E
2,2'-bipyridyl 0 3 6 (h)
(kDa) 170
αSETDB1
130
αHIF1α
55
αTubulin
Prostate cancer, LNCaP
F
1% O2 0 1 3 6 12 (h)
(kDa) 170
αSETDB1
130
αHIF1α
55
αTubulin
Lung cancer, A549
G
1% O2 0 3 6 12 (h)
(kDa) 170
αSETDB1
130
αHIF1α
55
αTubulin
RCC4
H
DMOG - + - -
1% O2 - - + -
Roxadustat - - - +
170
(kDa)
αSETDB1
55
αTubulin
HeLa
I
DMOG 0 3 6 (h)
(kDa) 170
αSETDB1
130
αHIF1α
55
αTubulin
RCC4

Fig S3.

J

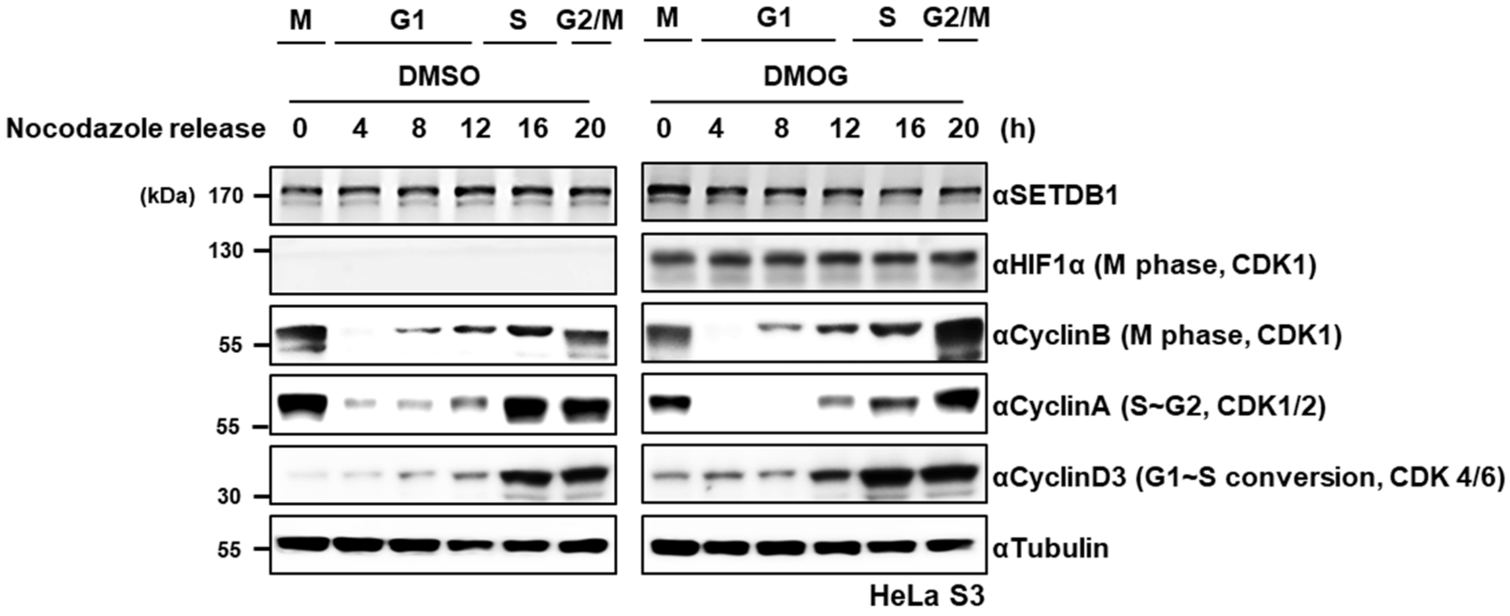
M
G1
S
G2/M
M
G1
S
G2/M
DMSO
DMOG
Nocodazole release 0 4 8 12 16 20 0 4 8 12 16 20 (h)
(kDa) 170
130
55
55
30
55
αSETDB1
αHIF1α (M phase, CDK1)
αCyclinB (M phase, CDK1)
αCyclinA (S~G2, CDK1/2)
αCyclinD3 (G1~S conversion, CDK 4/6)
αTubulin
HeLa S3

K

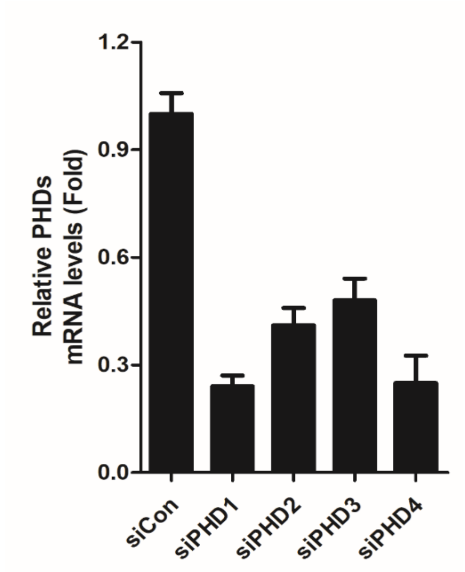
1.2
0.9
0.6
0.3
0.0
Relative PHDs
mRNA levels (Fold)
siCon
siPHD1
siPHD2
siPHD3
siPHD4

L

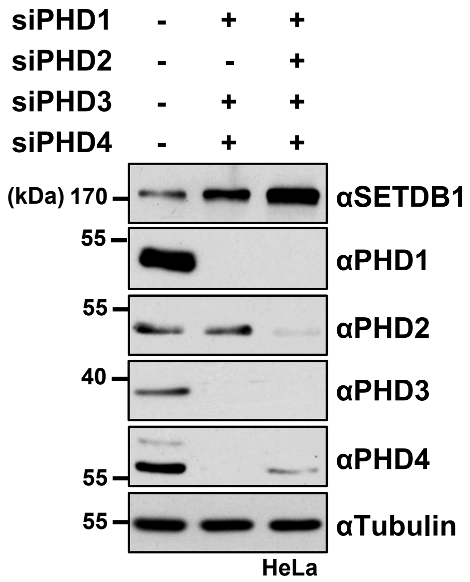
siPHD1 - + +
siPHD2 - - +
siPHD3 - + +
siPHD4 - + +
(kDa) 170
55
55
40
55
55
αSETDB1
αPHD1
αPHD2
αPHD3
αPHD4
αTubulin
HeLa

Fig S4.

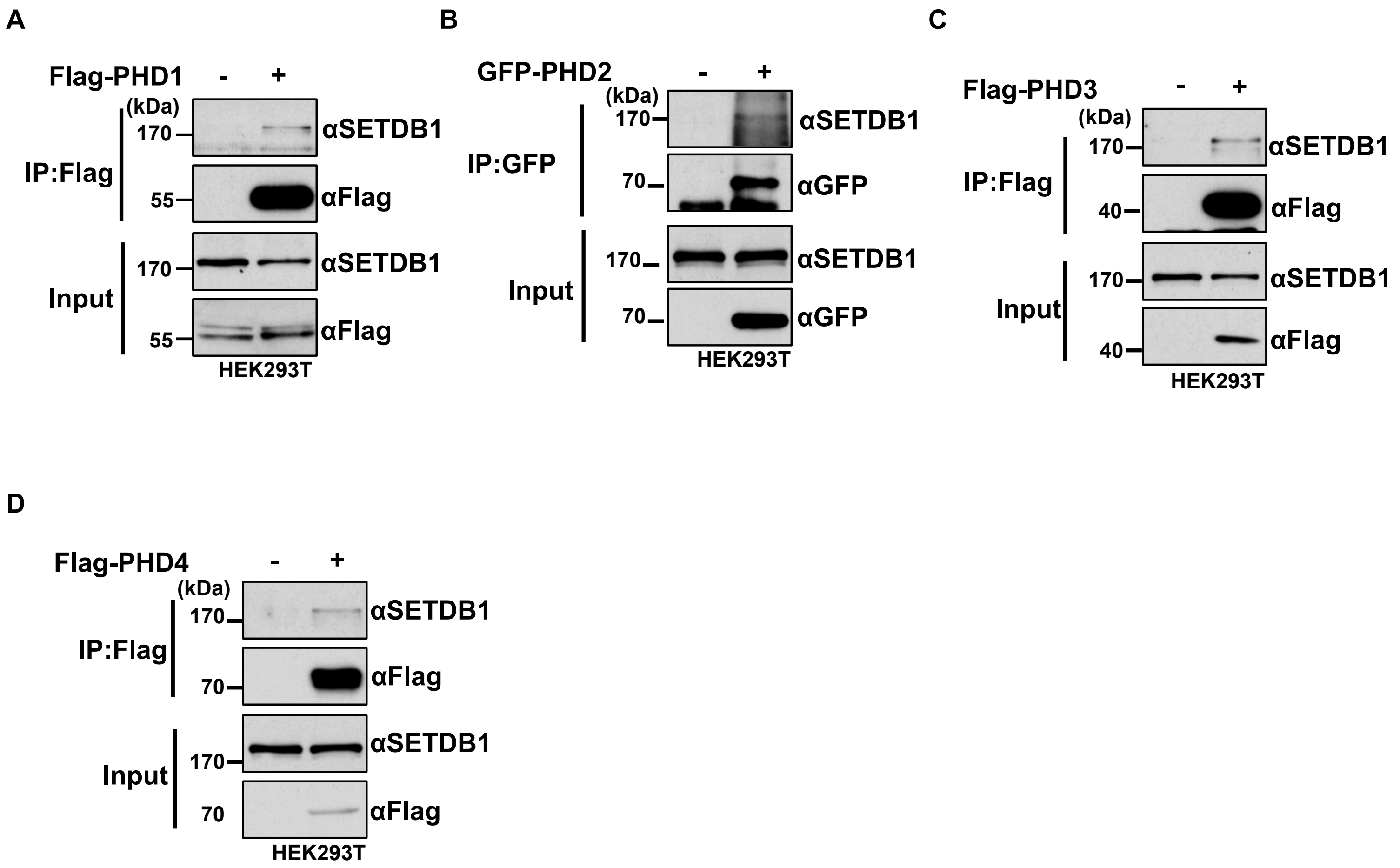
A
Flag-PHD1 - +
(kDa)
170
55
170
55
IP:Flag
Input
αSETDB1
αFlag
αSETDB1
αFlag
HEK293T
B
GFP-PHD2 - +
(kDa)
170
70
170
70
IP:GFP
Input
αSETDB1
αGFP
αSETDB1
αGFP
HEK293T
C
Flag-PHD3 - +
(kDa)
170
40
170
40
IP:Flag
Input
αSETDB1
αFlag
αSETDB1
αFlag
HEK293T
D
Flag-PHD4 - +
(kDa)
170
70
170
70
IP:Flag
Input
αSETDB1
αFlag
αSETDB1
αFlag
HEK293T

Fig S5.

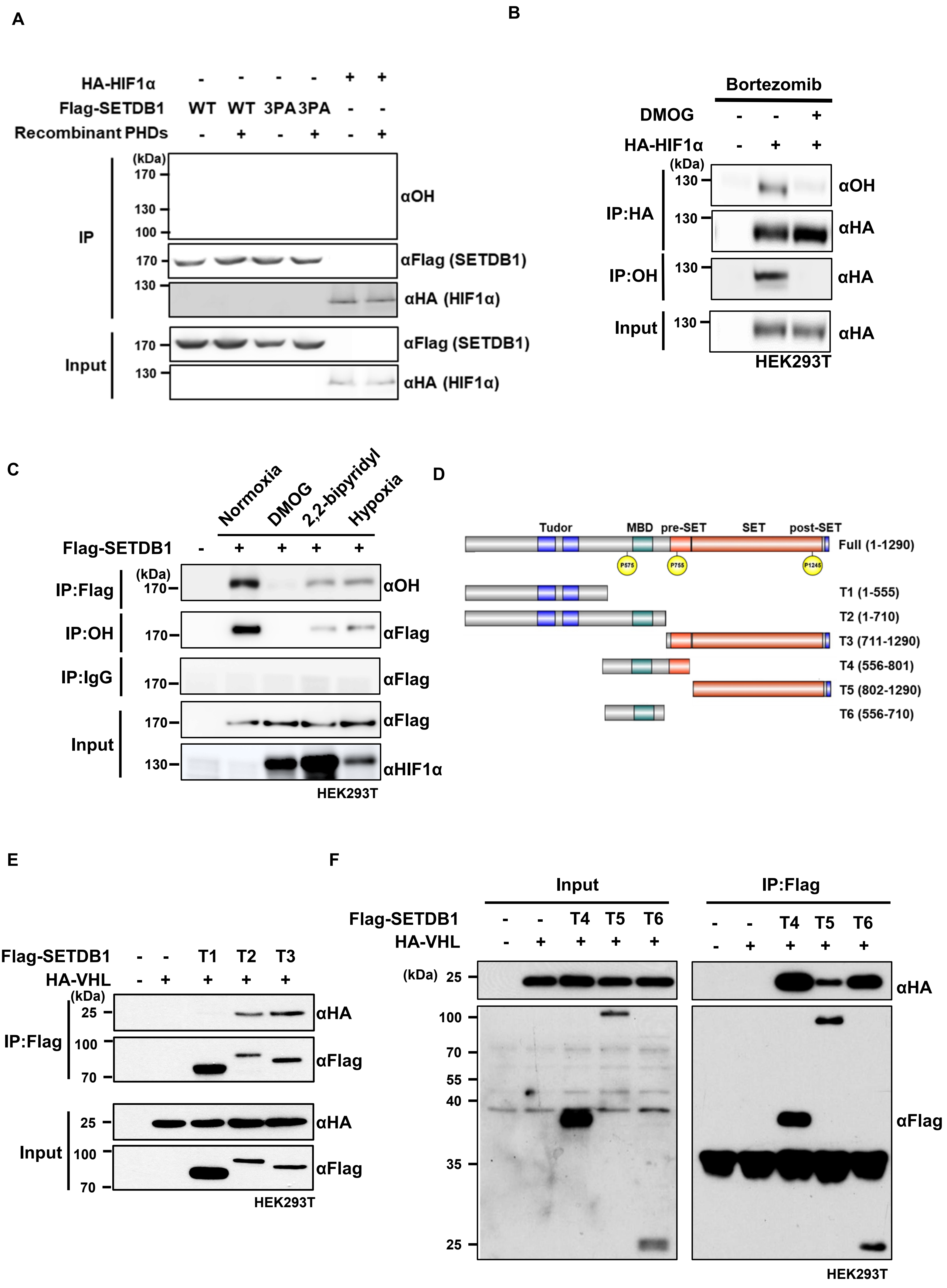

A
HA-HIF1α - - - - + +
Flag-SETDB1 WT WT 3PA 3PA - -
Recombinant PHDs - + - + - +
(kDa)
170
130
100
IP
αOH
αFlag (SETDB1)
αHA (HIF1α)
Input
αFlag (SETDB1)
αHA (HIF1α)
B
Bortezomib
DMOG - - +
HA-HIF1α - + +
IP:HA
αOH
αHA
IP:OH
αHA
Input
αHA
HEK293T
C
Normoxia
DMOG
2,2-bipyridyl
Hypoxia
Flag-SETDB1 - + + + +
IP:Flag
αOH
IP:OH
αFlag
IP:IgG
αFlag
Input
αFlag
αHIF1α
HEK293T
D
Tudor
MBD
pre-SET
SET
post-SET
Full (1-1290)
P575
P755
P1245
T1 (1-555)
T2 (1-710)
T3 (711-1290)
T4 (556-801)
T5 (802-1290)
T6 (556-710)
E
Flag-SETDB1 - - T1 T2 T3
HA-VHL - + + + +
IP:Flag
αHA
αFlag
Input
αHA
αFlag
HEK293T
F
Input
IP:Flag
Flag-SETDB1 - - T4 T5 T6
HA-VHL - + + + +
αHA
αFlag
HEK293T

Fig S5.

G

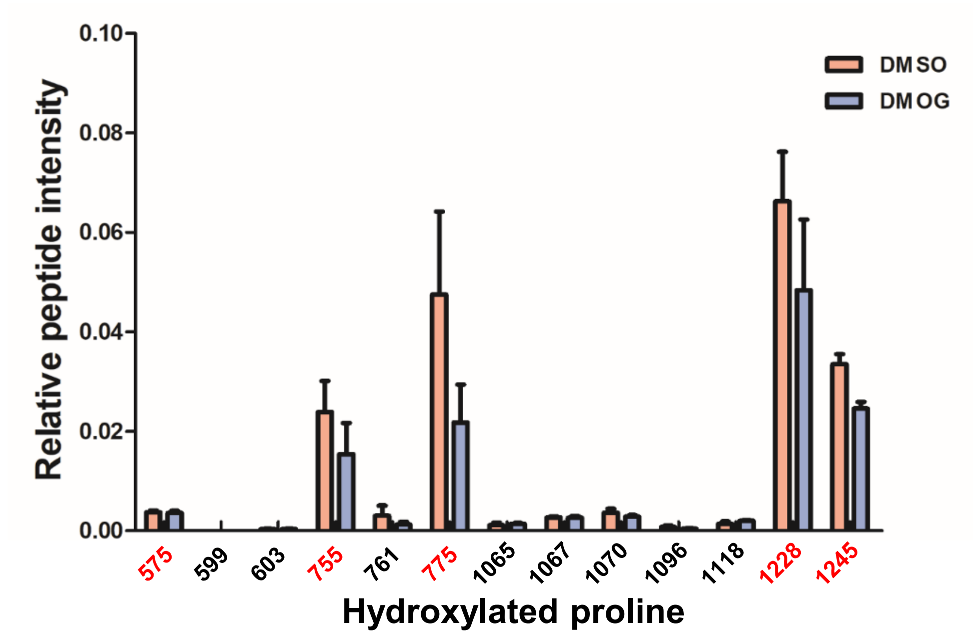

Relative peptide intensity
DMSO
DMOG
575
599
603
755
761
775
1065
1067
1070
1096
1118
1228
1245
Hydroxylated proline

H

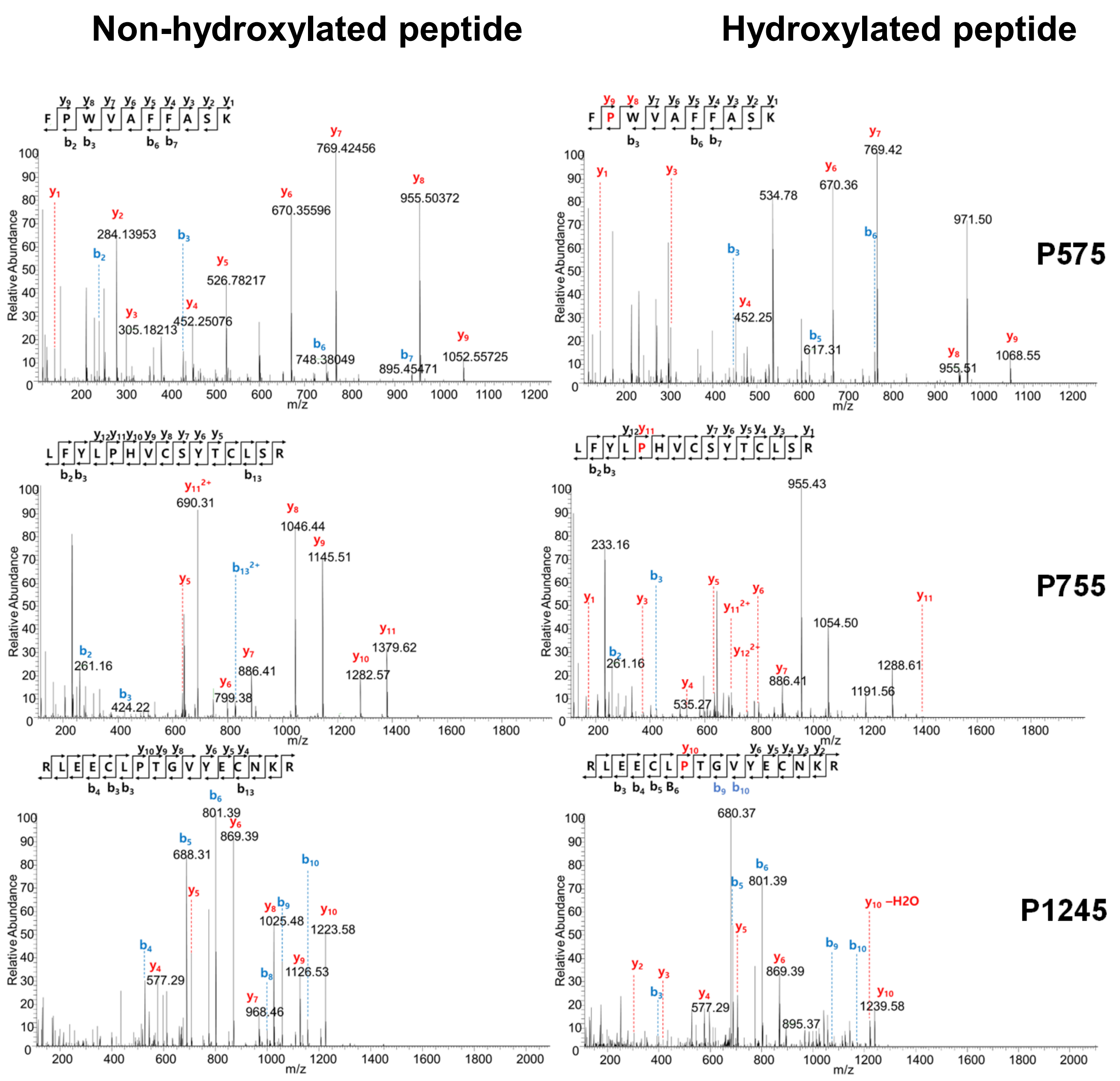

Non-hydroxylated peptide
Hydroxylated peptide
P575
P755
P1245
Relative Abundance
m/z

Fig S5.

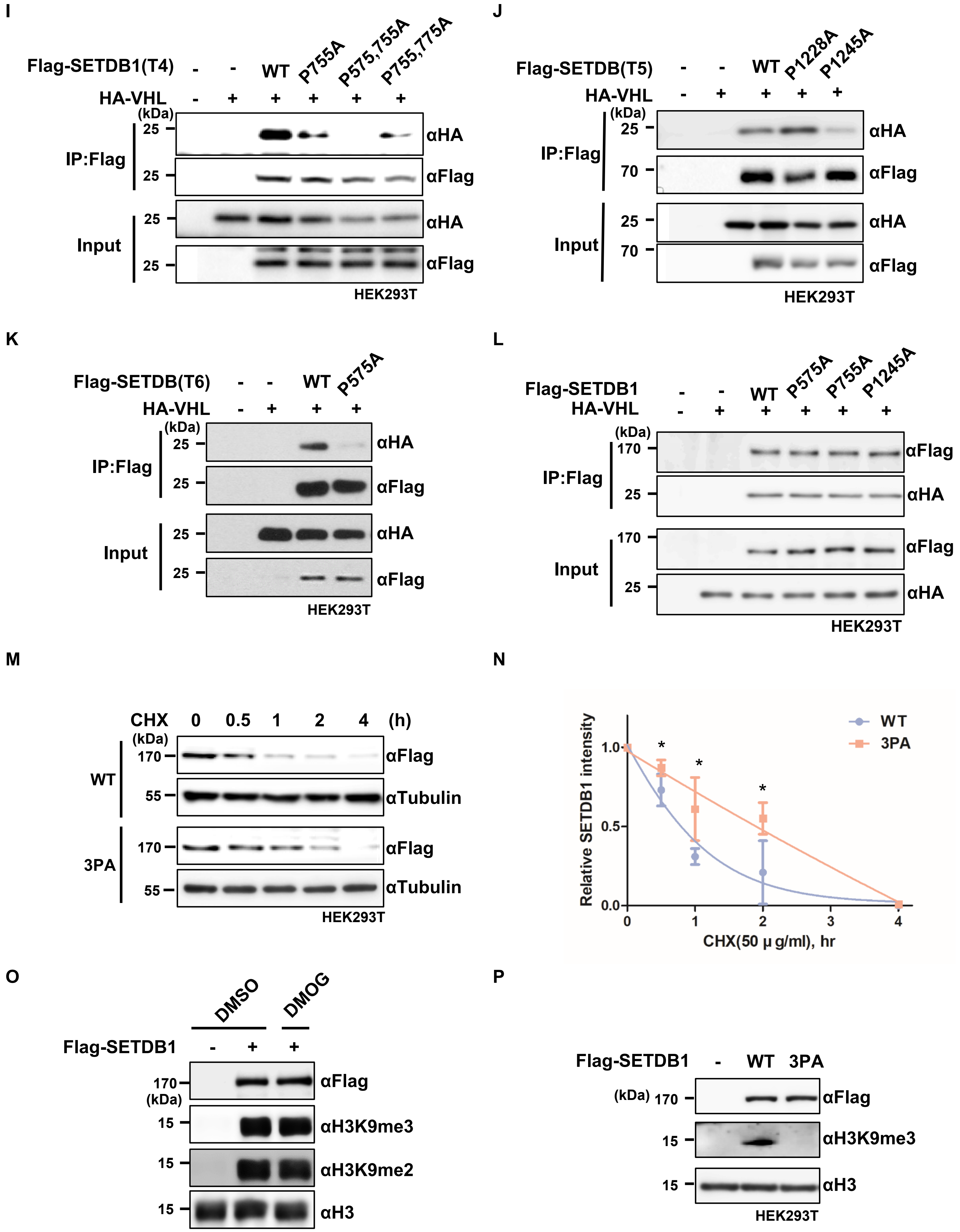
I
Flag-SETDB1(T4) - - WT P755A P575,755A P755,775A
HA-VHL - + + + + +
(kDa)
IP:Flag
Input
αHA
αFlag
HEK293T
J
Flag-SETDB(T5) - - WT P1228A P1245A
HA-VHL - + + + +
IP:Flag
Input
αHA
αFlag
HEK293T
K
Flag-SETDB(T6) - - WT P575A
HA-VHL - + + +
IP:Flag
Input
αHA
αFlag
HEK293T
L
Flag-SETDB1 - - WT P575A P755A P1245A
HA-VHL - + + + + +
IP:Flag
Input
αFlag
αHA
HEK293T
M
CHX 0 0.5 1 2 4 (h)
WT
3PA
αFlag
αTubulin
HEK293T
N
WT
3PA
Relative SETDB1 intensity
CHX(50 μ g/ml), hr
O
DMSO
DMOG
Flag-SETDB1 - + +
αFlag
αH3K9me3
αH3K9me2
αH3
P
Flag-SETDB1 - WT 3PA
αFlag
αH3K9me3
αH3
HEK293T

Fig S6.

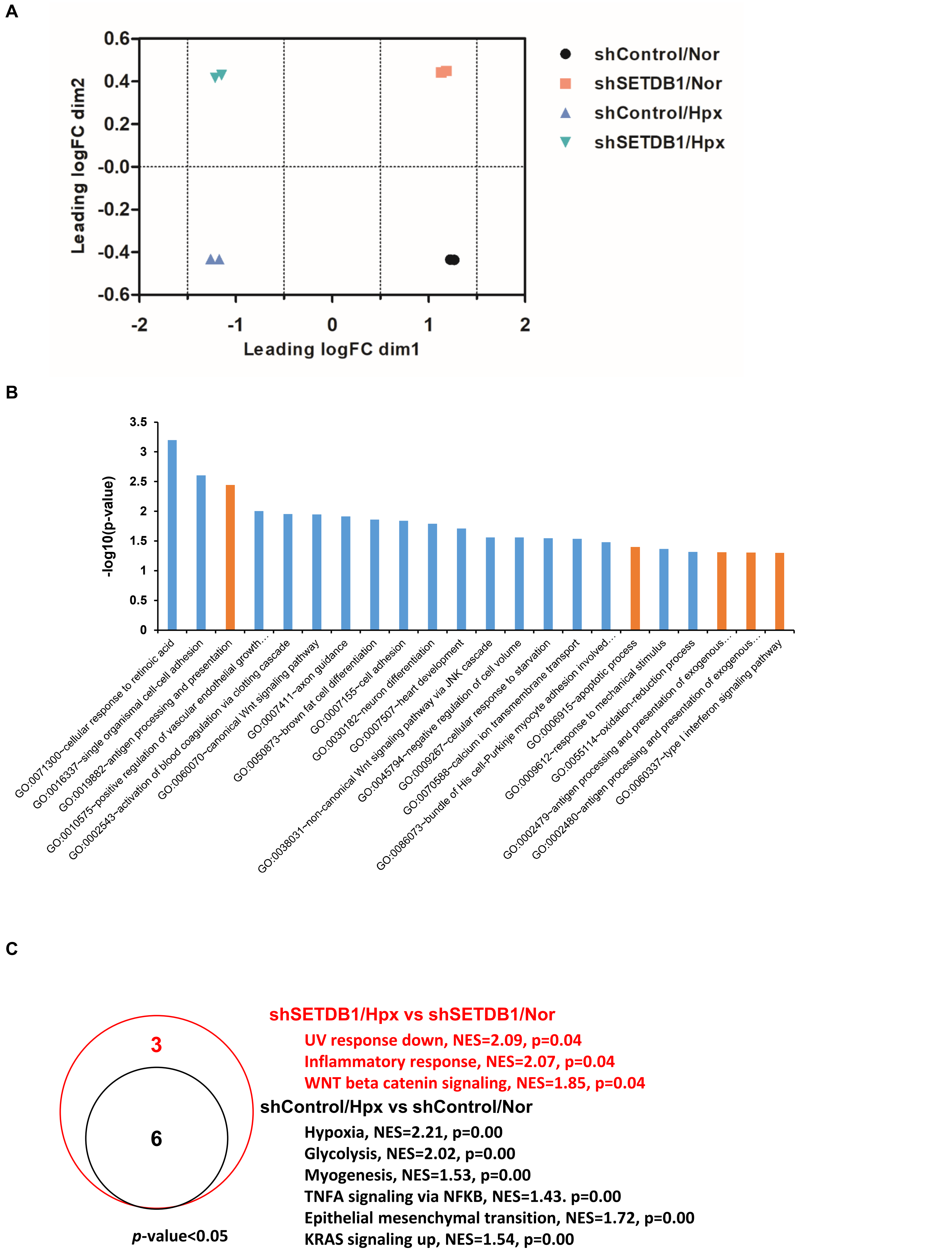
A
0.6
0.4
0.2
-0.0
-0.2
-0.4
-0.6
Leading logFC dim2
-2
-1
0
1
2
Leading logFC dim1
shControl/Nor
shSETDB1/Nor
shControl/Hpx
shSETDB1/Hpx
B
-log10(p-value)
3.5
3
2.5
2
1.5
1
0.5
0
GO:0071300~cellular response to retinoic acid
GO:0016337~single organismal cell-cell adhesion
GO:0019882~antigen processing and presentation
GO:0010575~positive regulation of vascular endothelial growth…
GO:0002543~activation of blood coagulation via clotting cascade
GO:0060070~canonical Wnt signaling pathway
GO:0007411~axon guidance
GO:0050873~brown fat cell differentiation
GO:0007155~cell adhesion
GO:0030182~neuron differentiation
GO:0007507~heart development
GO:0038031~non-canonical Wnt signaling pathway via JNK cascade
GO:0045794~negative regulation of cell volume
GO:0009267~cellular response to starvation
GO:0070588~calcium ion transmembrane transport
GO:0086073~bundle of His cell-Purkinje myocyte adhesion involved…
GO:0006915~apoptotic process
GO:0009612~response to mechanical stimulus
GO:0055114~oxidation-reduction process
GO:0002479~antigen processing and presentation of exogenous…
GO:0002480~antigen processing and presentation of exogenous…
GO:0060337~type I interferon signaling pathway
C
3
6
p-value<0.05
shSETDB1/Hpx vs shSETDB1/Nor
UV response down, NES=2.09, p=0.04
Inflammatory response, NES=2.07, p=0.04
WNT beta catenin signaling, NES=1.85, p=0.04
shControl/Hpx vs shControl/Nor
Hypoxia, NES=2.21, p=0.00
Glycolysis, NES=2.02, p=0.00
Myogenesis, NES=1.53, p=0.00
TNFA signaling via NFKB, NES=1.43. p=0.00
Epithelial mesenchymal transition, NES=1.72, p=0.00
KRAS signaling up, NES=1.54, p=0.00

Fig S6.

D

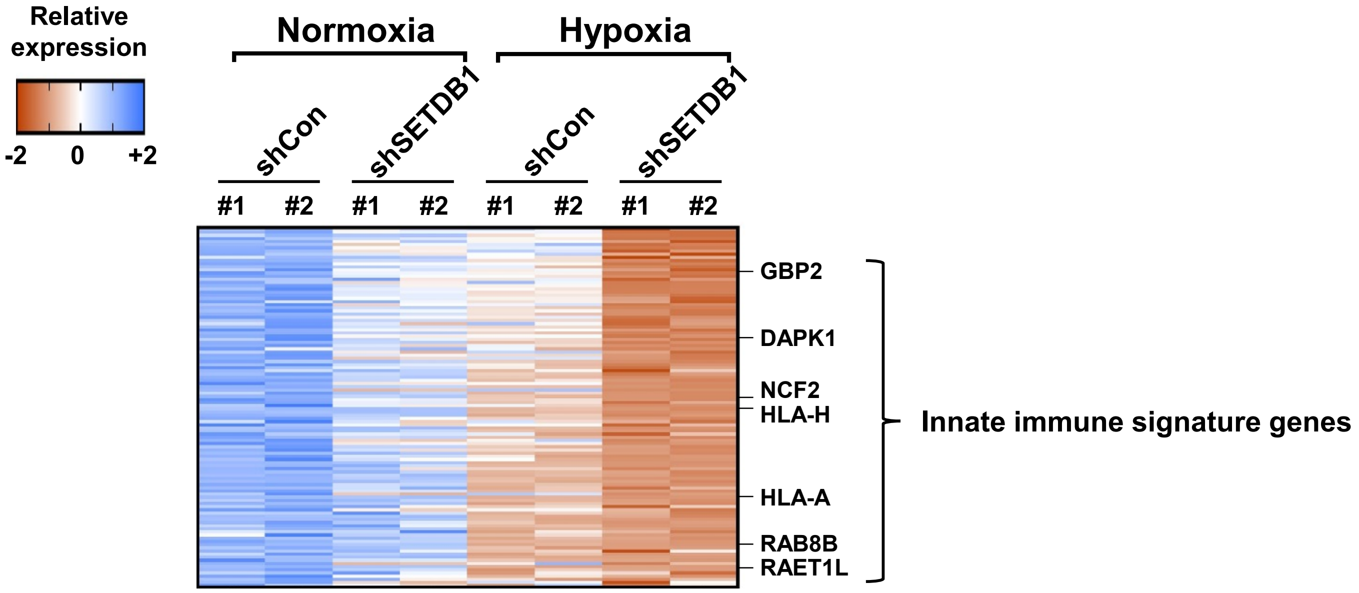
Relative expression
-2
0
+2
Normoxia
Hypoxia
shCon
shSETDB1
shCon
shSETDB1
#1
#2
#1
#2
#1
#2
#1
#2
GBP2
DAPK1
NCF2
HLA-H
HLA-A
RAB8B
RAET1L
Innate immune signature genes

Fig S7.

A

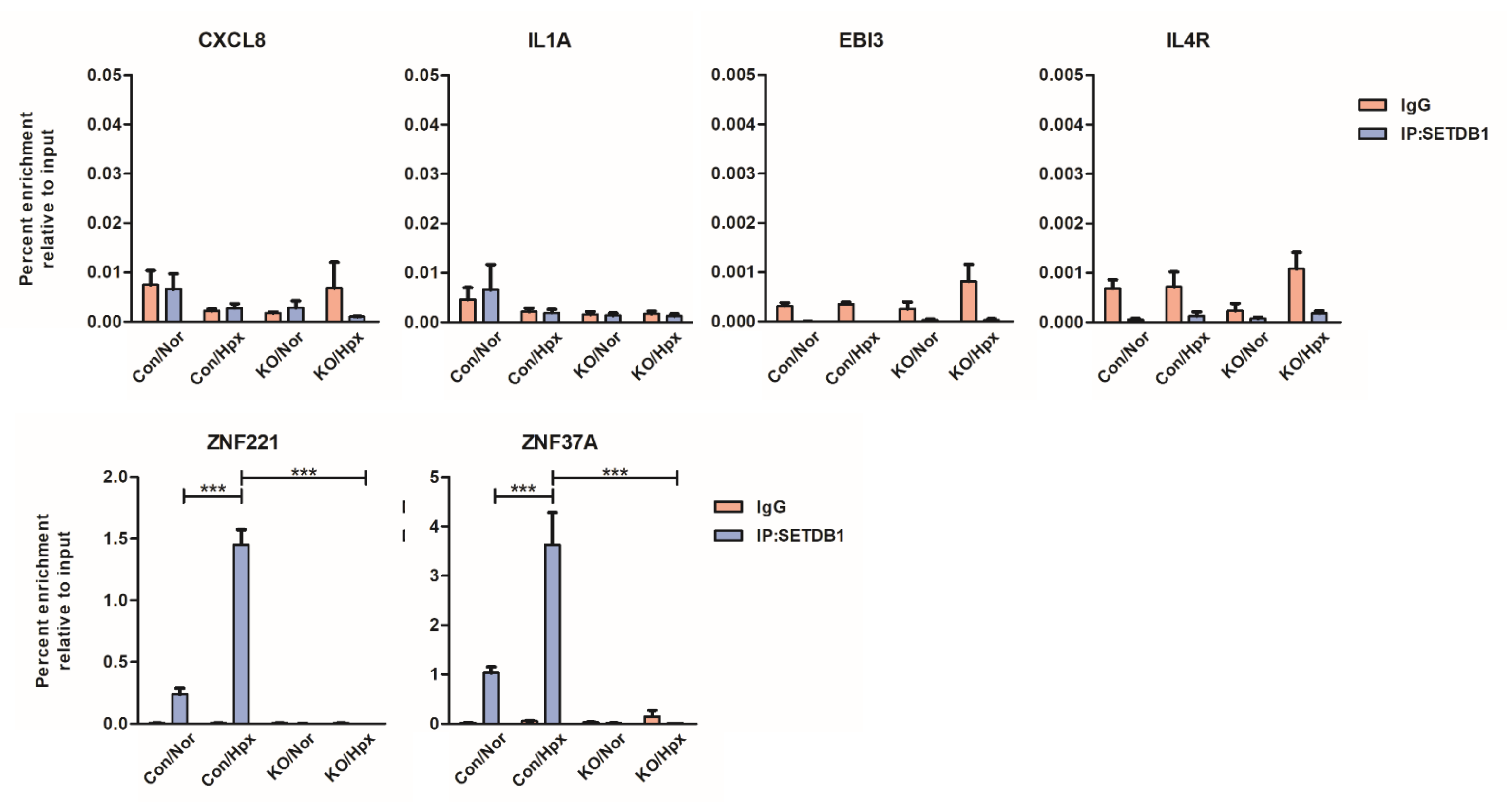
CXCL8
IL1A
EBI3
IL4R
Percent enrichment relative to input
0.05
0.04
0.03
0.02
0.01
0.00
0.005
0.004
0.003
0.002
0.001
0.000
Con/Nor
Con/Hpx
KO/Nor
KO/Hpx
IgG
IP:SETDB1
ZNF221
ZNF37A
2.0
1.5
1.0
0.5
0.0
5
4
3
2
1
0
***
***

B

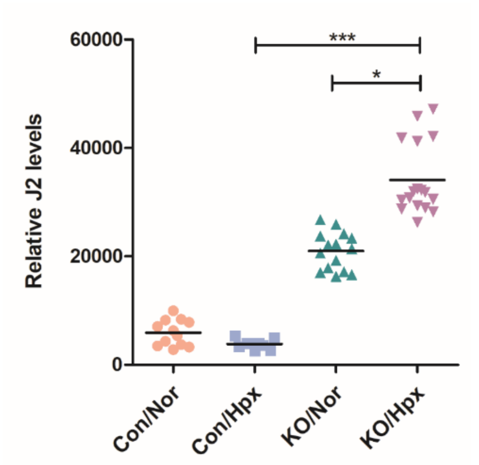
Relative J2 levels
60000
40000
20000
0
***
*
Con/Nor
Con/Hpx
KO/Nor
KO/Hpx

C

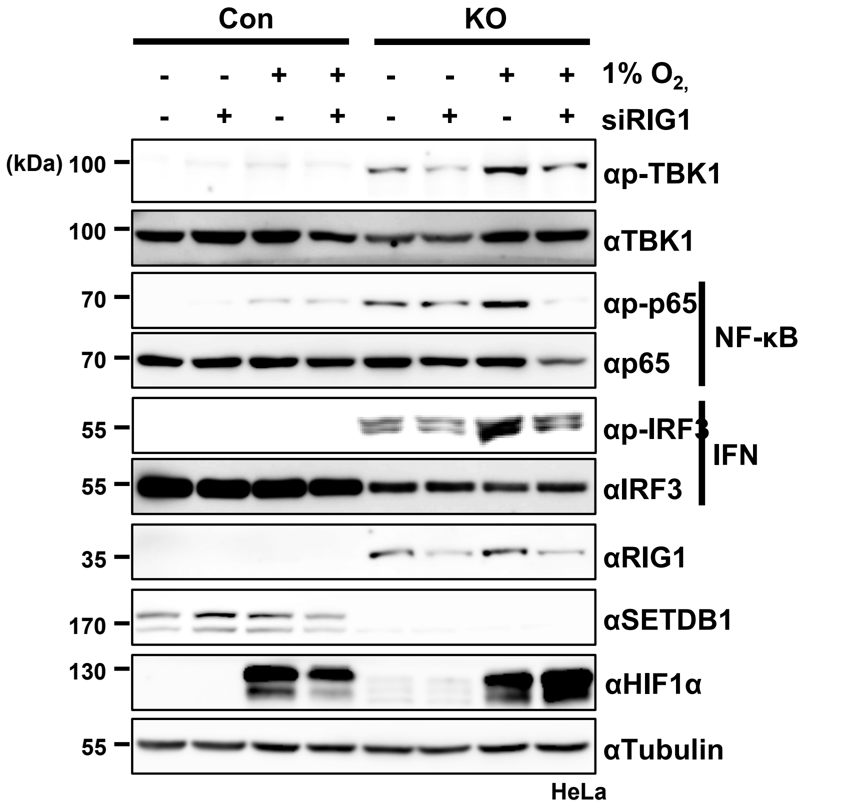
Con
KO
1% O2,
siRIG1
- - + + - - + +
- + - + - + - +
(kDa) 100
100
70
70
55
55
35
170
130
55
αp-TBK1
αTBK1
αp-p65
αp65
NF-κB
αp-IRF3
αIRF3
IFN
αRIG1
αSETDB1
αHIF1α
αTubulin
HeLa

Fig S7.

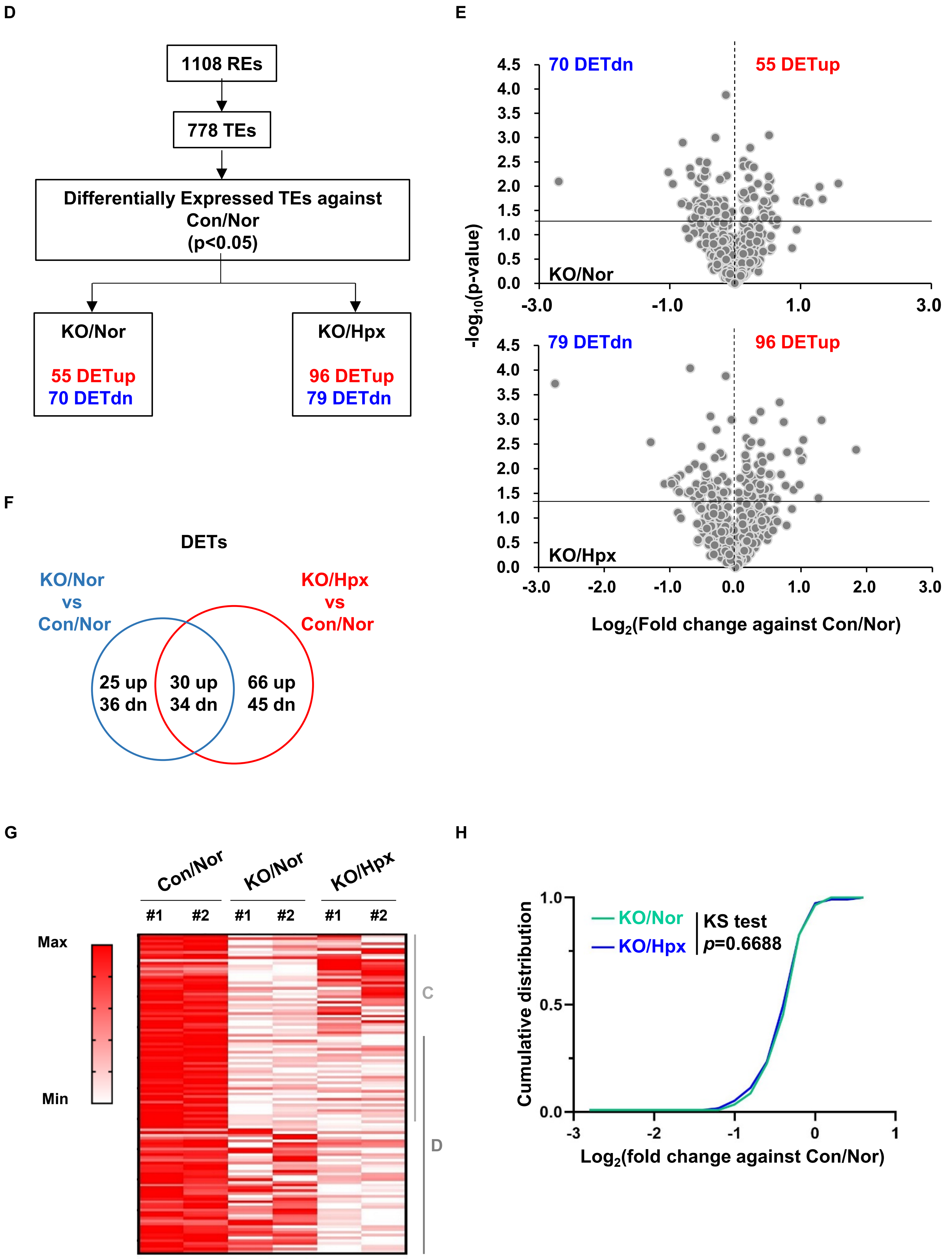

D
1108 REs
778 TEs
Differentially Expressed TEs against Con/Nor (p<0.05)
KO/Nor
55 DETup
70 DETdn
KO/Hpx
96 DETup
79 DETdn
E
70 DETdn
55 DETup
KO/Nor
79 DETdn
96 DETup
KO/Hpx
-log10(p-value)
Log2(Fold change against Con/Nor)
F
DETs
KO/Nor vs Con/Nor
KO/Hpx vs Con/Nor
25 up
36 dn
30 up
34 dn
66 up
45 dn
G
Con/Nor
KO/Nor
KO/Hpx
#1
#2
Max
Min
C
D
H
KO/Nor
KO/Hpx
KS test
p=0.6688
Cumulative distribution
Log2(fold change against Con/Nor)

Fig S7.

I

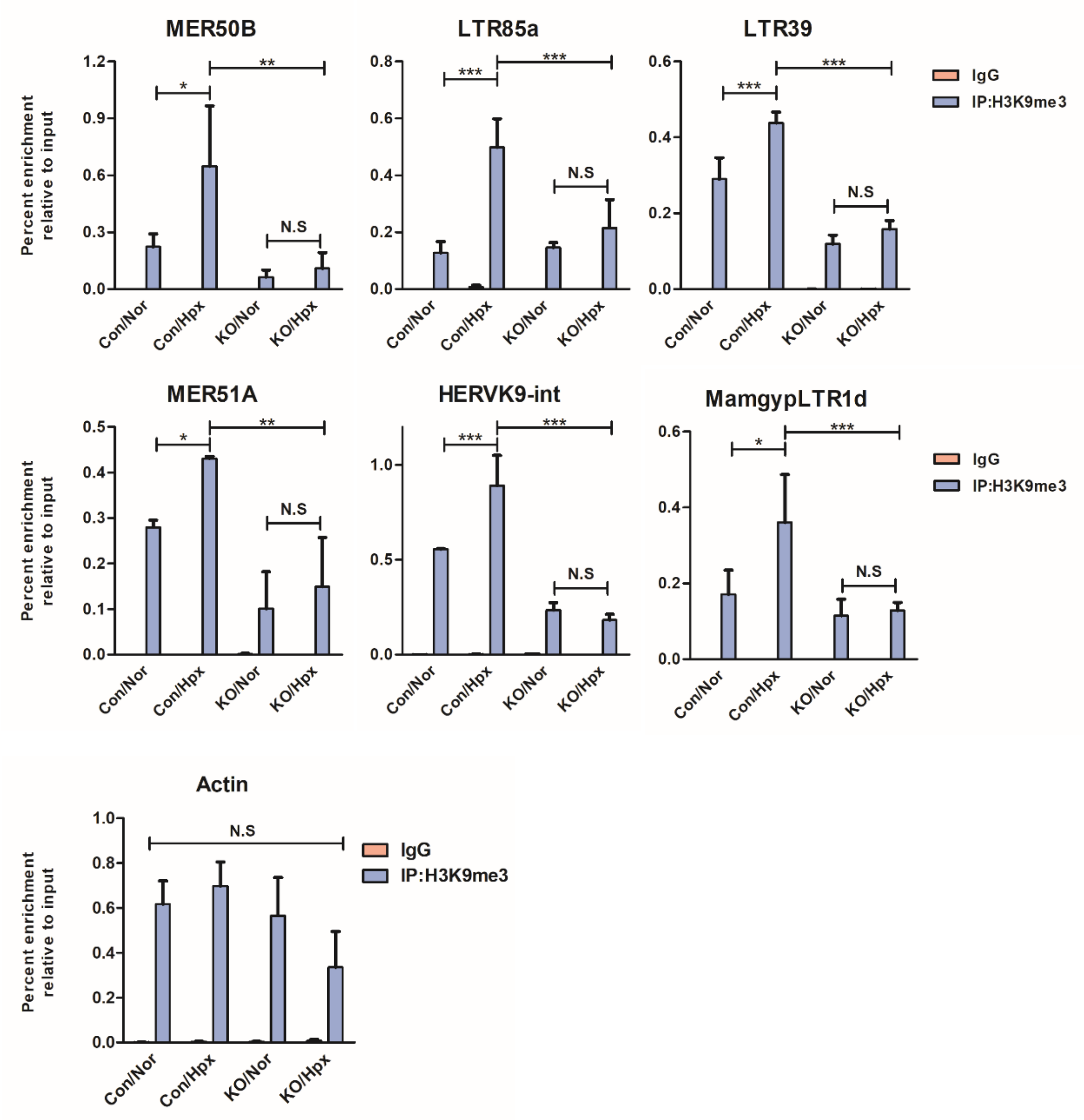
MER50B
LTR85a
LTR39
MER51A
HERVK9-int
MamgypLTR1d
Actin
Percent enrichment relative to input
Con/Nor
Con/Hpx
KO/Nor
KO/Hpx
N.S
IgG
IP:H3K9me3

K

J

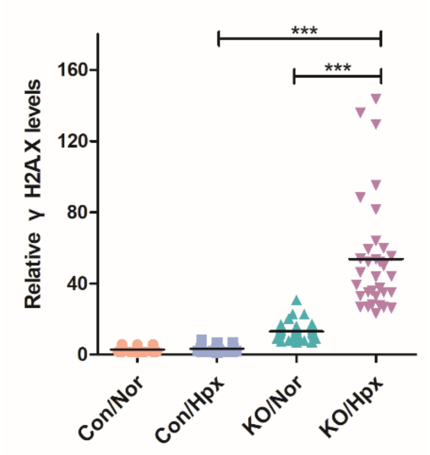
Relative γ H2A.X levels
Con/Nor
Con/Hpx
KO/Nor
KO/Hpx

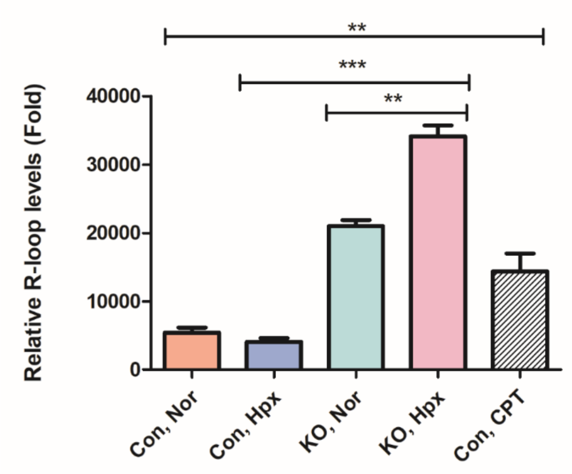
Relative R-loop levels (Fold)
Con, Nor
Con, Hpx
KO, Nor
KO, Hpx
Con, CPT

Fig S7.

L

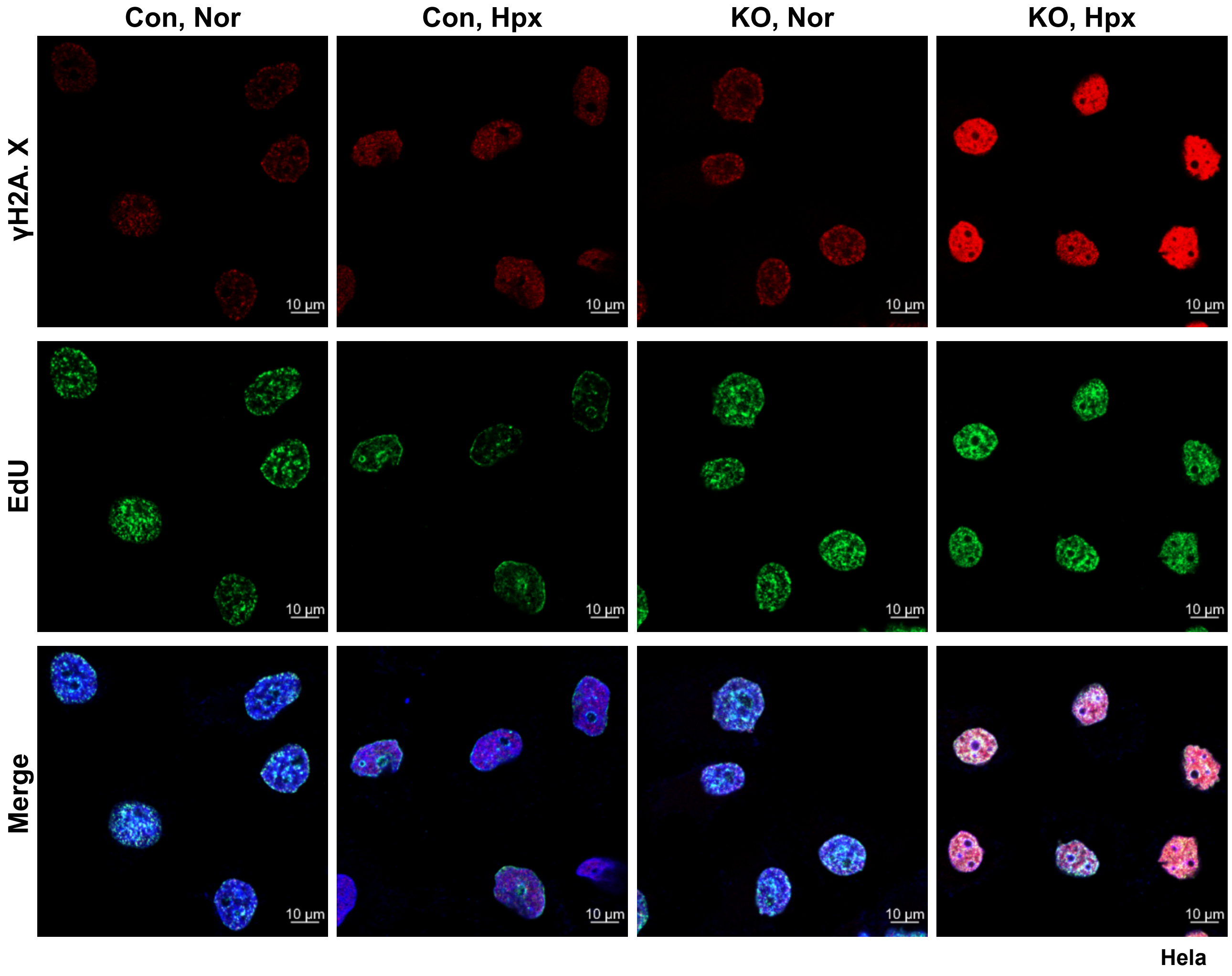
Con, Nor
Con, Hpx
KO, Nor
KO, Hpx
γH2A. X
EdU
Merge
10 μm
Hela

M

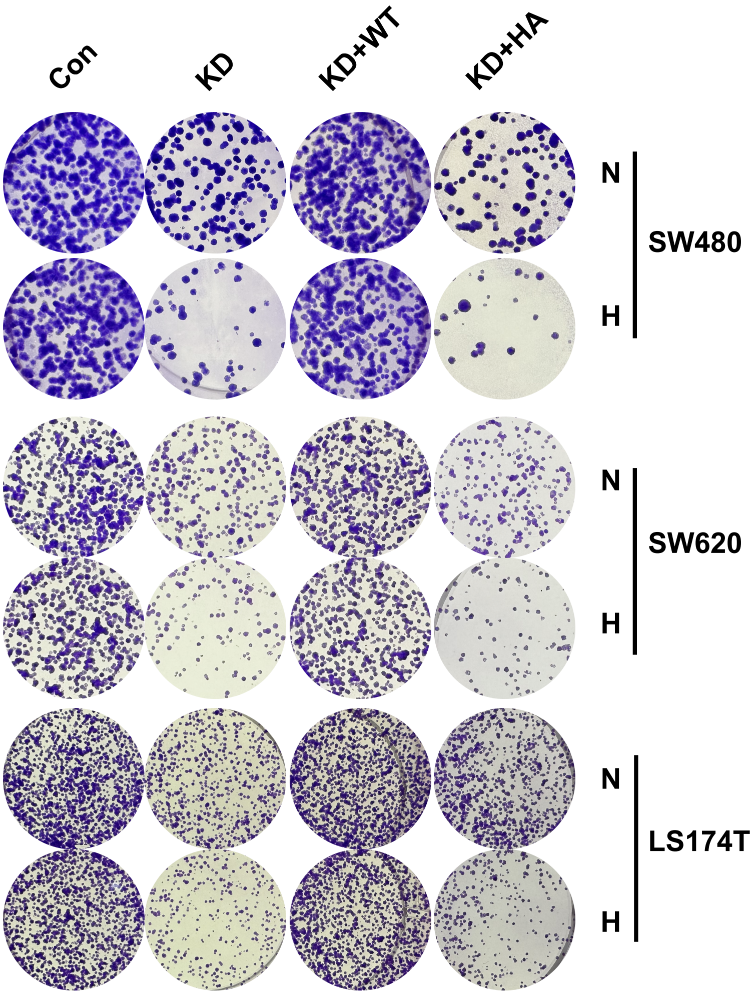
Con
KD
KD+WT
KD+HA
N
H
SW480
SW620
LS174T

N

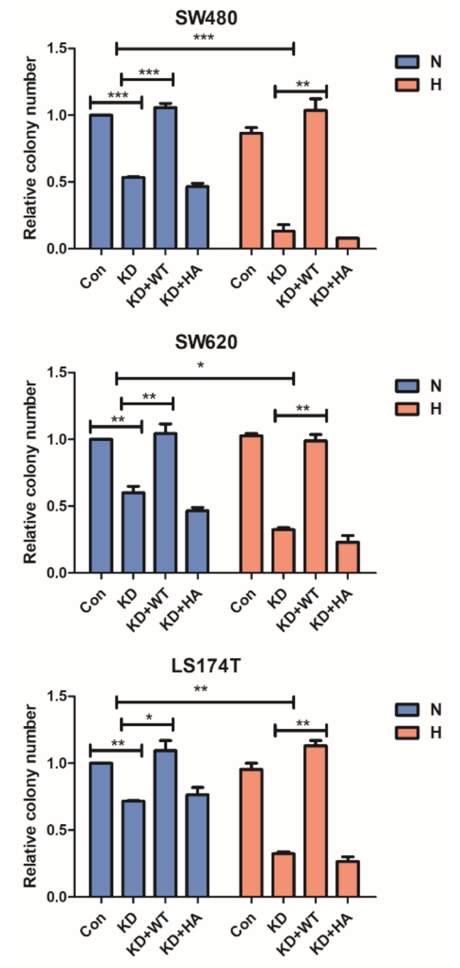
SW480
SW620
LS174T
Relative colony number
Con
KD
KD+WT
KD+HA
N
H

Fig S7.

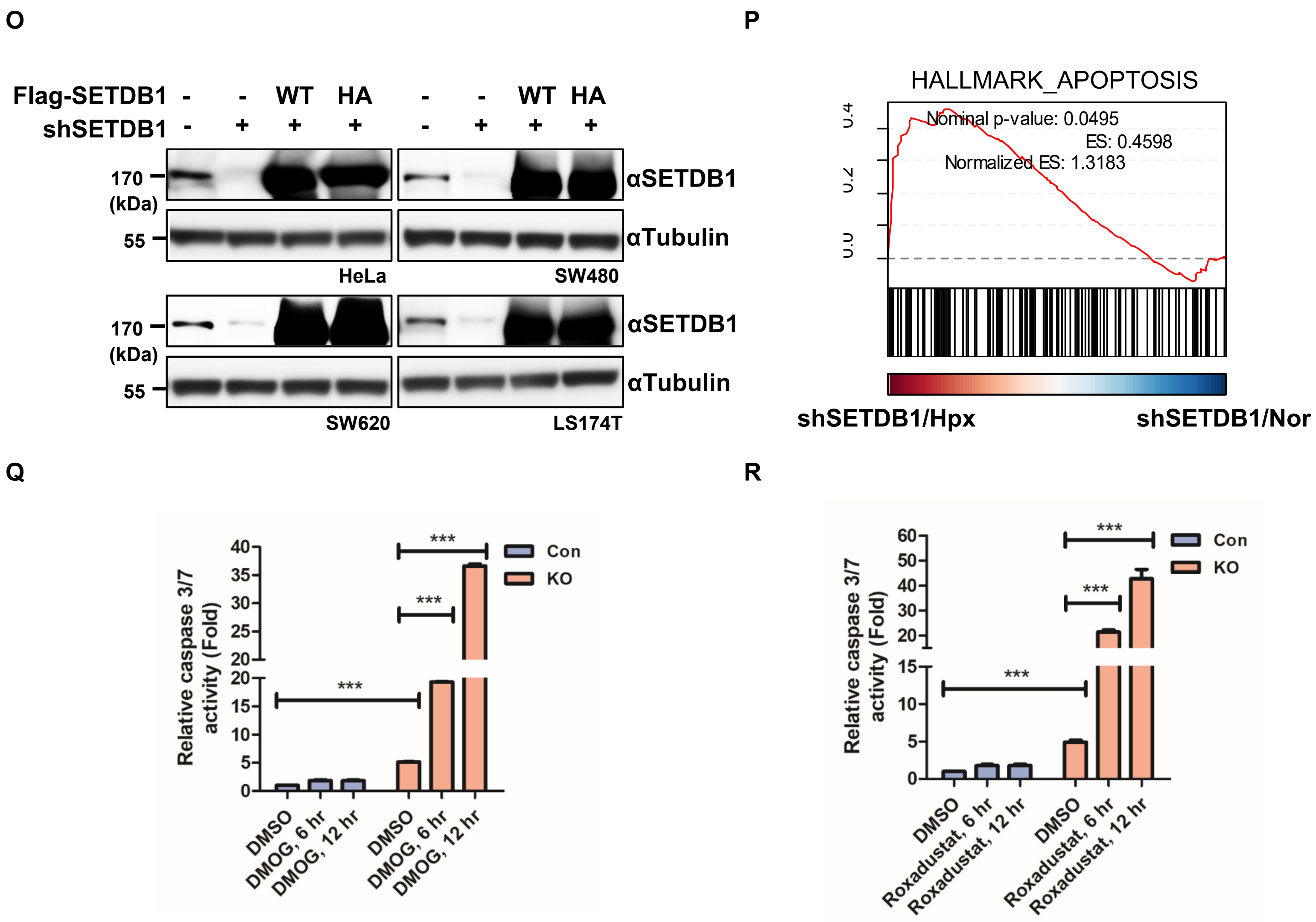
O
Flag-SETDB1 - - WT HA - - WT HA
shSETDB1 - + + + - + + +
170
(kDa)
55
αSETDB1
αTubulin
HeLa
SW480
SW620
LS174T
P
HALLMARK_APOPTOSIS
Nominal p-value: 0.0495
ES: 0.4598
Normalized ES: 1.3183
shSETDB1/Hpx
shSETDB1/Nor
Q
Relative caspase 3/7 activity (Fold)
Con
KO
DMSO
DMOG, 6 hr
DMOG, 12 hr
***
R
Relative caspase 3/7 activity (Fold)
Con
KO
DMSO
Roxadustat, 6 hr
Roxadustat, 12 hr
***

Fig S8.

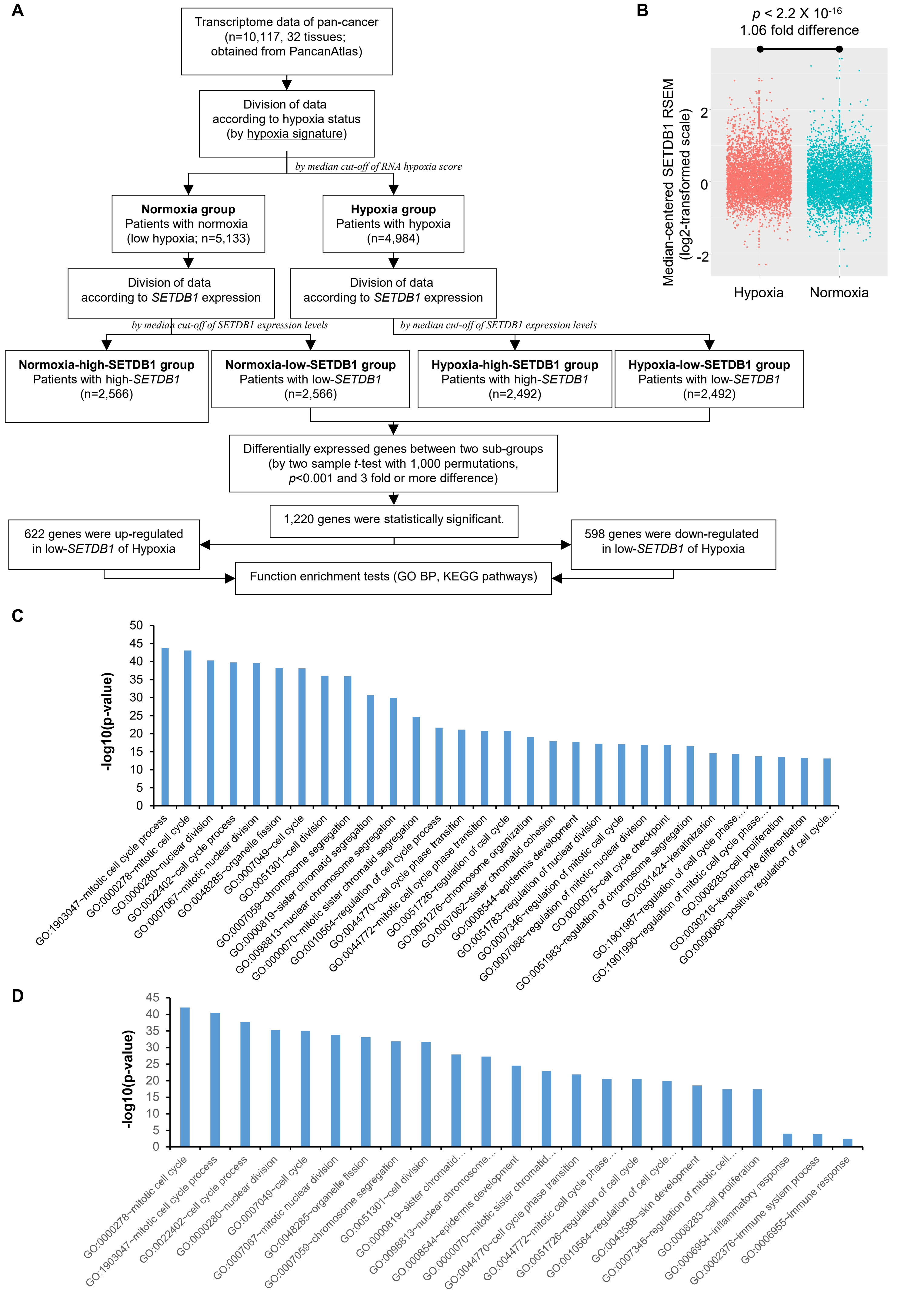
A
Transcriptome data of pan-cancer (n=10,117, 32 tissues; obtained from PancanAtlas)
Division of data according to hypoxia status (by hypoxia signature)
by median cut-off of RNA hypoxia score
Normoxia group Patients with normoxia (low hypoxia; n=5,133)
Hypoxia group Patients with hypoxia (n=4,984)
Division of data according to SETDB1 expression
Division of data according to SETDB1 expression
by median cut-off of SETDB1 expression levels
by median cut-off of SETDB1 expression levels
Normoxia-high-SETDB1 group Patients with high-SETDB1 (n=2,566)
Normoxia-low-SETDB1 group Patients with low-SETDB1 (n=2,566)
Hypoxia-high-SETDB1 group Patients with high-SETDB1 (n=2,492)
Hypoxia-low-SETDB1 group Patients with low-SETDB1 (n=2,492)
Differentially expressed genes between two sub-groups (by two sample t-test with 1,000 permutations, p<0.001 and 3 fold or more difference)
1,220 genes were statistically significant.
622 genes were up-regulated in low-SETDB1 of Hypoxia
598 genes were down-regulated in low-SETDB1 of Hypoxia
Function enrichment tests (GO BP, KEGG pathways)
B
p < 2.2 X 10-16
1.06 fold difference
Median-centered SETDB1 RSEM (log2-transformed scale)
2
0
-2
Hypoxia
Normoxia
C
-log10(p-value)
50
45
40
35
30
25
20
15
10
5
0
GO:1903047~mitotic cell cycle process
GO:0000278~mitotic cell cycle
GO:0000280~nuclear division
GO:0022402~cell cycle process
GO:0007067~mitotic nuclear division
GO:0048285~organelle fission
GO:0007049~cell cycle
GO:0051301~cell division
GO:0007059~chromosome segregation
GO:0000819~sister chromatid segregation
GO:0098813~nuclear chromosome segregation
GO:0000070~mitotic sister chromatid segregation
GO:0010564~regulation of cell cycle process
GO:0044770~cell cycle phase transition
GO:0044772~mitotic cell cycle phase transition
GO:0051726~regulation of cell cycle
GO:0051276~chromosome organization
GO:0007062~sister chromatid cohesion
GO:0008544~epidermis development
GO:0051783~regulation of nuclear division
GO:0007346~regulation of mitotic cell cycle
GO:0007088~regulation of mitotic nuclear division
GO:0000075~cell cycle checkpoint
GO:0051983~regulation of chromosome segregation
GO:0031424~keratinization
GO:1901987~regulation of cell cycle phase…
GO:1901990~regulation of mitotic cell cycle phase…
GO:0008283~cell proliferation
GO:0030216~keratinocyte differentiation
GO:0090068~positive regulation of cell cycle…
D
-log10(p-value)
45
40
35
30
25
20
15
10
5
0
GO:0000278~mitotic cell cycle
GO:1903047~mitotic cell cycle process
GO:0022402~cell cycle process
GO:0000280~nuclear division
GO:0007049~cell cycle
GO:0007067~mitotic nuclear division
GO:0048285~organelle fission
GO:0007059~chromosome segregation
GO:0051301~cell division
GO:0000819~sister chromatid…
GO:0098813~nuclear chromosome…
GO:0008544~epidermis development
GO:0000070~mitotic sister chromatid…
GO:0044770~cell cycle phase transition
GO:0044772~mitotic cell cycle phase…
GO:0051726~regulation of cell cycle
GO:0010564~regulation of cell cycle…
GO:0043588~skin development
GO:0007346~regulation of mitotic cell…
GO:0008283~cell proliferation
GO:0006954~inflammatory response
GO:0002376~immune system process
GO:0006955~immune response

Supplementary table S1

A

Cat No.	Antibody	Company	Application
ab107225	SETDB1	Abcam	PLA (1:100)
ab166917	Cullin2	Abcam	WB (1:1000)
ab113077	PHD1	Abcam	WB (1:1000)
ab133630	PHD2	Abcam	WB (1:1000)
ab30782	PHD3	Abcam	WB (1:1000)
ab76493	phospho-IRF3	Abcam	WB (1:1000)
ab109394	phospho-RPA32	Abcam	WB (1:1000)
ab1791	Histone H3	Abcam	WB (1:10000), ChIP
ab8898	H3K9me3	Abcam	WB (1:1000), ChIP
ab290	GFP	Abcam	WB (1:10000),
ab171870	IgG	Abcam	WB (1:1000), ChIP
A300-169A	ATF7IP (MCAF)	Bethyl Laboratories	WB (1:200)
#2196	SETDB1	CST	IF(1:100)
#68547	VHL	CST	WB (1:1000),
#2699	cullin4a	CST	WB (1:1000)
#14179	HIF1α	CST	WB (1:1000)
#3724	HA	CST	WB (1:1000)
#5537	HIF1β	CST	WB (1:1000)
#3504	TBK1	CST	WB (1:1000)
#11904	IRF3	CST	WB (1:1000)
#5483	phosepho-TBK1	CST	WB (1:1000)
#5321	MDA5	CST	WB (1:1000)
#3743	RIG1	CST	WB (1:1000)
#4764	p65	CST	WB (1:1000)
#3033	phospho-p65	CST	WB (1:1000)
#9718	γH2AX	CST	WB (1:1000)
#13934	ATR	CST	WB (1:1000)
#2873	ATM	CST	WB (1:1000)
#2853	phospho-ATR	CST	WB (1:1000)
#13050	phospho-ATM	CST	WB (1:1000)
#5625	Cleaved PARP	CST	WB (1:1000)
#9761	Cleaved Caspase-3	CST	WB (1:1000)
#9761	Cleaved Caspase-6	CST	WB (1:1000)
#2278	Myc-Tag	CST	WB (1:1000)
#7074S	anti-Rabbit IgG HRP conjugated	CST	WB (1:5000)
#2912	LDHA	CST	WB (1:5000)
#73812	Hydroxyproline	CST	WB(1:1000), IP
NB100-295	PHD4	Novus Biologicals	WB (1:1000)
11231-1-AP	SETDB1	Proteintech	WB (1:1000), ChIP
13538-1-AP	VHL	Proteintech	PLA (1:100)
34650	6X His	QIAGEN	WB (1:1000)
sc-133090	ElonginB	Santa Cruz Biotechnology	WB (1:1000)
sc-135895	ElonginC	Santa Cruz Biotechnology	WB (1:1000)
T5068	Tubulin	Sigma Aldrich	WB (1:10000)
F3165	Flag M2	Sigma Aldrich	WB (1:1000)
F2555	Flag (Rabbit)	Sigma Aldrich	WB (1:1000)
MABE1134	J2	Sigma Aldrich	IF (1:50)
MABE1095	R-loop (DNA-RNA Hybrid)	Sigma Aldrich	IF (1:50)
A11008	bit IgG (H+L) Cross-Adsorbed Secondary Antibody, Alexa	Thermo Fischer	IF (1:1000)
A11010	bit IgG (H+L) Cross-Adsorbed Secondary Antibody, Alexa	Thermo Fischer	IF (1:1000)
A11003	use IgG (H+L) Cross-Adsorbed Secondary Antibody, Alexa	Thermo Fischer	IF (1:1000)
A9044	anti-Mouse IgG HRP conjugated	Sigma-Aldrich	WB (1:5000)

WB; Western blot, IF; Immunofluorescence, PLA; Proximity ligation assay, IP; Immunoprecipitation

B

Application	Gene	Forward (5' - 3')	Reverse (5' - 3')
RT-qPCR	CXCL8	GAGAGTGATTGAGAGTGGACCAC	CACAACCCTCTGCACCCAGTTT
RT-qPCR	IL1A	TGTATGTGACTGCCCAAGATGAAG	AGAGGAGGTTGGTCTCACTACC
RT-qPCR	EBI3	TGTTCTCCATGGCTCCCTAC	AGAAGATCTCTGGGAAGGGC
RT-qPCR	IL4R	CTGCTCATGGATGACGTGGTCA	GGTGTGAACTGTCAGGTTTCCTG
RT-qPCR	GBP2	GTTCCTACATCCTCAGCCATTCC	CCACTGCTGATGGCATTGACGT
RT-qPCR	HLA-A	AGATACACCTGCCATGTGCAGC	GATCACAGCTCCAAGGAGAACC
RT-qPCR	RAET1L	AGAGCAACTGCTTGACATTCAGC	GAGTAGGAAGGTCTGTCCATCG
RT-qPCR	LTR39	CCCTTGCTTGTAATCCCTCA	AAACCTGGGTCTGCTTTCCT
RT-qPCR	HERVK9-int	GCAAGCTTTGAAGGAAATGC	TCAGCAGCCATTGTGCTATC
RT-qPCR	MER51A	ATTAGTCCCCTCCCACAACC	CATAGGGGCTCAAAGCTCAG
RT-qPCR	MER50B	CCACTCCTGCTCTGAAACTTG	CATATGGGTCTGCAGCAATC
RT-qPCR	LTR85a	TGGGGAAGGTGAAGCTTAGA	ACTCATTTGGCCATCAGACC
RT-qPCR	MamGypLTR1d	AATGCCAAACCTGTGTCCTC	TTCCTTTCTGGGCCCTCTAT
RT-qPCR	SETDB1	AAGAGTGTCTGCCCACAGGG	CATGTTTGGGTCACAATTGCA
RT-qPCR	Actin	CTCCTTAATGTCACGCACGAT	CATGTACGTTGCTATCCAGGC
ChIP-qPCR	CXCL8	AGTATGCCCCCTAAGAGCAGT	TGGTGAAGATAAGCCAGCCAA
ChIP-qPCR	IL1A	TCCTATGAGGGCATGGGTGA	GAGAACACCAGCCACCATCA
ChIP-qPCR	EBI3	AGAGGGTTGTCCAAGGAAGG	ACCCACAAAGTCCAGAGGGT
ChIP-qPCR	IL4R	GGCTGCATCCGGGTTCTTA	CACTCTGCAGGTAAGCAGGAG
ChIP-qPCR	ZNF221	AAGGCATGATTTCACCTTCA	CTGCCTCTTTGAATGTGGTC
ChIP-qPCR	ZNF37A	CATTCTGAAGAGGAACCTTCTG	TTTCTGATGCCAAATGAGGT
ChIP-qPCR	LTR39	AACCTAGAGCCCCACCAAAT	AGCAGGAGCTCACAGGTCAT
ChIP-qPCR	HERVK9-int	AGCGGGGATGGTAGGATTAC	TGGCATAATCAGGAGCTGTG
ChIP-qPCR	MER51A	CCAATTCACGCTGACTTCCT	GGTTGTGGGAGGGGACTAAT
ChIP-qPCR	MER50B	CTGCTCTGAAACTTGCCTGTC	CCATATGGGTCTGCAGCAAT
ChIP-qPCR	LTR85a	AGTGGTGCTGTGGAATGTGA	AACAAGGGGTTGTTCTCACG

C

Gene	Target Sequence (5' - 3')
sgSETDB1	GCUCUGAGGACGAAUCUUCC
shRNA/siRNA negative control	UUCUCCGAACGUGUCACGU
shSETDB1 (3' UTR)	AUCCCUCCCAUCCCAUAUUUG
shSETDB1	GCUCAGAUGAUAACUUCUGUA
shVHL	GGAGCGCAUUGCACAUCAACG
siMDA5	CCAACAAAGAAGCAGUGUAUA
siRIG1	CCAGAAUUAUCCCAACCGAUA
siARNT(HIF1β)	AAUGACAUCAGAUGUACCA
siPHD1	AAUGAGCAACCGGUCAAAG
siPHD2	UCUGAGUGUAGUAUGACUA
siPHD3	GUGCAGCCCUCUUACGCAA
siPHD4	CGAUGAAAUGAGUCUGAUU
siVHL #1	GGAGCGCAUUGCACAUCAACG
siVHL #2	UAUCACACUGCCAGUGUAUAC
siATF7IP	CCCUGUAUCCUAAGUGUUAAU

Supplementary table S2

A

Gene names	Score	Number of Oxidation (P)	Amino acid	Position in peptide	Position in protein	Charge	Sequence window	Oxidation (P) Probabilities
SETDB1	125.53	1	P	11	226	2	KTWHKGTLIAIQTVGPGKKYKVKFDNKGKSL	GTLIAIQTVGP(1)GKK
SETDB1	145.52	1;2	P	14	253	3	GKSLLSGNHIAYDYHPPADKLYVGSRVVAKY	SLLSGNHIAYDYHP(1)P(1)ADK
SETDB1	154.79	1;2	P	15	254	3	KSLLSGNHIAYDYHPPADKLYVGSRVVAKYK	SLLSGNHIAYDYHP(1)P(1)ADK
SETDB1	85.231	1	P	20	312	3	FDDGYASYVTQSELYPICRPLKKTWEDIEDI	FLIFFDDGYASYVTQSELYP(1)ICR
SETDB1	123.97	1;2	P	11	341	3	DISCRDFIEEYVTAYPNRPMVLLKSGQLIKT	DFIEEYVTAYP(1)NRP(1)MVLLK
SETDB1	139.08	1;2	P	14	344	3	CRDFIEEYVTAYPNRPMVLLKSGQLIKTEWE	DFIEEYVTAYP(1)NRP(1)MVLLK
SETDB1	104.35	1	P	4	430	3	QLRTRPNMGAVRSKGPVVQYTQDLTGTGTQF	SKGP(1)VVQYTQDLTGTGTQFK
SETDB1	314.56	1;2	P	1	496	3	AQSRKQVAKKSTSFRPGSVGSGHSSPTSPAL	P(1)GSVGSGHSSPTSPALSENVSGGK
SETDB1	232.2	1;2	P	11	506	3	STSFRPGSVGSGHSSPTSPALSENVSGGKPG	PGSVGSGHSSP(1)TSP(1)ALSENVSGGKPGINQTYR
SETDB1	232.2	1;2	P	14	509	3	FRPGSVGSGHSSPTSPALSENVSGGKPGINQ	PGSVGSGHSSP(1)TSP(1)ALSENVSGGKPGINQTYR
SETDB1	221.29	1;2;3	P	18	545	3	LGSTASAPAPSALPAPPAPPVFHGMLERAPA	SPLGSTASAPAPSALP(0.005)AP(0.992)P(0.992)AP(0.01)P(0.001)VFHGMLER
SETDB1	212.32	1;2;3	P	19	546	3	GSTASAPAPSALPAPPAPPVFHGMLERAPAE	SPLGSTASAPAPSALP(0.005)AP(0.992)P(0.992)AP(0.01)P(0.001)VFHGMLER
SETDB1	215.37	1;2;3	P	21	548	3	TASAPAPSALPAPPAPPVFHGMLERAPAEPS	SPLGSTASAPAPSALPAP(0.004)P(0.009)AP(0.993)P(0.995)VFHGMLER
SETDB1	235.73	1;2;3	P	22	549	3	ASAPAPSALPAPPAPPVFHGMLERAPAEPSY	SPLGSTASAPAPSALPAP(0.002)P(0.045)AP(0.955)P(0.998)VFHGMLER
SETDB1	88.224	1	P	5	575	3	AEPSYRAPMEKLFYLPHVCSYTCLSRVRPMR	LFYLP(1)HVCSYTCLSR
SETDB1	145.89	1	P	2	599	2	SRVRPMRNEQYRGKNPLLVPLLYDFRRMTAR	NP(1)LLVPLLYDFR
SETDB1	175.73	1	P	6	603	2	PMRNEQYRGKNPLLVPLLYDFRRMTARRRVN	NPLLVP(1)LLYDFR
SETDB1	131.04	1;2	P	15	755	3	KCACHQLTIQATACTPGGQINPNSGYQYKRL	CACHQLTIQATACTP(1)GGQINP(1)NSGYQYK
SETDB1	136.61	1;2	P	21	761	3	LTIQATACTPGGQINPNSGYQYKRLEECLPT	CACHQLTIQATACTP(1)GGQINP(1)NSGYQYK
SETDB1	114.27	1	P	7	775	3	NPNSGYQYKRLEECLPTGVYECNKRCKCDPN	RLEECLP(1)TGVYECNKR
SETDB1	231.66	1;2	P	6	1065	3	KMSVVTESSRNYGYNPSPVKPEGLRRPPSKT	NYGYNP(1)SP(1)VKPEGLR
SETDB1	290.28	1;2	P	8	1067	2	SVVTESSRNYGYNPSPVKPEGLRRPPSKTSM	NYGYNPSP(1)VKP(1)EGLR
SETDB1	322.99	1;2	P	11	1070	2	TESSRNYGYNPSPVKPEGLRRPPSKTSMHQS	NYGYNPSP(1)VKP(1)EGLR
SETDB1	146.53	1	P	10	1096	3	SMHQSRRLMASAQSNPDDVLTLSSSTESEGE	RLMASAQSNP(1)DDVLTLSSSTESEGESGTSR
SETDB1	188.33	1	P	2	1118	4	SSSTESEGESGTSRKPTAGQTSATAVDSDDI	KP(1)TAGQTSATAVDSDDIQTISSGSEGDDFEDKK
SETDB1	159.14	1	P	8	1228	3	KLEGNLGRYLNHSCSPNLFVQNVFVDTHDLR	YLNHSCSP(1)NLFVQNVFVDTHDLR
SETDB1	154.49	1	P	2	1245	2	LFVQNVFVDTHDLRFPWVAFFASKRIRAGTE	FP(1)WVAFFASK
NOTE. Putative hydroxylated proline sites were selected by oxidation (P) probabiities. Sites with probability <1 were excluded.								

B

	Normalized hydroxylated proline intensity					
	DMSO			DMOG		
	#1	#2	#2	#1	#2	#3
P575	0.003597588	0.004353763	0.003196034	0.003547332	0.002784024	0.004331632
P599	9.2851E-09	5.58892E-09	3.13108E-09	7.35747E-09	2.07996E-08	1.19756E-09
P603	0.000375119	0.000416592	0.000274284	0.000358129	0.000302209	0.000444416
P755	0.017087195	0.036361204	0.018297891	0.00950797	0.008935644	0.027867333
P761	0.000473913	0.00705437	0.001580862	0.000435378	0.001015277	0.00233271
P775	0.015653739	0.071947001	0.054909034	0.015287595	0.036999564	0.013005681
P1065	0.001442995	0.000195248	0.001786654	0.00168815	0.001044784	0.001410585
P1067	0.002753892	0.002973957	0.002301794	0.001865904	0.002745788	0.003235637
P1070	0.00500171	0.002257816	0.003667044	0.003377893	0.002415626	0.00273898
P1096	0.001269864	0.000824098	0.000229361	0.000600423	0.0003901	0.000228151
P1118	0.002253937	0.00044533	0.001442367	0.001884823	0.001825142	0.002249083
P1228	0.086015477	0.054397885	0.058367013	0.076285516	0.030046268	0.038974617
P1245	0.034207685	0.036566804	0.029713566	0.024914068	0.02222261	0.026693032

Supplementary Figure Legends

Figure S1.Hypoxia stabilizes SETDB1 protein.

(A) Half-life of Flag-SETDB1 protein in shControl (shCon) and shVHL HEK293T cells treated with cycloheximide (50 μg/mL) for the indicated times, determined by immunoblotting with the indicated antibodies. (B) Quantification of results in Figure S1A. (C, D) Effect of siRNA-mediated KD of VHL on polyubiquitination of ectopic Flag-tagged SETDB1 in HEK293T cells treated with bortezomib (1 μM, 12 h), measured by immunoblotting with the indicated antibodies. (E) Upregulation of ubiquitination of overexpressed Flag-tagged SETDB1 by overexpression of Myc-tagged VHL in HEK293T cells treated with bortezomib (1 μM) for 12 h.

Figure S2. Stability of SETDB1 and its binding partner ATF7IP are dependent on oxygen availability.

(A) Inverse correlation of SETDB1 protein expression and oxygen concentration in HEK293T and HeLa cells exposed to different O_2 levels for 3 h, determined by immunoblotting with the indicated antibodies. (B) Quantification of SETDB1 immunofluorescent signals in cells examined for Figure 2B (n >40, *** *p*-value < 0.001, Student's t-test). (C) SETDB1 mRNA levels in HeLa cells under normoxia or hypoxia (1% O_2) for the indicated times measured via qRT-PCR. Relative levels were calculated by normalizing to actin mRNA. (D) Immunoblot analysis of SETDB1 and ATF7IP in HeLa cells treated with 2,2-bipyridyl (200 μM) for the indicated times. (E)

SETDB1 protein levels in HeLa cells exposed to different hypoxic conditions. Cells exposed to hypoxic conditions (1% O_2, 3 or 6 h) were rescued under normoxic conditions (Re-O_2) for the indicated times and SETDB1 levels were assessed by immunoblotting. (F) Immunoblot analysis of nuclear SETDB1 levels in HeLa cells with siRNA-mediated knockdown of ATF7IP (siATF7IP) under normoxia or hypoxia (1% O_2, 6 h).

Figure S3. Hypoxia-induced SETDB1 stabilization in different cell lines.

(A) Immunoblot analysis of SETDB1 protein levels in HDFn and HEKa cells under hypoxia. (B-G) Immunoblot analysis of SETDB1 protein levels in cancer cell lines under hypoxia (1% O_2 for the indicated times) or hypoxia mimetic conditions (200 μM 2,2-bipyridyl) for the indicated times. (H) Immunoblot analysis of SETDB1 protein levels in HeLa cells under hypoxic stress (1% O_2, 6 h) or with DMOG (1 mM) or roxadustat (100 μM, 6 h) treatment. (I) Immunoblot analysis of SETDB1 protein levels in RCC4 cells treated with DMOG (1 mM) for the indicated times. (J) SETDB1 protein levels in different cell cycle phase, determined by immunoblot analysis of lysates of cells released from nocodazole (100 ng/ml) arrest in the presence of DMOG (1 mM) or DMSO control. (K) mRNA levels of PHDs in Figure 2G, measured by qRT-PCR. Relative levels were calculated by normalizing to actin mRNA. (L) Additive effect of simultaneous depletion of PHD1-4 on SETDB1 levels in HeLa cells, determined by immunoblotting using the indicated antibodies. Non-targeting siRNA was used as a negative control.

Figure S4. Physical interactions of SETDB1 with PHDs.

(A-D) Analysis of physical interactions of SETDB1 and overexpressed PHD1-4. Endogenous SETDB1 and overexpressed Flag-PHD1 (A), GFP-PHD2 (B), Flag-PHD3 (C), and Flag-PHD4 (D) were immunoprecipitated using the indicated antibodies, followed by immunoblotting.

Figure S5. VHL recognizes hydroxylated proline residues on SETDB1.

(A) Hydroxylation at proline residues on Flag-tagged SETDB1 and HA tagged HIF1α examined via an *in vitro* hydroxylation assay utilizing recombinant PHD1 and PHD3 (PHDs), followed by immunoblotting with the indicated antibodies. (B) Hydroxylated proline residues of HIF1α in HEK293T cells with the indicated with DMOG (1 mM) treatment, captured by immunoprecipitation followed by immunoblotting with the indicated antibodies. (C) Flag-tagged, hydroxylated proline-containing SETDB1 in HEK293T cells treated with DMOG (1 mM, 12 h) or 2,2-bipyridyl (200 μM, 12 h), or exposed to hypoxia (1% O_2,12 h), captured by immunoprecipitation with an anti-OH antibody or anti-Flag antibody followed by immunoblotting with the indicated antibodies. (D) Schematic imaging of SETDB1 truncation mutants. (E) Physical interactions between HA-VHL and T1, T2, or T3 mutant SETDB1 in HEK293T cells determined via immunoprecipitation using an anti-Flag antibody followed by immunoblotting with the indicated antibodies. (F) Physical interactions between HA-VHL and T4, T5, or T6 mutant SETDB1 in HEK293T cells determined via immunoprecipitation using anti-Flag antibody followed by immunoblotting with the

indicated antibodies. (G) VHL-binding, hydroxylated proline residues of SETDB1, identified by mass spectrometry. The intensity of each hydroxylated proline of SETDB1 was calculated by normalizing the indicated hydroxylated proline residue-containing peptide to that of total peptides from SETDB1. (H) Mass spectra of the indicated non-hydroxylated and hydroxylated prolines in SETDB1. (I-L) Physical interactions between diverse mutant SETDB1 (P575A, P755A, P775, P1228A, or P1245A) and HA-VHL in HEK293T cells determined by immunoprecipitation using anti-Flag antibody followed by immunoblotting with the indicated antibodies. (M) Half-lives of wild-type (WT) and mutant SETDB1 with 3PA (3PA) in HEK293T treated with cycloheximide (CHX, 50 µg/ml) for the indicated times analyzed by immunoblotting with the indicated antibodies. (N) Quantitative analysis of half-lives of WT and 3PA SETDB1 proteins. Data are expressed as means ± SD of three independent experiments (* p-value < 0.05, Student's t-test). (O) Effect of DMOG (1 mM) treatment on the histone H3K9 methylation activity of Flag-tagged SETDB1, assessed by *in vitro* methylation assay using recombinant core histone (1.5 µg) followed by immunoblotting with the indicated antibodies. (P) Effects of the 3PA mutation on histone H3K9 methylation activity of Flag-tagged SETDB1 (0.5 µg) examined via an *in vitro* methylation assay utilizing recombinant core histone (1.5 µg) followed by immunoblotting with the indicated antibodies.

Figure S6. Immune-inflammatory response is overrepresented in SETDB1 knockout cells in hypoxia.

(A) Multidimensional scaling (MDS) plot of RNA-seq data from HeLa cells depleted of

SETDB1 (shSETDB1) under hypoxic (hpx) versus normoxic (nor) conditions, and in control HeLa cells (shControl) under hypoxic versus normoxic conditions. Each point represents 1 sample. (B) Gene ontology (GO) over-representation in differentially upregulated genes in SETDB1 knockout (KO) HeLa cells under hypoxia compared with control cells under normoxia. Immune-inflammatory response-related GO are presented in orange. (C) Venn diagram displaying gene ontology-biological pathways (GO_BP) identified with gene set enrichment analysis (GSEA). Pathways enriched in HeLa cells depleted of SETDB1 under hypoxia compared with those under normoxia are presented in red. Pathways enriched in control HeLa cells under hypoxia compared with those under normoxia are presented in black. (D) Heatmap showing relative expression of differentially upregulated innate immune signature-related genes in SETDB1 knockout (KO) cells under hypoxia.

Figure S7. Hyperactivated innate-inflammatory response in SETDB1 knockout cells under hypoxia is dependent on derepression of a subset of TEs.

(A) Occupancy of SETDB1 at promoters of different inflammatory response genes and known SETDB1-associated loci (ZNF221 and ZNF37A) analyzed using ChIP-qPCR. (B) Quantification of anti-J2 signal intensity as described in Figure 5A (* p-value < 0.05, ** p-value < 0.005, Student's t-test). (C) Inflammatory signaling activities in RIG1-depleted (siRIG1) cells determined via immunoblot with the indicated antibodies. KO: SETDB1 KO, Con: control. (D) Schematic diagram of analysis of transcripts derived from transposable elements (TE) in KO cells under normoxia (Nor) and hypoxia (Hpx). RE: repetitive element, DETup: differentially upregulated TEs (red), DETdn:

differentially downregulated TEs (blue). (E) Volcano plots displaying $\log_2$ fold change of TEs in KO/Nor and KO/Hpx compared with those in Con/Nor. (F) Venn diagram of DETs identified in KO/Nor and KO/Hpx compared with those in Con/Nor ($p<0.05$). (G) Heatmap displaying relative expression of DETdns in KO/Nor (C) and KO/Hpx (D). (H) Cumulative distribution of fold changes in transcript abundance for TE groups C and D in SETDB1 KO cells under normoxia (KO/Nor) and hypoxia (1% O_2, 12 h; KO/Hpx) compared with those in control cells under normoxia (Con/Nor). The *p*-values were calculated with the Kolmogorov Smirnov (KS) test. (I) ChIP-qPCR analysis of H3K9me3 enrichment at the indicated TE and Actin loci in SETDB1 KO (KO) and control (Con) HeLa cells under either normoxia or hypoxia (1% O_2, 6 h). Results are expressed as fold enrichment relative to input DNA and presented as mean ± SEM (n = 3, ** *p*-value < 0.005, *** *p*-value < 0.001, Student's t-test). (J) Quantified γH2AX signal intensities assessed as for Figure 6A (n>30, *** *p*-value < 0.001, Student's t-test). (K) Quantification of relative R-loop intensity as described in Figure 6C (* *p*-value < 0.05, ** *p*-value < 0.01, *** *p*-value < 0.001, Student's t-test). (L) Representative immunofluorescent images of anti-γH2A antibodies (red), EdU (green), and DAPI (blue) in KO and Con cells under normoxia and hypoxia (1% O_2, 24 h). (M) Colony formation assay (CFA) in SETDB1-depeted (KD), control (Con), Wild-type (WT) SETDB1-rescued (Flag-SETDB1 overexpressing) and catalytic dead H1224A (HA) SETDB1-reintroduced colon cancer cells exposed to hypoxia mimetic condition (2,2-bipyridyl, 200 μM for 24 h) followed by normoxic culture (medium change) for 24 d. N: normoxic condition; H: hypoxia mimetic condition. (N) Quantification of relative colony number as described in Figure S7M (* *p*-value < 0.05, ** *p*-value < 0.01, *** *p*-value < 0.001, Student's t-test). (O) Western blot analysis of knockdown of SETDB1 and re-introduction of wildtype (WT) and catalytic dead H1224A mutant (HA) SETDB1 (Flag-

SETDB1 overexpressing) in SW480, SW620 and LS174T colon cancer cells using the indicated antibodies. (P) GSEA displaying overrepresentation of the 'hallmark_apoptosis' pathway in SETDB1-depleted (shSETDB1) HeLa cells under hypoxia compared with shSETDB1 under normoxia. (Q, R) Caspase 3/7 activity in SETDB1-KO (KO) and control HeLa cells exposed to DMOG (1 mM) or roxadustat (100 μM) for the indicated times, assessed with a Caspase-Glo 3/7 Assay.

Figure S8. Transcriptomic profiling of pan-cancer patient data

(A) Overview of transcriptomic data of clinical samples. (B) Expression of the SETDB1 transcript in clinical samples harboring hypoxia and normoxia signatures. (C) Overview of GO pathways overrepresented in the Hypoxia group relative to the Normoxia group. (D) Overview of GO pathways in the Hypoxia-low-SETDB1 group compared with the Normoxia-low-SETDB1 group.

Supplementary table S1.

(A) List of antibodies used in the study. (B) Sequence of primers used for RT-PCR studies (C) Target sequences of siRNA oligonucleotides.

Supplementary table S2.

(A) List of hydroxylated proline containing peptides in SETDB1 identified from LC-MS analysis. (B) Normalized intensity of hydroxylated proline residues on SETDB1 with DMOG (1 mM, 6 h) or DMSO control. Relative insensities were calculated by

normalizing to that of total peptides from SETDB1.
